# CRISPR/Cas9-mediated targeted mutagenesis of *TAS4* and *MYBA7* loci in grapevine rootstock 101-14

**DOI:** 10.1007/s11248-020-00196-w

**Published:** 2020-04-23

**Authors:** Sukumaran Sunitha, Christopher D. Rock

**Affiliations:** Department of Biological Sciences, Texas Tech University, Lubbock, TX 79409-3131 USA

**Keywords:** MYB transcription factor, microRNA, Flavonoids, Genome editing, Off-target editing, RNA interference, Genetically modified organisms

## Abstract

Pierce’s disease (PD) of grapevine (*Vitis vinifera*) is caused by the bacterium *Xylella fastidiosa* and is vectored by xylem sap-sucking insects, whereas *Grapevine Red Blotch Virus* (GRBV) causes Red Blotch Disease and is transmitted in the laboratory by alfalfa leafhopper *Spissistilus festinus*. The significance of anthocyanin accumulations in distinct tissues of grapevine by these pathogens is unknown, but vector feeding preferences and olfactory cues from host anthocyanins may be important for these disease etiologies. Phosphate, sugar, and UV light are known to regulate anthocyanin accumulation via miR828 and *Trans*-*Acting Small*-*interfering locus4* (*TAS4*), specifically in grape by production of phased *TAS4a/b/c* small-interfering RNAs that are differentially expressed and target *MYBA5/6/7* transcription factor transcripts for post-transcriptional slicing and antisense-mediated silencing. To generate materials that can critically test these genes’ functions in PD and GRBV disease symptoms, we produced transgenic grape plants targeting *TAS4b* and *MYBA7* using CRISPR/Cas9 technology. We obtained five *MYBA7* lines all with bi-allelic editing events and no off-targets detected at genomic loci with homology to the guide sequence. We obtained two independent edited *TAS4b* lines; one bi-allelic, the other heterozygous while both had fortuitous evidences of bi-allelic *TAS4a* off-target editing events at the paralogous locus. No visible anthocyanin accumulation phenotypes were observed in regenerated plants, possibly due to the presence of genetically redundant *TAS4c* and *MYBA5/6* loci or absence of inductive environmental stress conditions. The editing events encompass single base insertions and di/trinucleotide deletions of *Vvi*-*TAS4a/b* and *Vvi*-*MYBA7* at expected positions 3 nt upstream from the guideRNA proximal adjacent motifs NGG. We also identified evidences of homologous recombinations of *TAS4a* with *TAS4b* at the *TAS4a* off-target in one of the *TAS4b* lines, resulting in a chimeric locus with a bi-allelic polymorphism, supporting independent recombination events in transgenic plants associated with apparent high Cas9 activities. The lack of obvious visible pigment phenotypes in edited plants precluded pathogen challenge tests of the role of anthocyanins in host PD and GRBV resistance/tolerance mechanisms. Nonetheless, we demonstrate successful genome-editing of non-coding RNA and *MYB* transcription factor loci which can serve future characterizations of the functions of *TAS4a/b/c* and *MYBA7* in developmental, physiological, and environmental biotic/abiotic stress response pathways important for value-added nutraceutical synthesis and pathogen responses of winegrape.

**Electronic supplementary material:**

The online version of this article (10.1007/s11248-020-00196-w) contains supplementary material, which is available to authorized users.

## Background

Historically, plant diseases have been controlled by the application of chemical pesticides, commonly leading to residual contamination, negative impacts on beneficial insects, and vector insecticide resistance (Stelinski et al. 2012). Host pathogen resistance and crop quality improvements depends on applying new genetic insights and new technologies to accelerate breeding through improved genotyping and phenotyping methods, and by exploiting the available diversity in germplasm. The genetic identity of traditional grapevine (*Vitis vinifera*) cultivars used for wine discourages breeding approaches because markets and appellation statutes dictate cultivar choice, thus varieties lack recombination (Myles et al. 2011) and the resultant opportunity to select/screen for adaptability. Genome editing technologies, on the other hand, can result in non-“genetically modified organisms” (GMO) after outcrossing the effector transgene locus. Recently the USDA issued a directive that the agency does not have plans to regulate plants generated using gene editing techniques that create deletions/insertions that could otherwise have been developed through traditional breeding techniques (https://www.usda.gov/media/press-releases/2018/03/28/secretary-perdue-issues-usda-statement-plant-breeding-innovation), expanding prospects for genome editing of crops for resistance to insect pests and pathogens (Bisht et al. 2019; Mushtaq et al. 2019).

Two grapevine pathogens in particular [*Grapevine Red Blotch Virus* (GRBV) and *Xylella fastidiosa* (XF)] cause host disease symptoms that implicate anthocyanin as effectors that could mediate disease spreads in vineyards. There is evidence for host plant stress physiology associated with disease vector feeding deterrence in grapevine (Krugner et al. 2012). Some anthocyanin and derivative tannic compounds can reduce insect feeding (Johnson et al. 2010), including sap-sucking insects (Barbehenn and Constabel 2011; Makoi et al. 2010), which provides a plausible basis for observed XF infection susceptibility differences between anthocyanless and red cultivars (Cantos et al. 2002; Krivanek and Walker 2004; Raju and Goheen 1981). Prior work demonstrated that XF infection causes a significant decrease in leaf elemental phosphorus content of leaves (De La Fuente et al. 2013), and anthocyanin accumulation is a well-known plant physiological response to inorganic phosphate (P_i_) starvation or sucrose treatment, including grapevine (Yamakawa et al. 1983; Yin et al. 2012). Based on their mobile nature in vascular tissues, P_i_, sugars, the plant stress hormone abscisic acid (ABA), microRNAs (miRNAs), and target mRNAs have been recognized as systemic signals that convey the whole-plant P_i_ status internally (Lin et al. 2008, 2018; Thieme et al. 2015). Phytoalexin polyphenolics accumulate in xylem sap and leaves of XF-infected almonds (Wilhelm et al. 2011) and grape (Wallis and Chen 2012); some cultivars (e.g. ‘Rubired’) induce polyphenolics to higher concentrations and do not develop PD symptoms as quickly as anthocyanless cultivars such as ‘Chardonnay’ or ‘Thompson Seedless’ (Wallis et al. 2013). Phenolics inhibit XF growth in vitro (Maddox et al. 2010), and foliar applications of ABA increase xylem sap polyphenolics and promote curing of XF-infected grapevines in the greenhouse (Meyer and Kirkpatrick 2011).

*Grapevine Red Blotch Virus* (GRBV) is a monopartite, grapevine-infecting Grablovirus causing Red Blotch Disease and was first observed in California in 2008 (Calvi 2011). Bahder et al. (2016) identified the alfalfa leafhopper *Spissistilus festinus* as the candidate vector that can transmit GRBV under laboratory conditions. GRBV disease symptoms manifest as red patches due to anthocyanin accumulation in the middle of the grapevine leaf and in veins and petiole, which coalesce at the end of the growing season (Sudarshana et al. 2015). GRBV infection results in delayed and uneven berry ripening, higher titratable acids, reduced sugar and reduced anthocyanin content in the berry (Oberhoster et al. 2016), impairing fruit qualities which threaten both table grape and wine industries (Rwahnih et al. 2015).

XF is a gram-negative, xylem-limited bacterium associated with a large number of crop diseases (Kyrkou et al. 2018) including Pierce’s disease of grape (PD), alfalfa dwarf, phony peach disease, plum leaf scald, citrus variegated chlorosis, leaf scorches of coffee, almond, mulberry, blueberry, and most recently Olive quick decline syndrome in Italy (Almeida and Nunney 2015). PD is vectored by xylem sap-sucking insects, in particular the Glassy-Winged Sharpshooter (GWSS; *Homalodisca vitripennis*), an invasive species that caused an epidemic of PD in southern California in the 1990s, and by the endemic blue-green sharpshooter *Graphocephala atropunctata* in the Pacific northwest and northern California. Obvious PD symptoms are anthocyanin accumulation in leaves at the scorched periphery and shriveling of berries that impacts fruit quality and yield. The threat of a PD epidemic in northern California and the Pacific Northwest like in southern and central California in the 1880s, 1930s, 1970s, and 1990s remains real.

New tools and management strategies are needed to combat grapevine diseases. Despite years of focused efforts by microbiologists, entomologists, and plant physiologist/pathologists, the molecular mechanisms of PD or GRBV disease etiology are not understood (Kyrkou et al. 2018; Yepes et al. 2018). We hypothesize that *Trans*-*acting small*-*interfering RNA locus4* (*TAS4)* (Rajagopalan et al. 2006) is a molecular determinant of GRBV and PD host susceptibility. *TAS4* generates a ~1 kb long non-coding RNA spawning ‘phased’ siRNAs (phasiRNAs) in 21 nt register due to processive activity of DICER-LIKE4 (DCL4) triggered by miR828. miR828 is a P_i_- (Hsieh et al. 2009) and ABA-regulated (Luo et al. 2012) miRNA that directly and indirectly targets MYeloBlastosis viral oncogene-like (*MYB)* transcription factors (Rajagopalan et al. 2006) *PRODUCTION OF ANTHOCYANIN1/PAP1/MYB75/Vvi*-*MYBA6*, *PAP2/MYB90/Vvi*-*MYBA7*, and *MYB113/Vvi*-*MYBA5* (Sunitha et al. 2019). Grapevine has one *MIR828* and three functionally conserved *TAS4* loci (a–c) with implications for differential MYB cleavage activities (Rock 2013; Sunitha et al. 2019). Although genome editing of animal non-coding RNAs has been demonstrated and the method has high potential for engineering crops (Basak and Nithin 2015), only one report to date describes CRISPR editing of a plant non-coding RNA involved in tomato ripening (Li et al. 2018). We have applied Clustered Regularly Interspaced Short Palindromic Repeats/Cas9 (CRISPR/Cas9) genome editing technology (Cong et al. 2013; Jinek et al. 2012) to disrupt grapevine *Vvi*-*TAS4a/b* and *Vvi*-*MYBA7* host genes to enable future critical assessments of candidate effectors of PD and GBRV etiology.

## Materials and methods

### Plasmid construction

We obtained binary plasmid p201N-Cas9 (Jacobs et al. 2015; Jacobs and Martin 2016) (www.addgene.org plasmid #59175) and generated recombinant vectors using the NEBuilder HiFi DNA Assembly Cloning Kit (New England Biolabs) to genome edit the, *VviTAS4b*, and its target *VviMYBA6/7* loci (Table 1). Specifically, 20 bp guide sequences for *Tas4b*, and *MYBA6* and *MYBA7* were mined (Liu et al. 2017) to minimize off-target potential (Bae et al. 2014), comprising G(N)_19_ synthetic guide (sgRNA) upstream from a protospacer adjacent motif NGG and distal scaffold sequence for Cas9 activity (Jinek et al. 2012).

**Table 1:** Synthetic guide sequences for CRISPR-Cas9 editing of *VviMYBA6*, *MYBA7*, and *TAS4b* genes

Gene.test	Candidate guide sequence	Relative genome position	Off targets, seed (12)NGG? (seed mismatches, microhomology score; locus)
VviMYBA6.1	GGCCCTTCAGGAGTGCGGAA	Exon1, codon3, sense	No
VviMYBA7.1	GGCTCTTTAGGTCTGCGGAA	Exon1, codon3, sense	chr7:14830652 (2 mm, 0.4; intergenic)
VviTAS4b.2	CGGACCTTCACCATGGCCAC	D4 phase, sense	chr14:21607930rc (1 mm, 1.5; *TAS4a*)

Prioritized candidates were chosen based on dearth of canonical off targets with low seed microhomology scores (Bae et al. 2014; Liu et al. 2017)

### Agrobacterium-mediated grapevine rootstock 101-14 transformation

Recombinant vectors electroporated into *Agrobacterium* EHA105 (plus empty vector control) were used to transform embryogenic callus derived from anthers of commercially relevant grapevine rootstock 101-14 (which produces requisite marker anthocyanins for phenotyping) as fee-for-service from the UC Davis Plant Transformation Facility (Tricoli et al. 2014). Regenerated plants were shipped under USDA permit APHIS-BRS# 17-342-101m.

### Genomic Southern blot analysis of transgene events

Total DNA was extracted from frozen grapevine leaves of regenerated transgenic events as described (Lodhi et al. 1994) and quantified with a Nanodrop microvolume spectrophotometer (Thermo-Fisher). DNA samples (10 μg) from control empty vector and transgenic plants were digested with either *Bam*HI or *Hin*dIII restriction enzymes (New England Biolabs), electrophoresed in 1% agarose gels in 1 × Tris–borate–EDTA, and subjected to Southern blot analysis (Southern 1975).

Agarose gels were blotted onto positively charged Amersham Hybond-N+ nylon membrane (GE Healthcare Life Sciences, USA) using the capillary transfer (Thermoscientific, USA) and the membrane was UV-crosslinked (SpectroLinker XL-1500, Spectroline, Westbury NY). PCR amplified *nptII* and *cas9* coding sequences were gel purified and randomly labelled with [α-^32^P]dCTP, 3000 Ci/mmol (Perkin Elmer, www.perkinelmer.com) and used as probes. Hybridization was performed at 65°C for 16–20 h. Post-hybridization washes were performed as follows: The hybridization solution was discarded, and the blots were washed at 65°C twice with 2X SSC/0.5% SDS and four times with 0.2X SSC/0.5% SDS. The radioactivity signals were scanned using a Personal Molecular Imager™ system (www.bio-rad.com).

### Detection of CRISPR/Cas9-induced genome editing of target genes

#### a) Targeted amplicon sequencing

Characterization of genome editing events of target genes was by done by Amplicon-Ez targeted amplicon sequencing (Genewiz, South Plainfield, NJ). A 300 bp amplicon comprising the gRNA target region was PCR amplified using KAPA HiFi HotStart ReadyMix. Partial Illumina adapters were fused to the 5′ end of the gene specific PCR primers (PCR Primer sequences are listed in Suppl. Table 7). The PCR products were gel purified using Zymoclean gel DNA recovery kit and subjected to polyacrylamide gel electrophoresis-based genotyping (Zhu et al. 2014) and sequenced (Genewiz). The targeted amplicon sequencing was repeated in vegetatively propagated transgenic plant clones.

#### b) Genomic library sequencing and analysis

Genomic libraries were prepared using 200 ng of total DNA as input according to the instructions provided by TruSeq Nano DNA Sample Preparation kit for 550 bp insert size (Illumina^®^). Six DNA libraries (two empty vector, two TAS4b, and two MYBA7 transgenic plants) were constructed with eight bp dual-indexed adapters. The quality of each library was assessed using an Agilent High Sensitivity DNA chip on an Agilent 2100 Bioanalyzer. Equi-molar concentrations of libraries were pooled and sequenced on Illumina NovaSeq SP lane at the University of California, Los Angeles Genomics Core Facility. The reads obtained were analyzed using Magic-Blast (Boratyn et al. 2019) for targeted and off-target editing, SPAdes (Nurk et al. 2013) for de novo assembly of T-DNA integration scaffolds, and bowtie (Langmead et al. 2009) for non-T-DNA integration.

## Results

### Genome editing by CRISPR/Cas9 of *Vvi*-*TAS4b* and *Vvi*-*MYBA7* genes

**Fig. 1:**
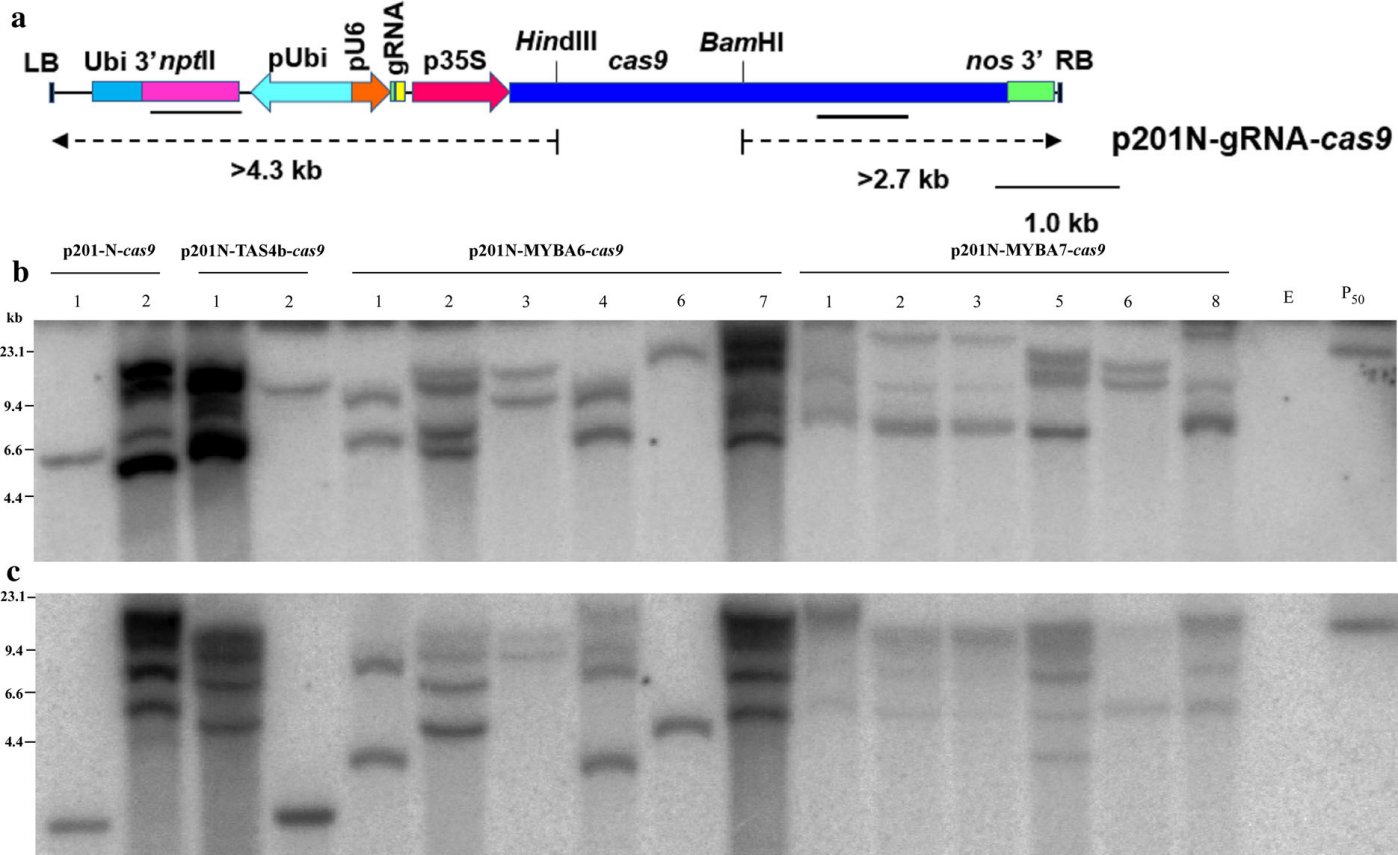
Southern blot analysis of grape plants transformed with CRISPR vectors p201-gRNA-*cas9*. **a** The T-DNA of the binary vector p201N-gRNA-cas9. RB: T-DNA right border. p35S: Cauliflower mosaic virus 35S promoter. *cas9*: CRISPR associated protein9, human codon optimized. *nos 3*′: polyadenylation signal of the nopaline synthase gene. pU6: *Medicago truncatula* U6.6 promoter. gRNA: guide RNA. pUbi: maize ubiquitin promoter. *nptII*: neomycin phosphotransferase gene. Ubi 3′: ubiquitin 3′ polyadenylation signal. LB: T-DNA left border. Probes used (*nptII* and *cas9*) have been marked in bold lines. The junction fragment sizes > 4.3 kb and > 2.7 kb have been marked in a dashed arrow. **b**, **c** Southern blot analysis of grape plants transformed and regenerated with p201N-gRNA-*cas9* probed with *nptII* and *cas9*, respectively; P_50_ plasmid p201N-MYBA6-cas9 was used as hybridization positive control. Total DNA was extracted from 16 plants (two vector alone, two p201N-TAS4b-cas9, six p201N-MYBA6-*cas9*, and six p201N-MYBA7-*cas9*) rooted under kanamycin selection. **b** DNA (10 μg) digested with *Hin*dIII probed with *nptII*. T-DNA junction restriction fragments > 4.3 kb for transgenic plants are expected. **c** DNA (10 μg) digested with *Bam*HI probed with *cas9*. T-DNA junction restriction fragments > 2.7 kb for transgenic plants are expected. (Color figure online)

The synthetic guide RNA (sgRNA) sequences of interest (Table 1), including potential off target loci, were identified by manual inspection and computationally (Bae et al. 2014; Liu et al. 2017). Synthetic oligonucleotides were designed to overlap with the U6 promoter sequence in p201N-cas9 as described (Jacobs et al. 2015; Jacobs and Martin 2016) to yield p201N-gRNA-cas9 using the NEBuilder HiFi DNA Assembly Cloning Kit (New England Biolabs). The p201N-cas9 vector harbors the neomycin phosphotransferaseII (*nptII*) gene as the plant selectable marker (Fig. 1a). We used *Agrobacterium* strain EHA105, a T-DNA deletion derivative of hyper-virulent Ti plasmid pTiBo542 (Hood et al. 1993), carrying binary p201N-gRNA-cas9 vectors (Jacobs et al. 2015) targeting the *MIR828*, *TAS4a/b*, and *MYBA6* and *MYBA7* loci to transform embryogenic callus derived from anthers of the commercially relevant grapevine rootstock 101-14 (Tricoli et al. 2014). 101-14 produces requisite marker anthocyanins for phenotyping of transgene events. Grape transformation with p201N-gRNA-cas9 constructs listed in Table I yielded two kanamycin-resistant *TAS4b* plants (*TAS4b*-*1*, *TAS4b*-*2*), six *MYBA6* plants (*MYBA6*-1, -2, -3, -4, -6 and -7) and six *MYBA7* plants (*MYBA7*-1, -2, -3, -5, -6 and -8). We also obtained two empty vector (with no gRNA cassette) transgenic plants (cas9-1, cas9-2).

T-DNA integrations in kanamycin-resistant regenerated plants (expressing the *nptII* gene) were characterized by genomic Southern blots hybridized with *nptII* and *cas9* probes. Digestion of genomic DNA with *Hin*dIII and hybridization with the *nptII* probe is predicted to yield junction fragments for integration events that include the left T-DNA border outside of the selectable marker larger than 4.3 kb. The integration of the *cas9* sequence mapping inside the right T-DNA border was assayed by digestion of genomic DNA with *Bam*HI enzyme. Junction fragments larger than 2.7 kb are expected to hybridize when T-DNA integration events include the right T-DNA border. Southern blot analysis showed that all of the transgenic plants had at least one integrated copy of the T-DNA harboring both the *nptII* (Fig. 1b) and *cas9* genes (Fig. 1c). Junction fragment analysis revealed that MYBA7-2 and -3 events were likely clones that regenerated from the same transformation event (Fig. 1b, c).

**Fig. 2:**
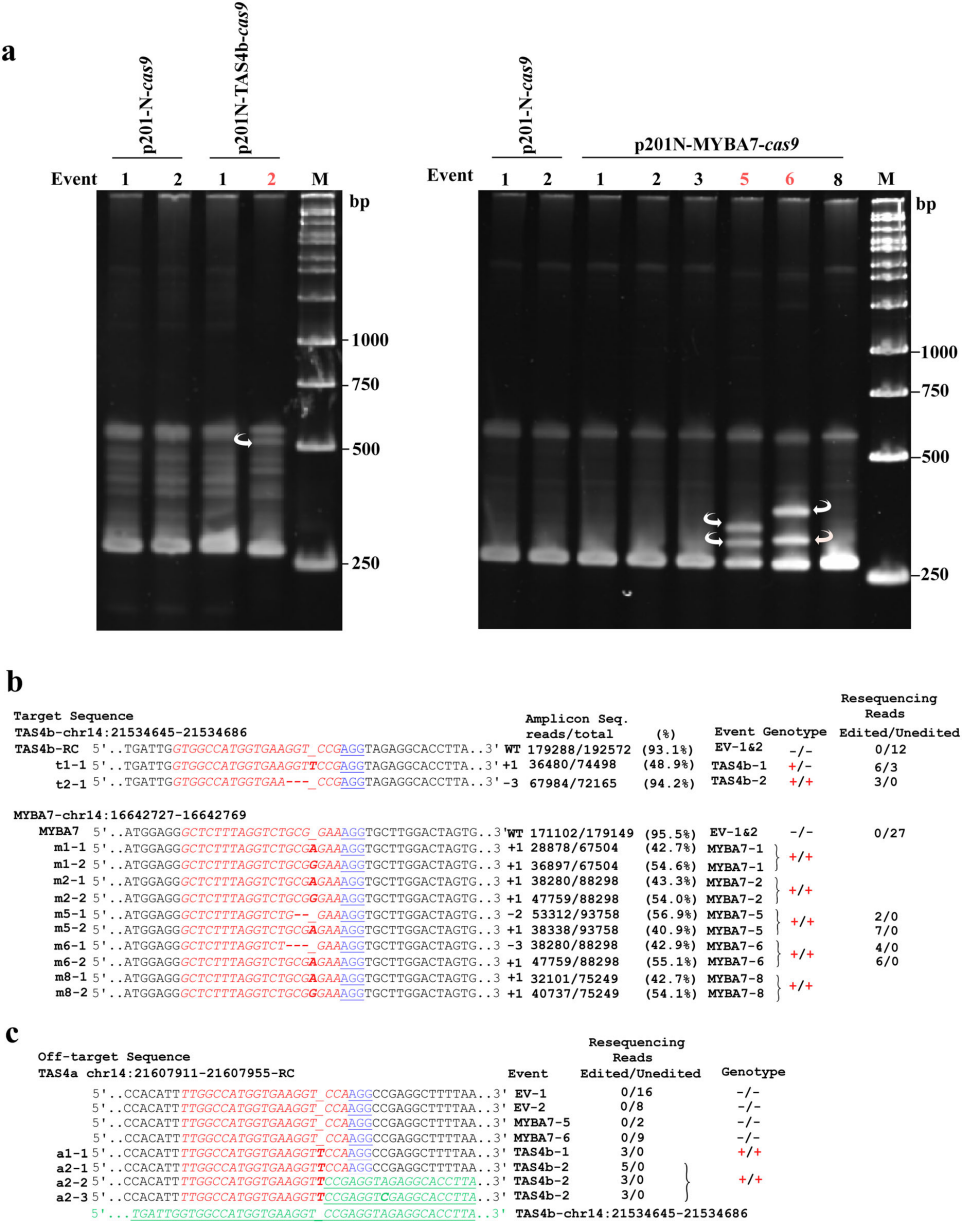
Evidence for genome editing of *TAS4b* and *MYBA7* in transgenic grapevine events. **a** Polyacrylamide gel electrophoresis heteroduplex amplicon assay (Zhu et al. 2014) showing candidate editing events in stably transformed grapevine regenerants (white arrows). p201-N-cas9: empty vector control regenerants. **b** Validation by deep sequencing of amplicons and genomic sequencing of transgenics for independent grapevine *TAS4b* and *MYBA7* CRISPR editing events (far right column) resulting in one nt insertions (bold italicized red), two nt or three nt deletions (bold dashes) at expected positions three nt upstream of the Proximal Adjacent Motif (PAM; underlined blue) in target guide sequences (italicized red). The *MYBA7* insertion events result in a stop codon five amino acids downstream from frame shifts. ***TAS4b*-*RC***: Reverse complement sequence of guide RNA; ***EV*-*1&2***: Empty vector transgenic plants cas9-1 and cas9-2; ***t1*-*1***: TAS4b-1edited event; ***t2*-*1***: TAS4b-2 edited event; ***m1*-*1*and*m1*-*2***: MYBA7-1 edited event 1 and 2; ***m2*-*1*and*m2*-*2***: MYBA7-2 edited event 1 and 2; ***m5*-*1*and*m5*-*2***: MYBA7-5 edited event 1 and 2; ***m6*-*1*and*m6*-*2***: MYBA7-6 edited event 1 and 2; ***m8*-*1*and*m8*-*2***: MYBA7-8 edited event 1 and 2. **c** Detection of off-target effect on TAS4a locus by genomic sequencing of TAS4b transgenic plants. TAS4a locus is unedited in empty vector transgenic plants (EV-1 and EV2) and in MYBA7 transgenic plants (MYBA7-5 and MYBA7-6). ***a1*-*1***: TAS4a locus edited TAS4b-1 plant; ***a2*-*1***, ***a2*-*2 and a2*-*3***: Three different editing events of TAS4a locus in TAS4b-2 plant. Color scheme: one nt insertion (bold italicized red); target sequences (italicized red); TAS4a/b recombinant sequences (italicized green underlined). **b**, **c -*/*-**: unedited genotype; **+/-**: mono-allelic editing; **+*/*+**: bi-allelic editing. (Color figure online)

To initially identify candidate genome-edited events in transgenic plants, facile polyacrylamide gel electrophoresis-based genotyping (Zhu et al. 2014) was performed. PAGE heteroduplex analysis is based on the rationale that DNA heteroduplexes with bulges migrate in gels at a slower rate than homoduplexes. PCR amplification of target sequences results in a mixture of amplicons from template variants that can include edited allele(s). Denaturation and renaturation of PCR products result in homoduplexes if there is no template complexity or heteroduplexes with different gel migration rates. Based on the differences in migration of bands compared to vector alone regenerant control plants, Fig. 2a shows evidence for one candidate editing event for TAS4b (lane event number 2), and at least two editing events for MYBA7 (lane event numbers 5 and 6).

A 300 bp gRNA target region was amplified by PCR from genomic DNA template extracted from each transgenic line and vector-alone control line using primers containing partial Illumina adapter sequences incorporated in the 5′ end of the primers. The PCR products were gel-purified and assessed by targeted deep sequencing (Amplicon-EZ, Genewiz; South Plainfield, NJ) (Fig. 2b). The identified two bp editing deletion of MYBA7-5 (m5-1) changed the reading frame of the polypeptide while the in-frame three bp deletions for characterized MYBA7 line #6 (m6-1) at the target site are predicted to delete residue 8^Arg^ from the polypeptide, whereas the bi-allelic A/G single bp insertion dual mutations in MYBA7 lines #1, 2, 5, 6, and 8 are predicted to cause translation termination after residue 13^Asp^. We also observed evidence of mono- and bi-allelic cas9-mediated events: a single bp insertion in TAS4b line #1 (t1-1) and 3 bp deletion in TAS4b #2 (t2-1). The editing events characterized by amplicon deep sequencing were independently confirmed by whole genome resequencing of libraries made from genomic DNAs extracted from two empty vector transgenic plants (cas9-1 and -2), two TAS4b edited plants (TAS4b-1 and -2), and two MYBA7 edited plants (MYBA7-5 and -6). We confirmed the accuracy of results from two rounds of amplicon deep sequencing by mapping whole genome resequencing datasets using Magic-Blast (Boratyn et al. 2019) (Suppl. Table 1). Empty vector-transformed transgenic plants had intact unedited target sites, confirmed by 12 TAS4b unedited reads and 27 MYBA7 reads, respectively. The editing event in TAS4b-1 (t1-1) was confirmed to be heterozygous (**+/-**) with six edited reads and 3 unedited reads found in whole genome resequencing libraries (Fig. 2b; Suppl. Table 1) while the three bp deletion of TAS4b-2 was confirmed to be bi-allelic (+/+) with 3 edited reads and zero unedited reads (Fig. 2b; Suppl. Table 1). Resequencing of the MYBA7-5 transgenic line was confirmed to be bi-allelic with a two bp deletion event m5-1 (2 reads) and a single bp insertion of “A” for m5-2 (7 reads). Similarly, MYBA7-6 was confirmed to be bi-allelic with a three bp deletion m6-1 (4 reads) and a single bp insertion of “A” for m6-2 (6 reads) (Fig. 2b; Suppl. Table 1). We did not detect any unedited wild type reads in MYBA7-5 and MYBA7-6. Presumably because of genetic redundancy of *TAS4* (three loci; a, b, c) and *MYBA7* (two paralogous loci, *MYBA5* and *MYBA6*), anthocyanin phenotypes did not obviously manifest in the regenerated transgenic lines.

### Detection of an off-target effect from TAS4b- but not MYBA7-gRNAs

The predicted off-targets of TAS4b and MYBA7 gRNA were mined using the online tool CRISPR-P 2.0 (Liu et al. 2017) based on seed microhomology (Bae et al. 2014) and listed in Suppl. Table 2. Interestingly, we found polymorphism of the off-target sequence in 101-14 rootstocks when we analyzed resequencing data available in the public domain (Liang et al. 2019) (SRR5891889_1.fastq and SRR5891889_2.fastq), in comparison to the reference genome. The 101-14 polymorphic sequence of the off-target sequence is listed in Suppl. Table 2. We checked for off-target editing in the genomic resequencing data of two empty vector transgenic plants (cas9-1 and -2), two TAS4b edited plants (TAS4b-1 and -2) and two MYBA7 edited plants (MYBA7-5 and -6). The top off-target hit for *TAS4b* was its closest homolog *TAS4a* with two bp mismatch between the gRNA and the off-target sequence (Suppl. Table 2). The unedited off-target sequence was observed as wild type in empty vector (EV-1 and EV-2) and MYBA7 transgenic plants (MYBA7-5 and -6) (Fig. 2c; Suppl. Table 3). In the TAS4b-1 edited plant, we found the bi-allelic editing of off-target *TAS4a* sequence with a single bp insertion (a1-1) (Fig. 2c; Suppl. Table 3). In TAS4b-2 three bp bi-allelic deletion plant, we found two editing events in the *TAS4a* off-target locus. A single bp insertion at *TAS4a*, similar to the off-target event in TAS4b-1 plant was observed in TAS4b-2 plant (a2-1) (Fig. 2c; Suppl. Table 3). The second off target single base insertion editing event was coupled with apparent homologous recombinations between *TAS4a* and *TAS4b*. The most parsimonious account of the reads suggest Cas9-mediated dsDNA breaks in *TAS4a* resulted in independent single bp insertions three bp upstream of the PAM sequence followed by strand exchange with either intra- or inter-strand versions of the *TAS4b* locus (a2-2 and a2-3) (Fig. 2c; Suppl, Table 3). The evidence for independent recombination events are multiple reads in the same TAS4b-2 plant library of a C/A polymorphism found two nt downstream of the PAM in *TAS4a*. Consistent with independent homologous recombination events associated with high Cas9 guide activity is that no reciprocal exchange events with *TAS4b* (i.e. the complementary product of a single bi-molecular recombination reaction) were detected in the TAS4b-2 resequencing library. No off-target effects were observed in MYBA7 edited plants MYBA7-5 or -6, including for candidate off target MYBA6, the closest homolog of MYBA7 (Suppl. Table 4).

### Characterization of T-DNA integration loci in transgenic plants

T-DNA integration is often incomplete with truncated T-DNA transfer (Spielmann and Simpson 1986; Yin and Wang 2000) and non-T-DNA vector backbone portions integrated randomly in the genome (Ramanathan and Veluthambi 1995). To check for non-T-DNA portion integrations we performed bowtie (Langmead et al. 2009) mapping of the resequencing libraries against the p201-N-cas9 vector sequence. The output revealed long T-DNA integrations past the left border in all transgenic plants assayed (Suppl. Table 5). We observed concordant increases in non-T-DNA integrations with increased copy numbers of T-DNA integrated evidenced by intense Southern hybridization signals in certain events (Fig. 1b; p201-N-cas9 empty vector event 2; TAS4b event 1). Next, we mapped the T-DNA integration loci by finding chimaeric 101 bp resequencing reads mapping to the vector yet having perfect homology at read end overhangs to the grapevine reference genome using Magic-Blast (Boratyn et al. 2019). We independently assessed the T-DNA integration event structures using de novo assembler SPAdes (Nurk et al. 2013). Both tools mapped at least one edge of an integration with evidence of chromosomal grape sequences chimaeric with a T-DNA left and or right border sequence. Specifically, we identified integration of the empty vector Cas9-1 event to chr8:2688372-2688357, MYBA7-6 event to chr15: 12878391–12878411 (Suppl. Table 6; Suppl.docx 1; Suppl.docx 2). Magic-Blast mapped TAS4b- 1 to chr12: 14666332–14666303 and TAS4b-2 to chr4: 10021502–10021519. We were unsuccessful by these methods to map the integration sites of either cas9-2 or MYBA7-5, most likely due to the complex, apparently higher copy number T-DNA integrations of these lines (Fig. 1b). A further technical issue for these samples was the read depths of these two libraries were relatively shallow, ~ 78% and 37% respectively versus the average depths of the libraries successful to identify multiple chimaeric reads that established T-DNA integration loci (Suppl. Table 6). The consequence was the number of de novo-assembled contigs were higher for these two libraries, with corresponding shorter contig lengths.

## Discussion

Many labs have demonstrated high efficiency (~ 80% including bi-allelic/homozygous mutations in primary transformants) of plant genome editing by CRISPR/Cas9 synthetic guide technologies, reviewed in Belhaj et al. (2013), Bisht et al. (2019), Mushtaq et al. (2019) and Raitskin and Patron (2016). CRISPR induces DNA double-strand breaks at specific genome sites that have a high propensity to result in multiple independent site-directed mutations through error-prone non-homologous end joining. Recent results including deployment of CRISPR-Cas12a (Cpf1) from *Prevotella* and *Francisella* show the method is practical for engineering resistance in Duncan grapefruit to citrus canker (Jia et al. 2015, 2019) caused by the bacterium *Xanthomonas axonopodis* by modifying the PthA4 pathogenicity effector binding elements in the promoter of *Cs*-*Lateral Organ Boundaries1* susceptibility gene. Five reports have documented the efficacy of creating events by CRISPR/Cas9 *Agrobacterium*-mediated transformation/regeneration in grapevine: targeting *Vvi*-*Phytoene Desaturase* in cv Muscat (Nakajima et al. 2017) and cv Chardonnay and 41B (Ren et al. 2019) as visible marker for bi-allele knockout efficiency, the *Vvi*-*WRKY52* gene (~ 70% biallelic events) in cv Thompson seedless for resistance to noble rot caused by *Botrytis cinerea* (Wang et al. 2018), and the *Vvi*-*L*-*idonate dehydrogenase/IdnDH* gene for tartaric acid biosynthesis in cv Chardonnay (Osakabe et al. 2018; Ren et al. 2016). In each case, and especially the latter, a preponderance of events were one bp insertions and three bp deletions like we observed for predominantly bi-allelic *Vvi*-*TAS4ab* and *Vvi*-*MYBA7* regenerants (Fig. 2). Another report documented grapevine cv Chardonnay protoplasts as suitable starting material for CRISPR/Cas9 editing at an estimated rate of 0.1% indels generated in a candidate powdery mildew susceptibility gene *MLO*-*7* (Malnoy et al. 2016). Because transgenic cells expressing CRISPR/Cas9 constructs are subject to mutations arising independently as a function of Cas9 activity, the limitations of low transformation efficiency in grapevine appear to have been overcome by high Cas9 performance in our transgene events (Fig. 2b, c). Nakajima et al. (2017) observed from the visible phenotype of bi-allelic CRISPR-induced mutations in the *Phytoene Desaturase1* gene of grapevine a correlation between leaf age and mutation rates.

Target specificity is an important issue for all genome editing technologies, including CRISPR/Cas9. Off-targets have been addressed with experimental evidence for some off-target activity in rice, barley and *Brassica oleracea* (Lawrenson et al. 2015) but not in *N. benthamiana* [reviewed in Raitskin and Patron (2016)] or in the documented cases of grapevine CRISPR (Nakajima et al. 2017; Osakabe et al. 2018; Ren et al. 2016, 2019; Wang et al. 2018). We observed fortuitous off-target mutations of *TAS4b* homolog *TAS4a*, wherein the off-target sequence had just two bp mismatches between the *TAS4b* gRNA and the off target. We observed a single bp insertion in TAS4b-1 and -2 plants and novel homologous recombinations of *TAS4a*-*TAS4b* loci in the TAS4b-2 plant remarkably with apparent breakpoints at the Cas9-induced dsDNA break (Fig. 2c). Several recent works have shown CRISPR-mediated targeted recombination in Drosophila (Brunner et al. 2019), yeast (Sadhu et al. 2016), and tomato (Filler-Hayut et al. 2017). Studies have shown that design of gRNAs with at least three mismatches from other genomic regions can alleviate off-target editing (Young et al. 2019). Polymorphism of *TAS4a* locus in 101-14 rootstock genotype appears to reduce the number of mismatches to two residues compared to the Pinot Noir reference genome, and thus likely made the locus more prone to off target activity by TAS4b gRNA. Thus, our findings underscore the importance of exploring for off-target effects in vivo especially when rootstocks and/or cultivars other than the reference genome are subject matter for genome editing experiments. It is worth noting that the off-target effect of *TAS4b* gRNA on the *TAS4a* locus was fortuitous and to our advantage wherein we successfully edited two homologous loci of interest with a single guide RNA.

Due to genetic redundancy for *MYBA5/6/7* and *TAS4a/b/c* loci, it remains to be determined whether the events characterized here will have visible phenotypes impacting anthocyanin pigmentation and/or PD resistance/tolerance. Future experiments can employ multiple guide constructs (Jacobs and Martin 2016) to target all *MYBA* and *TAS4* family members, and to target the sole *Vvi*-*MIR828* locus at locations upstream or downstream of the mature miR828 in the hairpin structure to generate leaky dominant-negative alleles predicted to alter DICER processing efficiency. Our initial test construct for *Vvi*-*MIR828* aimed to create null alleles by targeting the mature miR828 duplex per se but we failed to recover any regenerants, consistent with a speculated essential function of *MIR828*.

There is now scope with these novel edited materials to assess molecular phenotypes of deranged gene expression, pathogen resistance/tolerance, vector feeding preferences (Zeilinger et al. 2018), and GRBV systemic movement by agroinoculations (Yepes et al. 2018) on these genome-edited anthocyanin host effector grapevine lines. However, dissolution of the transgenic state or conversion to homozygosity of mutated haplotypes (like TAS4b-2-a2; Fig. 2c) and non-mutated wild type alleles (like TAS4b-1) to obtain desired homozygous plants by back-crossing and genetic segregation of sexually reproduced individual progeny is a lengthy route. This is because of the difficulty to obtain successful crosses of grapevine with greenhouse material, and several years of time elapsed before regenerated field-grown juvenile transgenic plants will flower. Further work on fundamental processes of plant interactions with GRBV and XF can leverage translational science from model organisms to crops, with potential for broad impacts on agriculture, development of sustainable nutrient management tools, and understanding the mechanisms of pathogen resistance/tolerance and pleiotropic disease states.

## Conclusions

CRISPR-cas9 technology has been successfully used to knock-down several protein coding genes in several plant species. Although successful editing of non-coding RNAs has been demonstrated in animals [reviewed in Basak and Nithin (2015)], the only report on editing of a non-coding RNA is in tomato (Li et al. 2018). We demonstrate successful gene editing of a non-coding regulatory RNA *TAS4a/b* in grape cultivar 101-14, an anthocyanin producing rootstock. We further demonstrate fortuitous off-target effects of TAS4b guide RNA on *TAS4a* locus resulting in a chimeric TAS4a-b locus subject to homologous recombination events associated with off-target editing. Future studies are now possible to test the roles of *Vvi*-*MYBA7* and *Vvi*-*TAS4a/b/c* in tissue-specific anthocyanin expression and the genes’ roles in microbe and virus disease etiologies and possibly feeding preferences of arthropod vectors (Zeilinger et al. 2018).

## Electronic supplementary material

Below is the link to the electronic supplementary material.

Supplementary material 1 (RTF 207 kb)

Supplementary material 2 (RTF 288 kb)

Supplementary material 3 (XLSX 24 kb)

Supplementary material 4 (XLSX 11 kb)

Supplementary material 5 (XLSX 66 kb)

Supplementary material 6 (XLSX 51 kb)

Supplementary material 7 (XLSX 1501 kb)

Supplementary material 8 (XLSX 20 kb)

Supplementary material 9 (XLSX 10 kb)

## Data Availability

The sequencing runs were submitted as raw fastq files to NCBI Sequence Read Archive with Bioproject accession PRJNA602781. Authors will freely share transgenic events and other materials described in this article via a Materials Transfer Agreement promulgated by the Texas Tech University Office of Technology Commercialization (TTU-OTC). All data needed to evaluate the conclusions in the paper are present in the paper. Additional data related to this paper may be requested from the authors.

## References

[CR1] Almeida RPP, Nunney L (2015). How do plant diseases caused by *Xylella fastidiosa* emerge?. Plant Dis.

[CR2] Bae S, Kweon J, Kim HS, Kim J-S (2014). Microhomology-based choice of Cas9 nuclease target sites. Nat Methods.

[CR3] Bahder BW, Zalom FG, Jayanth M, Sudarshana MR (2016). Phylogeny of geminivirus coat protein sequences and digital PCR aid in identifying *Spissistilus festinus* as a vector of grapevine red blotch-associated virus. Phytopathology.

[CR4] Barbehenn RV, Constabel CP (2011). Tannins in plant-herbivore interactions. Phytochemistry.

[CR5] Basak J, Nithin C (2015). Targeting non-coding RNAs in plants with the CRISPR-Cas technology is a challenge yet worth accepting. Front Plant Sci.

[CR6] Belhaj K, Chaparro-Garcia A, Kamoun S, Nekrasov V (2013). Plant genome editing made easy: targeted mutagenesis in model and crop plants using the CRISPR/Cas system. Plant Methods.

[CR7] Bisht DS, Bhatia V, Bhattacharya R (2019). Improving plant-resistance to insect-pests and pathogens: the new opportunities through targeted genome editing. Semin Cell Dev Biol.

[CR8] Boratyn GM, Thierry-Mieg J, Thierry-Mieg D, Busby B, Madden TL (2019). Magic-BLAST, an accurate RNA-seq aligner for long and short reads. BMC Bioinform.

[CR9] Brunner E (2019). CRISPR-induced double-strand breaks trigger recombination between homologous chromosome arms. Life Sci Alliance.

[CR10] Calvi BL (2011) Effects of red-leaf disease on Cabernet Sauvignon at the Oakville experimental vineyard and mitigation by harvest delay and crop adjustment. M.Sc. thesis. University of California, Davis

[CR11] Cantos E, Espín JC, Tomás-Barberán FA (2002). Varietal differences among the polyphenol profiles of seven table grape cultivars studied by LC–DAD–MS–MS. J Agric Food Chem.

[CR12] Cong L (2013). Multiplex genome engineering using CRISPR/Cas systems. Science.

[CR13] De La Fuente L, Parker JK, Oliver JE, Granger S, Brannen PM, van Santen E, Cobine PA (2013). The bacterial pathogen *Xylella fastidiosa* affects the leaf ionome of plant hosts during infection. PLoS ONE.

[CR14] Filler-Hayut S, Melamed Bessudo C, Levy AA (2017). Targeted recombination between homologous chromosomes for precise breeding in tomato. Nat Commun.

[CR15] Hood EE, Gelvin SB, Melchers LS, Hoekema A (1993). New *Agrobacterium* helper plasmids for gene transfer to plants. Transgenic Res.

[CR16] Hsieh LC (2009). Uncovering small RNA-mediated responses to phosphate deficiency in Arabidopsis by deep sequencing. Plant Physiol.

[CR17] Jacobs TB, Martin GB (2016). High-throughput CRISPR vector construction and characterization of DNA modifications by generation of tomato hairy roots. J Vis Exp.

[CR18] Jacobs T, LaFayette P, Schmitz R, Parrott W (2015). Targeted genome modifications in soybean with CRISPR/Cas9. BMC Biotechnol.

[CR19] Jia H, Orbović V, Jones JB, Wang N (2015). Modification of the PthA4 effector binding elements in Type I CsLOB1 promoter using Cas9/sgRNA to produce transgenic Duncan grapefruit alleviating XccΔpthA4:dCsLOB1.3 infection. Plant Biotechnol J.

[CR20] Jia H, Orbović V, Wang N (2019). CRISPR-LbCas12a-mediated modification of citrus. Plant Biotechnol J.

[CR21] Jinek M, Chylinski K, Fonfara I, Hauer M, Doudna JA, Charpentier E (2012). A programmable dual-RNA-guided DNA endonuclease in adaptive bacterial immunity. Science.

[CR22] Johnson ET, Berhow MA, Dowd PF (2010). Constitutive expression of the maize genes B1 and C1 in transgenic Hi II maize results in differential tissue pigmentation and generates resistance to *Helicoverpa zea*. J Agric Food Chem.

[CR23] Krivanek AF, Walker MA (2004). Vitis resistance to Pierce’s disease is characterized by differential *Xylella fastidiosa* population in stems and leaves. Phytopathology.

[CR24] Krugner R, Hagler JR, Groves RL, Sisterson MS, Morse JG, Johnson MW (2012). Plant water stress effects on the net dispersal rate of the insect vector *Homalodisca vitripennis* (Hemiptera: Cicadellidae) and movement of its egg parasitoid, *Gonatocerus ashmeadi* (Hymenoptera: Mymaridae). Environ Entomol.

[CR25] Kyrkou I, Pusa T, Ellegaard-Jensen L, Sagot M-F, Hansen LH (2018). Pierce’s Disease of grapevines: a review of control strategies and an outline of an epidemiological model. Front Microbiol.

[CR26] Langmead B, Trapnell C, Pop M, Salzberg S (2009). Ultrafast and memory-efficient alignment of short DNA sequences to the human genome. Genome Biol.

[CR27] Lawrenson T (2015). Induction of targeted, heritable mutations in barley and *Brassica oleracea* using RNA-guided Cas9 nuclease. Genome Biol.

[CR28] Li R, Fu D, Zhu B, Luo Y, Zhu H (2018). CRISPR/Cas9-mediated mutagenesis of lncRNA1459 alters tomato fruit ripening. Plant J.

[CR29] Liang Z (2019). Whole-genome resequencing of 472 Vitis accessions for grapevine diversity and demographic history analyses. Nat Commun.

[CR30] Lin SI, Chiang SF, Lin WY, Chen JW, Tseng CY, Wu PC, Chiou TJ (2008). Regulatory network of microRNA399 and PHO2 by systemic signaling. Plant Physiol.

[CR31] Lin W-Y, Lin Y-Y, Chiang S-F, Syu C, Hsieh L-C, Chiou T-J (2018). Evolution of microRNA827 targeting in the plant kingdom. New Phytol.

[CR32] Liu H, Ding Y, Zhou Y, Jin W, Xie K, Chen L-L (2017). CRISPR-P 2.0: an improved CRISPR-Cas9 tool for genome editing in plants. Mol Plant.

[CR33] Lodhi MA, Ye G-N, Weeden NF, Reisch BI (1994). A simple and efficient method for DNA extraction from grapevine cultivars and *Vitis* species. Plant Mol Biol Rep.

[CR34] Luo Q-J, Mittal A, Jia F, Rock CD (2012). An autoregulatory feedback loop involving *PAP1* and *TAS4* in response to sugars in Arabidopsis. Plant Mol Biol.

[CR35] Maddox C, Laur L, Tian L (2010). Antibacterial activity of phenolic compounds against the phytopathogen *Xylella fastidiosa*. Curr Microbiol.

[CR36] Makoi J, Belane AK, Chimphango SBM, Dakora FD (2010). Seed flavonoids and anthocyanins as markers of enhanced plant defence in nodulated cowpea (*Vigna unguiculata* L. Walp.). Field Crops Res.

[CR37] Malnoy M (2016). DNA-free genetically edited grapevine and apple protoplast using CRISPR/Cas9 ribonucleoproteins. Front Plant Sci.

[CR38] Meyer MM, Kirkpatrick BC (2011). Exogenous applications of abscisic acid increase curing of Pierce’s disease-affected grapevines growing in pots. Plant Dis.

[CR39] Mushtaq M (2019). Harnessing genome editing techniques to engineer disease resistance in plants. Front Plant Sci.

[CR40] Myles S (2011). Genetic structure and domestication history of the grape. Proc Natl Acad Sci U S A.

[CR41] Nakajima I (2017). CRISPR/Cas9-mediated targeted mutagenesis in grape. PLoS ONE.

[CR42] Nurk S (2013). Assembling single-cell genomes and mini-metagenomes from chimeric MDA products. J Comput Biol.

[CR43] Oberhoster A et al (2016) Impact of Red Blotch Disease on grape and wine composition. Workshop on Recent Advances in Viticulture and Enology, UC Davis, Dec 9, 2016. http://ucanr.edu/repository/fileaccess.cfm?article=162938&p=YQCEFO. Accessed 11 Apr 2020

[CR44] Osakabe Y (2018). CRISPR–Cas9-mediated genome editing in apple and grapevine. Nat Protocols.

[CR45] Raitskin O, Patron NJ (2016). Multi-gene engineering in plants with RNA-guided Cas9 nuclease. Curr Opin Biotechnol.

[CR46] Rajagopalan R, Vaucheret H, Trejo J, Bartel DP (2006). A diverse and evolutionarily fluid set of microRNAs in *Arabidopsis thaliana*. Genes Dev.

[CR47] Raju BC, Goheen AC (1981). Relative sensitivity of selected grapevine cultivars to Pierce’s disease bacterial inoculations. Am J Enol Vitic.

[CR48] Ramanathan V, Veluthambi K (1995). Transfer of non-T-DNA portions of the *Agrobacterium tumefaciens* Ti plasmid pTiA6 from the left terminus of T_L_-DNA. Plant Mol Biol.

[CR49] Ren C, Liu X, Zhang Z, Wang Y, Duan W, Li S, Liang Z (2016). CRISPR/Cas9-mediated efficient targeted mutagenesis in Chardonnay (*Vitis vinifera* L.). Sci Rep.

[CR50] Ren F, Ren C, Zhang Z, Duan W, Lecourieux D, Li S, Liang Z (2019). Efficiency optimization of CRISPR/Cas9-mediated targeted mutagenesis in grape. Front Plant Sci.

[CR51] Rock CD (2013). *Trans*-*acting small interfering RNA4*: key to nutraceutical synthesis in grape development?. Trends Plant Sci.

[CR52] Rwahnih MA, Rowhani A, Golino DA, Islas CM, Preece JE, Sudarshana MR (2015). Detection and genetic diversity of grapevine red blotch-associated virus isolates in table grape accessions in the National Clonal Germplasm Repository in California. Can J Plant Pathol.

[CR53] Sadhu MJ, Bloom JS, Day L, Kruglyak L (2016). CRISPR-directed mitotic recombination enables genetic mapping without crosses. Science.

[CR54] Southern EM (1975). Detection of specific sequences among DNA fragments separated by gel electrophoresis. J Mol Biol.

[CR55] Spielmann A, Simpson RB (1986). T-DNA structure in transgenic tobacco plants with multiple independent integration sites. Mol Gen Genet.

[CR56] Stelinski LL, Rogers ME, Tiwari S (2012). Biochemical basis of organophosphate and carbamate resistance in Asian Citrus Psyllid. J Econ Entomol.

[CR57] Sudarshana MR, Perry KL, Fuchs MF (2015). Grapevine red blotch-associated virus, an emerging threat to the grapevine industry. Phytopathology.

[CR58] Sunitha S, Loyola R, Alcalde JA, Arce-Johnson P, Matus JT, Rock CD (2019). The role of UV-B light on small RNA activity during grapevine berry development. G3 Genes Genomes Genet.

[CR59] Thieme CJ (2015). Endogenous Arabidopsis messenger RNAs transported to distant tissues. Nat Plants.

[CR60] Tricoli D, Chi-Ham C, Prieto H (2014) Development of a grape tissue culture and transformation platform for the California grape research community. In: Proceedings, 2014 Pierce’s disease research symposium California Department of Food and Agriculture, Sacramento, CA, pp 211–219. https://www.cdfa.ca.gov/pdcp/research.html

[CR61] Wallis CM, Chen J (2012). Grapevine phenolic compounds in xylem sap and tissues are significantly altered during infection by *Xylella fastidiosa*. Phytopathology.

[CR62] Wallis CM, Wallingford AK, Chen J (2013). Grapevine rootstock effects on scion sap phenolic levels, resistance to *Xylella fastidiosa* infection, and progression of Pierce’s disease. Front Plant Sci.

[CR63] Wang X (2018). CRISPR/Cas9-mediated efficient targeted mutagenesis in grape in the first generation. Plant Biotechnol J.

[CR64] Wilhelm M, Brodbeck BV, Andersen PC, Kasun GW, Kirkpatrick BC (2011). Analysis of xylem fluid components in almond cultivars differing in resistance to Almond Leaf Scorch disease. Plant Dis.

[CR65] Yamakawa T, Kato S, Ishida K, Kodama T, Minoda Y (1983). Production of anthocyanins by *Vitis* cells in suspension culture. Agric Biol Chem.

[CR66] Yepes LM, Cieniewicz E, Krenz B, McLane H, Thompson JR, Perry KL, Fuchs M (2018). Causative role of grapevine red blotch virus in red blotch disease. Phytopathology.

[CR67] Yin Z, Wang GL (2000). Evidence of multiple complex patterns of T-DNA integration into the rice genome. Theor Appl Genet.

[CR68] Yin Y, Borges G, Sakuta M, Crozier A, Ashihara H (2012). Effect of phosphate deficiency on the content and biosynthesis of anthocyanins and the expression of related genes in suspension-cultured grape (Vitis sp.) cells. Plant Physiol Biochem.

[CR69] Young J (2019). CRISPR-Cas9 editing in maize: systematic evaluation of off-target activity and its relevance in crop improvement. Sci Rep.

[CR70] Zeilinger AR, Del Cid C, Almeida RPP, Krugner R, Daugherty MP (2018). Plant water stress and vector feeding preference mediate transmission efficiency of a plant pathogen. Environ Entomol.

[CR71] Zhu X (2014). An efficient genotyping method for genome-modified animals and human cells generated with CRISPR/Cas9 system. Sci Rep.

