# Supplementary material for: CRISPR/Cas9-mediated targeted mutagenesis of TAS4 and MYBA7 loci in grapevine rootstock 101-14
Source: Transgenic Res. 2020 Apr 23;29(3):355–67. doi: 10.1007/s11248-020-00196-w (PMC7283210; doi:10.1007/s11248-020-00196-w)
Supplement: Supplementary file 1 — Supplementary material 1 (RTF 207 kb) [file 11248_2020_196_MOESM1_ESM.rtf]

Suppl. docx1.  Alignment of SPAdes contig output to p201N-cas9 vector sequence to map the T-DNA integration loci of Cas9-1 transgenic plant

>chr8 chr8:2688322..2688379 (+ strand) 
AAATGCGATTGAAGAGGCGAGATAGCAACAC ACAATACTAATGAAGAAGGTTAACCAA

3nt upstream of RB 
Last four nt of LB and Seq downstream of LB
2x-p35S

>NODE_10593_length_6023_cov_4.641253
                    140         *       160         *       180         *       200         *       220         *       240         *       260        
NODE_10593 : ---------------------------------------------------------------------------------------------------------------------------------- :     -
p201N_cas9 : GGCGTTTTCTTGTCGCGTGTTTTAGTCGCATAAAGTAGAATACTTGCGACTAGAACCGGAGACATTACGCCATGAACAAGAGCGCCGCCGCTGGCCTGCTGGGCTATGCCCGCGTCAGCACCGACGACCA :   260
                                                                                                                                                       
                                                                                                                                                       
                      *       280         *       300         *       320         *       340         *       360         *       380         *        
NODE_10593 : ---------------------------------------------------------------------------------------------------------------------------------- :     -
p201N_cas9 : GGACTTGACCAACCAACGGGCCGAACTGCACGCGGCCGGCTGCACCAAGCTGTTTTCCGAGAAGATCACCGGCACCAGGCGCGACCGCCCGGAGCTGGCCAGGATGCTTGACCACCTACGCCCTGGCGAC :   390
                                                                                                                                                       
                                                                                                                                                       
                    400         *       420         *       440         *       460         *       480         *       500         *       520        
NODE_10593 : ---------------------------------------------------------------------------------------------------------------------------------- :     -
p201N_cas9 : GTTGTGACAGTGACCAGGCTAGACCGCCTGGCCCGCAGCACCCGCGACCTACTGGACATTGCCGAGCGCATCCAGGAGGCCGGCGCGGGCCTGCGTAGCCTGGCAGAGCCGTGGGCCGACACCACCACGC :   520
                                                                                                                                                       
                                                                                                                                                       
                      *       540         *       560         *       580         *       600         *       620         *       640         *        
NODE_10593 : ---------------------------------------------------------------------------------------------------------------------------------- :     -
p201N_cas9 : CGGCCGGCCGCATGGTGTTGACCGTGTTCGCCGGCATTGCCGAGTTCGAGCGTTCCCTAATCATCGACCGCACCCGGAGCGGGCGCGAGGCCGCCAAGGCCCGAGGCGTGAAGTTTGGCCCCCGCCCTAC :   650
                                                                                                                                                       
                                                                                                                                                       
                    660         *       680         *       700         *       720         *       740         *       760         *       780        
NODE_10593 : ---------------------------------------------------------------------------------------------------------------------------------- :     -
p201N_cas9 : CCTCACCCCGGCACAGATCGCGCACGCCCGCGAGCTGATCGACCAGGAAGGCCGCACCGTGAAAGAGGCGGCTGCACTGCTTGGCGTGCATCGCTCGACCCTGTACCGCGCACTTGAGCGCAGCGAGGAA :   780
                                                                                                                                                       
                                                                                                                                                       
                      *       800         *       820         *       840         *       860         *       880         *       900         *        
NODE_10593 : ---------------------------------------------------------------------------------------------------------------------------------- :     -
p201N_cas9 : GTGACGCCCACCGAGGCCAGGCGGCGCGGTGCCTTCCGTGAGGACGCATTGACCGAGGCCGACGCCCTGGCGGCCGCCGAGAATGAACGCCAAGAGGAACAAGCATGAAACCGCACCAGGACGGCCAGGA :   910
                                                                                                                                                       
                                                                                                                                                       
                    920         *       940         *       960         *       980         *      1000         *      1020         *      1040        
NODE_10593 : ---------------------------------------------------------------------------------------------------------------------------------- :     -
p201N_cas9 : CGAACCGTTTTTCATTACCGAAGAGATCGAGGCGGAGATGATCGCGGCCGGGTACGTGTTCGAGCCGCCCGCGCACGTCTCAACCGTGCGGCTGCATGAAATCCTGGCCGGTTTGTCTGATGCCAAGCTG :  1040
                                                                                                                                                       
                                                                                                                                                       
                      *      1060         *      1080         *      1100         *      1120         *      1140         *      1160         *        
NODE_10593 : ---------------------------------------------------------------------------------------------------------------------------------- :     -
p201N_cas9 : GCGGCCTGGCCGGCCAGCTTGGCCGCTGAAGAAACCGAGCGCCGCCGTCTAAAAAGGTGATGTGTATTTGAGTAAAACAGCTTGCGTCATGCGGTCGCTGCGTATATGATGCGATGAGTAAATAAACAAA :  1170
                                                                                                                                                       
                                                                                                                                                       
                   1180         *      1200         *      1220         *      1240         *      1260         *      1280         *      1300        
NODE_10593 : ---------------------------------------------------------------------------------------------------------------------------------- :     -
p201N_cas9 : TACGCAAGGGGAACGCATGAAGGTTATCGCTGTACTTAACCAGAAAGGCGGGTCAGGCAAGACGACCATCGCAACCCATCTAGCCCGCGCCCTGCAACTCGCCGGGGCCGATGTTCTGTTAGTCGATTCC :  1300
                                                                                                                                                       
                                                                                                                                                       
                      *      1320         *      1340         *      1360         *      1380         *      1400         *      1420         *        
NODE_10593 : ---------------------------------------------------------------------------------------------------------------------------------- :     -
p201N_cas9 : GATCCCCAGGGCAGTGCCCGCGATTGGGCGGCCGTGCGGGAAGATCAACCGCTAACCGTTGTCGGCATCGACCGCCCGACGATTGACCGCGACGTGAAGGCCATCGGCCGGCGCGACTTCGTAGTGATCG :  1430
                                                                                                                                                       
                                                                                                                                                       
                   1440         *      1460         *      1480         *      1500         *      1520         *      1540         *      1560        
NODE_10593 : ---------------------------------------------------------------------------------------------------------------------------------- :     -
p201N_cas9 : ACGGAGCGCCCCAGGCGGCGGACTTGGCTGTGTCCGCGATCAAGGCAGCCGACTTCGTGCTGATTCCGGTGCAGCCAAGCCCTTACGACATATGGGCCACCGCCGACCTGGTGGAGCTGGTTAAGCAGCG :  1560
                                                                                                                                                       
                                                                                                                                                       
                      *      1580         *      1600         *      1620         *      1640         *      1660         *      1680         *        
NODE_10593 : ---------------------------------------------------------------------------------------------------------------------------------- :     -
p201N_cas9 : CATTGAGGTCACGGATGGAAGGCTACAAGCGGCCTTTGTCGTGTCGCGGGCGATCAAAGGCACGCGCATCGGCGGTGAGGTTGCCGAGGCGCTGGCCGGGTACGAGCTGCCCATTCTTGAGTCCCGTATC :  1690
                                                                                                                                                       
                                                                                                                                                       
                   1700         *      1720         *      1740         *      1760         *      1780         *      1800         *      1820        
NODE_10593 : ---------------------------------------------------------------------------------------------------------------------------------- :     -
p201N_cas9 : ACGCAGCGCGTGAGCTACCCAGGCACTGCCGCCGCCGGCACAACCGTTCTTGAATCAGAACCCGAGGGCGACGCTGCCCGCGAGGTCCAGGCGCTGGCCGCTGAAATTAAATCAAAACTCATTTGAGTTA :  1820
                                                                                                                                                       
                                                                                                                                                       
                      *      1840         *      1860         *      1880         *      1900         *      1920         *      1940         *        
NODE_10593 : ---------------------------------------------------------------------------------------------------------------------------------- :     -
p201N_cas9 : ATGAGGTAAAGAGAAAATGAGCAAAAGCACAAACACGCTAAGTGCCGGCCGTCCGAGCGCACGCAGCAGCAAGGCTGCAACGTTGGCCAGCCTGGCAGACACGCCAGCCATGAAGCGGGTCAACTTTCAG :  1950
                                                                                                                                                       
                                                                                                                                                       
                   1960         *      1980         *      2000         *      2020         *      2040         *      2060         *      2080        
NODE_10593 : ---------------------------------------------------------------------------------------------------------------------------------- :     -
p201N_cas9 : TTGCCGGCGGAGGATCACACCAAGCTGAAGATGTACGCGGTACGCCAAGGCAAGACCATTACCGAGCTGCTATCTGAATACATCGCGCAGCTACCAGAGTAAATGAGCAAATGAATAAATGAGTAGATGA :  2080
                                                                                                                                                       
                                                                                                                                                       
                      *      2100         *      2120         *      2140         *      2160         *      2180         *      2200         *        
NODE_10593 : ---------------------------------------------------------------------------------------------------------------------------------- :     -
p201N_cas9 : ATTTTAGCGGCTAAAGGAGGCGGCATGGAAAATCAAGAACAACCAGGCACCGACGCCGTGGAATGCCCCATGTGTGGAGGAACGGGCGGTTGGCCAGGCGTAAGCGGCTGGGTTGTCTGCCGGCCCTGCA :  2210
                                                                                                                                                       
                                                                                                                                                       
                   2220         *      2240         *      2260         *      2280         *      2300         *      2320         *      2340        
NODE_10593 : ---------------------------------------------------------------------------------------------------------------------------------- :     -
p201N_cas9 : ATGGCACTGGAACCCCCAAGCCCGAGGAATCGGCGTGACGGTCGCAAACCATCCGGCCCGGTACAAATCGGCGCGGCGCTGGGTGATGACCTGGTGGAGAAGTTGAAGGCCGCGCAGGCCGCCCAGCGGC :  2340
                                                                                                                                                       
                                                                                                                                                       
                      *      2360         *      2380         *      2400         *      2420         *      2440         *      2460         *        
NODE_10593 : ---------------------------------------------------------------------------------------------------------------------------------- :     -
p201N_cas9 : AACGCATCGAGGCAGAAGCACGCCCCGGTGAATCGTGGCAAGCGGCCGCTGATCGAATCCGCAAAGAATCCCGGCAACCGCCGGCAGCCGGTGCGCCGTCGATTAGGAAGCCGCCCAAGGGCGACGAGCA :  2470
                                                                                                                                                       
                                                                                                                                                       
                   2480         *      2500         *      2520         *      2540         *      2560         *      2580         *      2600        
NODE_10593 : ---------------------------------------------------------------------------------------------------------------------------------- :     -
p201N_cas9 : ACCAGATTTTTTCGTTCCGATGCTCTATGACGTGGGCACCCGCGATAGTCGCAGCATCATGGACGTGGCCGTTTTCCGTCTGTCGAAGCGTGACCGACGAGCTGGCGAGGTGATCCGCTACGAGCTTCCA :  2600
                                                                                                                                                       
                                                                                                                                                       
                      *      2620         *      2640         *      2660         *      2680         *      2700         *      2720         *        
NODE_10593 : ---------------------------------------------------------------------------------------------------------------------------------- :     -
p201N_cas9 : GACGGGCACGTAGAGGTTTCCGCAGGGCCGGCCGGCATGGCCAGTGTGTGGGATTACGACCTGGTACTGATGGCGGTTTCCCATCTAACCGAATCCATGAACCGATACCGGGAAGGGAAGGGAGACAAGC :  2730
                                                                                                                                                       
                                                                                                                                                       
                   2740         *      2760         *      2780         *      2800         *      2820         *      2840         *      2860        
NODE_10593 : ---------------------------------------------------------------------------------------------------------------------------------- :     -
p201N_cas9 : CCGGCCGCGTGTTCCGTCCACACGTTGCGGACGTACTCAAGTTCTGCCGGCGAGCCGATGGCGGAAAGCAGAAAGACGACCTGGTAGAAACCTGCATTCGGTTAAACACCACGCACGTTGCCATGCAGCG :  2860
                                                                                                                                                       
                                                                                                                                                       
                      *      2880         *      2900         *      2920         *      2940         *      2960         *      2980         *        
NODE_10593 : ---------------------------------------------------------------------------------------------------------------------------------- :     -
p201N_cas9 : TACGAAGAAGGCCAAGAACGGCCGCCTGGTGACGGTATCCGAGGGTGAAGCCTTGATTAGCCGCTACAAGATCGTAAAGAGCGAAACCGGGCGGCCGGAGTACATCGAGATCGAGCTAGCTGATTGGATG :  2990
                                                                                                                                                       
                                                                                                                                                       
                   3000         *      3020         *      3040         *      3060         *      3080         *      3100         *      3120        
NODE_10593 : ---------------------------------------------------------------------------------------------------------------------------------- :     -
p201N_cas9 : TACCGCGAGATCACAGAAGGCAAGAACCCGGACGTGCTGACGGTTCACCCCGATTACTTTTTGATCGATCCCGGCATCGGCCGTTTTCTCTACCGCCTGGCACGCCGCGCCGCAGGCAAGGCAGAAGCCA :  3120
                                                                                                                                                       
                                                                                                                                                       
                      *      3140         *      3160         *      3180         *      3200         *      3220         *      3240         *        
NODE_10593 : ---------------------------------------------------------------------------------------------------------------------------------- :     -
p201N_cas9 : GATGGTTGTTCAAGACGATCTACGAACGCAGTGGCAGCGCCGGAGAGTTCAAGAAGTTCTGTTTCACCGTGCGCAAGCTGATCGGGTCAAATGACCTGCCGGAGTACGATTTGAAGGAGGAGGCGGGGCA :  3250
                                                                                                                                                       
                                                                                                                                                       
                   3260         *      3280         *      3300         *      3320         *      3340         *      3360         *      3380        
NODE_10593 : ---------------------------------------------------------------------------------------------------------------------------------- :     -
p201N_cas9 : GGCTGGCCCGATCCTAGTCATGCGCTACCGCAACCTGATCGAGGGCGAAGCATCCGCCGGTTCCTAATGTACGGAGCAGATGCTAGGGCAAATTGCCCTAGCAGGGGAAAAAGGTCGAAAAGGTCTCTTT :  3380
                                                                                                                                                       
                                                                                                                                                       
                      *      3400         *      3420         *      3440         *      3460         *      3480         *      3500         *        
NODE_10593 : ---------------------------------------------------------------------------------------------------------------------------------- :     -
p201N_cas9 : CCTGTGGATAGCACGTACATTGGGAACCCAAAGCCGTACATTGGGAACCGGAACCCGTACATTGGGAACCCAAAGCCGTACATTGGGAACCGGTCACACATGTAAGTGACTGATATAAAAGAGAAAAAAG :  3510
                                                                                                                                                       
                                                                                                                                                       
                   3520         *      3540         *      3560         *      3580         *      3600         *      3620         *      3640        
NODE_10593 : ---------------------------------------------------------------------------------------------------------------------------------- :     -
p201N_cas9 : GCGATTTTTCCGCCTAAAACTCTTTAAAACTTATTAAAACTCTTAAAACCCGCCTGGCCTGTGCATAACTGTCTGGCCAGCGCACAGCCGAAGAGCTGCAAAAAGCGCCTACCCTTCGGTCGCTGCGCTC :  3640
                                                                                                                                                       
                                                                                                                                                       
                      *      3660         *      3680         *      3700         *      3720         *      3740         *      3760         *        
NODE_10593 : ---------------------------------------------------------------------------------------------------------------------------------- :     -
p201N_cas9 : CCTACGCCCCGCCGCTTCGCGTCGGCCTATCGCGGCCGCTGGCCGCTCAAAAATGGCTGGCCTACGGCCAGGCAATCTACCAGGGCGCGGACAAGCCGCGCCGTCGCCACTCGACCGCCGGCGCCCACAT :  3770
                                                                                                                                                       
                                                                                                                                                       
                   3780         *      3800         *      3820         *      3840         *      3860         *      3880         *      3900        
NODE_10593 : ---------------------------------------------------------------------------------------------------------------------------------- :     -
p201N_cas9 : CAAGGCACCCTGCCTCGCGCGTTTCGGTGATGACGGTGAAAACCTCTGACACATGCAGCTCCCGGAGACGGTCACAGCTTGTCTGTAAGCGGATGCCGGGAGCAGACAAGCCCGTCAGGGCGCGTCAGCG :  3900
                                                                                                                                                       
                                                                                                                                                       
                      *      3920         *      3940         *      3960         *      3980         *      4000         *      4020         *        
NODE_10593 : ---------------------------------------------------------------------------------------------------------------------------------- :     -
p201N_cas9 : GGTGTTGGCGGGTGTCGGGGCGCAGCCATGACCCAGTCACGTAGCGATAGCGGAGTGTATACTGGCTTAACTATGCGGCATCAGAGCAGATTGTACTGAGAGTGCACCATATGCGGTGTGAAATACCGCA :  4030
                                                                                                                                                       
                                                                                                                                                       
                   4040         *      4060         *      4080         *      4100         *      4120         *      4140         *      4160        
NODE_10593 : ---------------------------------------------------------------------------------------------------------------------------------- :     -
p201N_cas9 : CAGATGCGTAAGGAGAAAATACCGCATCAGGCGCTCTTCCGCTTCCTCGCTCACTGACTCGCTGCGCTCGGTCGTTCGGCTGCGGCGAGCGGTATCAGCTCACTCAAAGGCGGTAATACGGTTATCCACA :  4160
                                                                                                                                                       
                                                                                                                                                       
                      *      4180         *      4200         *      4220         *      4240         *      4260         *      4280         *        
NODE_10593 : ---------------------------------------------------------------------------------------------------------------------------------- :     -
p201N_cas9 : GAATCAGGGGATAACGCAGGAAAGAACATGTGAGCAAAAGGCCAGCAAAAGGCCAGGAACCGTAAAAAGGCCGCGTTGCTGGCGTTTTTCCATAGGCTCCGCCCCCCTGACGAGCATCACAAAAATCGAC :  4290
                                                                                                                                                       
                                                                                                                                                       
                   4300         *      4320         *      4340         *      4360         *      4380         *      4400         *      4420        
NODE_10593 : ---------------------------------------------------------------------------------------------------------------------------------- :     -
p201N_cas9 : GCTCAAGTCAGAGGTGGCGAAACCCGACAGGACTATAAAGATACCAGGCGTTTCCCCCTGGAAGCTCCCTCGTGCGCTCTCCTGTTCCGACCCTGCCGCTTACCGGATACCTGTCCGCCTTTCTCCCTTC :  4420
                                                                                                                                                       
                                                                                                                                                       
                      *      4440         *      4460         *      4480         *      4500         *      4520         *      4540         *        
NODE_10593 : ---------------------------------------------------------------------------------------------------------------------------------- :     -
p201N_cas9 : GGGAAGCGTGGCGCTTTCTCATAGCTCACGCTGTAGGTATCTCAGTTCGGTGTAGGTCGTTCGCTCCAAGCTGGGCTGTGTGCACGAACCCCCCGTTCAGCCCGACCGCTGCGCCTTATCCGGTAACTAT :  4550
                                                                                                                                                       
                                                                                                                                                       
                   4560         *      4580         *      4600         *      4620         *      4640         *      4660         *      4680        
NODE_10593 : ---------------------------------------------------------------------------------------------------------------------------------- :     -
p201N_cas9 : CGTCTTGAGTCCAACCCGGTAAGACACGACTTATCGCCACTGGCAGCAGCCACTGGTAACAGGATTAGCAGAGCGAGGTATGTAGGCGGTGCTACAGAGTTCTTGAAGTGGTGGCCTAACTACGGCTACA :  4680
                                                                                                                                                       
                                                                                                                                                       
                      *      4700         *      4720         *      4740         *      4760         *      4780         *      4800         *        
NODE_10593 : ---------------------------------------------------------------------------------------------------------------------------------- :     -
p201N_cas9 : CTAGAAGGACAGTATTTGGTATCTGCGCTCTGCTGAAGCCAGTTACCTTCGGAAAAAGAGTTGGTAGCTCTTGATCCGGCAAACAAACCACCGCTGGTAGCGGTGGTTTTTTTGTTTGCAAGCAGCAGAT :  4810
                                                                                                                                                       
                                                                                                                                                       
                   4820         *      4840         *      4860         *      4880         *      4900         *      4920         *      4940        
NODE_10593 : ---------------------------------------------------------------------------------------------------------------------------------- :     -
p201N_cas9 : TACGCGCAGAAAAAAAGGATCTCAAGAAGATCCTTTGATCTTTTCTACGGGGTCTGACGCTCAGTGGAACGAAAACTCACGTTAAGGGATTTTGGTCATGCAGGATCATGAATTAATTCTTAGAAAAACT :  4940
                                                                                                                                                       
                                                                                                                                                       
                      *      4960         *      4980         *      5000         *      5020         *      5040         *      5060         *        
NODE_10593 : ---------------------------------------------------------------------------------------------------------------------------------- :     -
p201N_cas9 : CATCGAGCATCAAATGAAACTGCAATTTATTCATATCAGGATTATCAATACCATATTTTTGAAAAAGCCGTTTCTGTAATGAAGGAGAAAACTCACCGAGGCAGTTCCATAGGATGGCAAGATCCTGGTA :  5070
                                                                                                                                                       
                                                                                                                                                       
                   5080         *      5100         *      5120         *      5140         *      5160         *      5180         *      5200        
NODE_10593 : ---------------------------------------------------------------------------------------------------------------------------------- :     -
p201N_cas9 : TCGGTCTGCGATTCCGACTCGTCCAACATCAATACAACCTATTAATTTCCCCTCGTCAAAAATAAGGTTATCAAGTGAGAAATCACCATGAGTGACGACTGAATCCGGTGAGAATGGCAAAAGTTTATGC :  5200
                                                                                                                                                       
                                                                                                                                                       
                      *      5220         *      5240         *      5260         *      5280         *      5300         *      5320         *        
NODE_10593 : ---------------------------------------------------------------------------------------------------------------------------------- :     -
p201N_cas9 : ATTTCTTTCCAGACTTGTTCAACAGGCCAGCCATTACGCTCGTCATCAAAATCACTCGCATCAACCAAACCGTTATTCATTCGTGATTGCGCCTGAGCGAGACGAAATACGCGATCGCTGTTAAAAGGAC :  5330
                                                                                                                                                       
                                                                                                                                                       
                   5340         *      5360         *      5380         *      5400         *      5420         *      5440         *      5460        
NODE_10593 : ---------------------------------------------------------------------------------------------------------------------------------- :     -
p201N_cas9 : AATTACAAACAGGAATCGAATGCAACCGGCGCAGGAACACTGCCAGCGCATCAACAATATTTTCACCTGAATCAGGATATTCTTCTAATACCTGGAATGCTGTTTTCCCGGGGATCGCAGTGGTGAGTAA :  5460
                                                                                                                                                       
                                                                                                                                                       
                      *      5480         *      5500         *      5520         *      5540         *      5560         *      5580         *        
NODE_10593 : ---------------------------------------------------------------------------------------------------------------------------------- :     -
p201N_cas9 : CCATGCATCATCAGGAGTACGGATAAAATGCTTGATGGTCGGAAGAGGCATAAATTCCGTCAGCCAGTTTAGTCTGACCATCTCATCTGTAACATCATTGGCAACGCTACCTTTGCCATGTTTCAGAAAC :  5590
                                                                                                                                                       
                                                                                                                                                       
                   5600         *      5620         *      5640         *      5660         *      5680         *      5700         *      5720        
NODE_10593 : ---------------------------------------------------------------------------------------------------------------------------------- :     -
p201N_cas9 : AACTCTGGCGCATCGGGCTTCCCATACAATCGATAGATTGTCGCACCTGATTGCCCGACATTATCGCGAGCCCATTTATACCCATATAAATCAGCATCCATGTTGGAATTTAATCGCGGCCTAGAGCAAG :  5720
                                                                                                                                                       
                                                                                                                                                       
                      *      5740         *      5760         *      5780         *      5800         *      5820         *      5840         *        
NODE_10593 : ---------------------------------------------------------------------------------------------------------------------------------- :     -
p201N_cas9 : ACGTTTCCCGTTGAATATGGCTCATAACACCCCTTGTATTACTGTTTATGTAAGCAGACAGTTTTATTGTTCATGATCTGGATCACAGGCAGCAACGCTCTGTCATCGTTACAATCAACATGCTACCCTC :  5850
                                                                                                                                                       
                                                                                                                                                       
                   5860         *      5880         *      5900         *      5920         *      5940         *      5960         *      5980        
NODE_10593 : ---------------------------------------------------------------------------------------------------------------------------------- :     -
p201N_cas9 : CGCGAGATCATCCGTGTTTCAAACCCGGCAGCTTAGTTGCCGTTCTTCCGAATAGCATCGGTAACATGAGCAAAGTCTGCCGCCTTACAACGGCTCTCCCGCTGACGCCGTCCCGGACTGATGGGCTGCC :  5980
                                                                                                                                                       
                                                                                                                                                       
                      *      6000         *      6020         *      6040         *      6060         *      6080         *      6100         *        
NODE_10593 : ---------------------------------------------------------------------------------------------------------------------------------- :     -
p201N_cas9 : TGTATCGAGTGGTGATTTTGTGCCGAGCTGCCGGTCGGGGAGCTGTTGGCTGGCTGGTGGCAGGATATATTGTGGTGTAAACAAATTGACGCTTAGACAACTTAATAACACATTGCGGACGTTTTTAATG :  6110
                                                                                                                                                       
                                                                                                                                                       
                   6120         *      6140         *      6160         *      6180         *      6200         *      6220         *      6240        
NODE_10593 : ---------------------------------------------------------------------------------------------------------------------------------- :     -
p201N_cas9 : TACTGAATTAACGCCGAATTGCTCTAGCCAATACGCAAACCGCCTCTCCCCGCGCGTTGGCCGATTCATTAATGCAGCTGGCACGACAGGTTTCCCGACTGGAAAGCGGGCAGTGAGCGCAACGCAATTA :  6240
                                                                                                                                                       
                                                                                                                                                       
                      *      6260         *      6280         *      6300         *      6320         *      6340         *      6360         *        
NODE_10593 : ---------------------------------------------------------------------------------------------------------------------------------- :     -
p201N_cas9 : ATGTGAGTTAGCTCACTCATTAGGCACCCCAGGCTTTACACTTTATGCTTCCGGCTCGTATGTTGTGTGGAATTGTGAGCGGATAACAATTTCACACAGGAAACAGCTATGACATGATTACGAATTCCTT :  6370
                                                                                                                                                       
                                                                                                                                                       
                   6380         *      6400         *      6420         *      6440         *      6460         *      6480         *      6500        
NODE_10593 : ---------------------------------------------------------------------------------------------------------------------------------- :     -
p201N_cas9 : GACTAGAGGGTAGGCGCGGCTTAATTAAGCCGCGCCGTACCCCAATAGTCTCGACAGACACATAGCACCTAACATTATTGCTTAAATTTACTATTTTTTTACTATATTATACTCAACCCAATGAGCATAA :  6500
                                                                                                                                                       
                                                                                                                                                       
                      *      6520         *      6540         *      6560         *      6580         *      6600         *      6620         *        
NODE_10593 : ---------------------------------------------------------------------------------------------------------------------------------- :     -
p201N_cas9 : AGACTGTAAAATCTCAAATTCCTGAGAAGCATATTTATCGATCCCACAGACTTGATAGTTCCATAATCCATACGCTGCAGCCAAATTGCTAGTGTGTTGAACATTTAACACGTAGAGAACTAGAAAAGAT :  6630
                                                                                                                                                       
                                                                                                                                                       
                   6640         *      6660         *      6680         *      6700         *      6720         *      6740         *      6760        
NODE_10593 : ---------------------------------------------------------------------------------------------------------------------------------- :     -
p201N_cas9 : ATAAAACTAAGATTGATATCCAAAATAGACGAGAACAATAAGCAAAAACTCTTAGTTTTGAAATAAATCAACAATCCCGAGGGTTGTCACATACATCAAAAACGAAAATCCATATAGCAAAAAAAACTCT :  6760
                                                                                                                                                       
                                                                                                                                                       
                      *      6780         *      6800         *      6820         *      6840         *      6860         *      6880         *        
NODE_10593 : ---------------------------------------------------------------------------------------------------------------------------------- :     -
p201N_cas9 : AAATTACCGTTCGACAAAAAGAGAAAACTGATAGGACATTTGCTAAACATTAAAATCAAAATTTGGATCAAGCCGAATTCTCAGAAGAACTCGTCAAGAAGGCGATAGAAGGCGATGCGCTGCGAATCGG :  6890
                                                                                                                                                       
                                                                                                                                                       
                   6900         *      6920         *      6940         *      6960         *      6980         *      7000         *      7020        
NODE_10593 : ---------------------------------------------------------------------------------------------------------------------------------- :     -
p201N_cas9 : GAGCGGCGATACCGTAAAGCACGAGGAAGCGGTCAGCCCATTCGCCGCCAAGCTCTTCAGCAATATCACGGGTAGCCAACGCTATGTCCTGATAGCGGTCCGCCACACCCAGCCGGCCACAGTCGATGAA :  7020
                                                                                                                                                       
                                                                                                                                                       
                      *      7040         *      7060         *      7080         *      7100         *      7120         *      7140         *        
NODE_10593 : ---------------------------------------------------------------------------------------------------------------------------------- :     -
p201N_cas9 : TCCAGAAAAGCGGCCATTTTCCACCATGATATTCGGCAAGCAGGCATCGCCATGGGTCACGACGAGATCATCGCCGTCGGGCATGCGCGCCTTGAGCCTGGCGAACAGTTCGGCTGGCGCGAGCCCCTGA :  7150
                                                                                                                                                       
                                                                                                                                                       
                   7160         *      7180         *      7200         *      7220         *      7240         *      7260         *      7280        
NODE_10593 : ---------------------------------------------------------------------------------------------------------------------------------- :     -
p201N_cas9 : TGCTCTTCGTCCAGATCATCCTGATCGACAAGACCGGCTTCCATCCGAGTACGTGCTCGCTCGATGCGATGTTTCGCTTGGTGGTCGAATGGGCAGGTAGCCGGATCAAGCGTATGCAGCCGCCGCATTG :  7280
                                                                                                                                                       
                                                                                                                                                       
                      *      7300         *      7320         *      7340         *      7360         *      7380         *      7400         *        
NODE_10593 : ---------------------------------------------------------------------------------------------------------------------------------- :     -
p201N_cas9 : CATCAGCCATGATGGATACTTTCTCGGCAGGAGCAAGGTGAGATGACAGGAGATCCTGCCCCGGCACTTCGCCCAATAGCAGCCAGTCCCTTCCCGCTTCAGTGACAACGTCGAGCACAGCTGCGCAAGG :  7410
                                                                                                                                                       
                                                                                                                                                       
                   7420         *      7440         *      7460         *      7480         *      7500         *      7520         *      7540        
NODE_10593 : ---------------------------------------------------------------------------------------------------------------------------------- :     -
p201N_cas9 : AACGCCCGTCGTGGCCAGCCACGATAGCCGCGCTGCCTCGTCCTGCAGTTCATTCAGGGCACCGGACAGGTCGGTCTTGACAAAAAGAACCGGGCGCCCCTGCGCTGACAGCCGGAACACGGCGGCATCA :  7540
                                                                                                                                                       
                                                                                                                                                       
                      *      7560         *      7580         *      7600         *      7620         *      7640         *      7660         *        
NODE_10593 : ---------------------------------------------------------------------------------------------------------------------------------- :     -
p201N_cas9 : GAGCAGCCGATTGTCTGTTGTGCCCAGTCATAGCCGAATAGCCTCTCCACCCAAGCGGCCGGAGAACCTGCGTGCAATCCATCTTGTTCAATCATCTCGAGACGTACCGCGCGATGCATTCGAAGATCGA :  7670
                                                                                                                                                       
                                                                                                                                                       
                   7680         *      7700         *      7720         *      7740         *      7760         *      7780         *      7800        
NODE_10593 : ---------------------------------------------------------------------------------------------------------------------------------- :     -
p201N_cas9 : TCCTTCGCCTGGAGGAGAGAAATCAGTGGCGCTGCGGCTTTTAGGGTTTCTTTGTTGATGGAATGAGAGTGTAAGCTCTGCCAGTGCCACTTTATTAGGGTTTTACAAGCCCTTTTCTTCGTAATTGGGC :  7800
                                                                                                                                                       
                                                                                                                                                       
                      *      7820         *      7840         *      7860         *      7880         *      7900         *      7920         *        
NODE_10593 : ---------------------------------------------------------------------------------------------------------------------------------- :     -
p201N_cas9 : CTGACATTTTGTGCCACTTGGGCCTTTAGAGATGAAAATGTATATTGGGCTTAAGTTGACTTGAAGGATAAATTAGTTTAGGATATTACGTTTTTTATGAGAATTGGTGTGTCGGATACATGTATATGAT :  7930
                                                                                                                                                       
                                                                                                                                                       
                   7940         *      7960         *      7980         *      8000         *      8020         *      8040         *      8060        
NODE_10593 : ---------------------------------------------------------------------------------------------AAGAAGAATATTACTCCTTTAATAGCTTTTGAGTATA :    37
p201N_cas9 : GCATTCAAATATATGTATTCTAGATACATTTAAGTTTAGATACAATCTAAAATGTGTCTTTAATTACAGGACTGTAACTAAAATACTTAATGTAAGAAGAATATTACTCCTTTAATAGCTTTTGAGTATA :  8060
                                                                                                          AAGAAGAATATTACTCCTTTAATAGCTTTTGAGTATA        
                                                                                                                                                       
                      *      8080         *      8100         *      8120         *      8140         *      8160         *      8180         *        
NODE_10593 : TCTAGTCTAACATCTTTTAAAAAAGTCTAATTTCTTTCATTTATTTTTCGAGCAATAGCAAAGTGCATAATTATTTTTTTCTTCTAGAAATTCAGATTTGTTTCTCTAAATTTTGAGATTCTTTTCTCAA :   167
p201N_cas9 : TCTAGTCTAACATCTTTTAAAAAAGTCTAATTTCTTTCATTTATTTTTCGAGCAATAGCAAAGTGCATAATTATTTTTTTCTTCTAGAAATTCAGATTTGTTTCTCTAAATTTTGAGATTCTTTTCTCAA :  8190
             TCTAGTCTAACATCTTTTAAAAAAGTCTAATTTCTTTCATTTATTTTTCGAGCAATAGCAAAGTGCATAATTATTTTTTTCTTCTAGAAATTCAGATTTGTTTCTCTAAATTTTGAGATTCTTTTCTCAA        
                                                                                                                                                       
                   8200         *      8220         *      8240         *      8260         *      8280         *      8300         *      8320        
NODE_10593 : TTTTGTATGTCTAGAGAACAATGTGTATTTTTCACTCTAGTTGGTTGTTACTTTGTTGAATGTTCTGATAAAAGTATATTGTTATTTCTGAAGTAGATATAAACCTTCATTTGGAAATTATACATAAATC :   297
p201N_cas9 : TTTTGTATGTCTAGAGAACAATGTGTATTTTTCACTCTAGTTGGTTGTTACTTTGTTGAATGTTCTGATAAAAGTATATTGTTATTTCTGAAGTAGATATAAACCTTCATTTGGAAATTATACATAAATC :  8320
             TTTTGTATGTCTAGAGAACAATGTGTATTTTTCACTCTAGTTGGTTGTTACTTTGTTGAATGTTCTGATAAAAGTATATTGTTATTTCTGAAGTAGATATAAACCTTCATTTGGAAATTATACATAAATC        
                                                                                                                                                       
                      *      8340         *      8360         *      8380         *      8400         *      8420         *      8440         *        
NODE_10593 : AAAATCGTTAATTATCTAGATCAAGATATATGCCCTTTTCCTAATGTATTTGATACATGCACCTAATTTCACTAGATGTATCTTTTCTATTTTTTAAATTATGAATAGTTAATTTTTTCCATATGTGTAT :   427
p201N_cas9 : AAAATCGTTAATTATCTAGATCAAGATATATGCCCTTTTCCTAATGTATTTGATACATGCACCTAATTTCACTAGATGTATCTTTTCTATTTTTTAAATTATGAATAGTTAATTTTTTCCATATGTGTAT :  8450
             AAAATCGTTAATTATCTAGATCAAGATATATGCCCTTTTCCTAATGTATTTGATACATGCACCTAATTTCACTAGATGTATCTTTTCTATTTTTTAAATTATGAATAGTTAATTTTTTCCATATGTGTAT        
                                                                                                                                                       
                   8460         *      8480         *      8500         *      8520         *      8540         *      8560         *      8580        
NODE_10593 : TTGATACATACTTCATGACTTTAAAAAATTAATTATATACCAGATATATGTATTTAAAATTTGTTATGTATTTAAAGTATGTATATGATTATTCGATATTAATCTCTTCGATGAAATTTA---------- :   547
p201N_cas9 : TTGATACATACTTCATGACTTTAAAAAATTAATTATATACCAGATATATGTATTTAAAATTTGTTATGTATTTAAAGTATGTATATGATTATTCGATATTAATCTCTTCGATGAAATTTATGCCTATCTT :  8580
             TTGATACATACTTCATGACTTTAAAAAATTAATTATATACCAGATATATGTATTTAAAATTTGTTATGTATTTAAAGTATGTATATGATTATTCGATATTAATCTCTTCGATGAAATTTA                  
                                                                                                                                                       
                      *      8600         *      8620         *      8640         *      8660         *      8680         *      8700         *        
NODE_10593 : ----AATCGATAA--------------------------------------------------------------------------------------------------------------------- :   556
p201N_cas9 : ATATGATCAATGAGGCATTTAATTGGGTGCATATGATGGTGAAAAAAGGTGCAGCTCCTGGCTTGGGAATGATGACTCATGTGGAATTTGGTCTTAAATTTATCACATCCTTTTGGGATGTGATGATTGT :  8710
                  ATC AT A                                                                                                                             
                                                                                                                                                       
                   8720         *      8740         *      8760         *      8780         *      8800         *      8820         *      8840        
NODE_10593 : -----------------------------------CTATGTGCTTTGGATCGATCTG----------------------------------CCCACTAG------------------------------- :   586
p201N_cas9 : ATCACTTGTTCATTTTGCAAAGACAAGGTGCACTGCTACAAACTTTGGTTTAATCTGAAATAAAACAAAACTCACTGAGAGGAAGATGCATCCCAGTAGGTGAAAGTCGAGAAGGATTTGCATGTTACTA :  8840
                                                CTA    CTTTGG T  ATCTG                                  CCCA TAG                                       
                                                                                                                                                       
                      *      8860         *      8880         *      8900         *      8920         *      8940         *      8960         *        
NODE_10593 : ---------------------------------------------------------------------------------------------------------------------------------- :     -
p201N_cas9 : TTACACTTGCTTTTTAGTCCCACATCGTCTGAAACATAAAATATTTCAGCGTTTAAATACTTCAAGCGAACCAGTAGGCTTGTTTTAGAGCTAGAAATAGCAAGTTAAAATAAGGCTAGTCCGTTATCAA :  8970
                                                                                                                                                       
                                                                                                                                                       
                   8980         *      9000         *      9020         *      9040         *      9060         *      9080         *      9100        
NODE_10593 : ----------------------------------TGAGTCGTATTACAATTCATGACTCTCTTAAGGTAGCCAAAATCGATATCTAGGGATAACAGGGTAATCGATCACTAGAATTCGGCGCGCCGGGCC :   682
p201N_cas9 : CTTGAAAAAGTGGCACCGAGTCGGTGCTTTTTTTTGAGTCGTATTACAATTCATGACTCTCTTAAGGTAGCCAAAATCGATATCTAGGGATAACAGGGTAATCGATCACTAGAATTCGGCGCGCCGGGCC :  9100
                                               TGAGTCGTATTACAATTCATGACTCTCTTAAGGTAGCCAAAATCGATATCTAGGGATAACAGGGTAATCGATCACTAGAATTCGGCGCGCCGGGCC        
                                                                                                                                                       
                      *      9120         *      9140         *      9160         *      9180         *      9200         *      9220         *        
NODE_10593 : CAACATGGTGGAGCACGACACTCTCGTCTACTCCAAGAATATCAAAGATACAGTCTCAGAAGACCAAAGGGCTATTGAGACTTTTCAACAAAGGGTAATATCGGGAAACCTCCTCGGATTCCATTGCCCA :   812
p201N_cas9 : CAACATGGTGGAGCACGACACTCTCGTCTACTCCAAGAATATCAAAGATACAGTCTCAGAAGACCAAAGGGCTATTGAGACTTTTCAACAAAGGGTAATATCGGGAAACCTCCTCGGATTCCATTGCCCA :  9230
             CAACATGGTGGAGCACGACACTCTCGTCTACTCCAAGAATATCAAAGATACAGTCTCAGAAGACCAAAGGGCTATTGAGACTTTTCAACAAAGGGTAATATCGGGAAACCTCCTCGGATTCCATTGCCCA        
                                                                                                                                                       
                   9240         *      9260         *      9280         *      9300         *      9320         *      9340         *      9360        
NODE_10593 : GCTATCTGTCACTTCATCAAAAGGACAGTAGAAAAGGAAGGTGGCACCTACAAATGCCATCATTGCGATAAAGGAAAGGCTATCGTTCAAGATGCCTCTGCCGACAGTGGTCCCAAAGATGGACCCCCAC :   942
p201N_cas9 : GCTATCTGTCACTTCATCAAAAGGACAGTAGAAAAGGAAGGTGGCACCTACAAATGCCATCATTGCGATAAAGGAAAGGCTATCGTTCAAGATGCCTCTGCCGACAGTGGTCCCAAAGATGGACCCCCAC :  9360
             GCTATCTGTCACTTCATCAAAAGGACAGTAGAAAAGGAAGGTGGCACCTACAAATGCCATCATTGCGATAAAGGAAAGGCTATCGTTCAAGATGCCTCTGCCGACAGTGGTCCCAAAGATGGACCCCCAC        
                                                                                                                                                       
                      *      9380         *      9400         *      9420         *      9440         *      9460         *      9480         *        
NODE_10593 : CCACGAGGAGCATCGTGGAAAAAGAAGACGTTCCAACCACGTCTTCAAAGCAAGTGGATTGATGTGA--------------------------------------------------------------- :  1009
p201N_cas9 : CCACGAGGAGCATCGTGGAAAAAGAAGACGTTCCAACCACGTCTTCAAAGCAAGTGGATTGATGTGAACATGGTGGAGCACGACACTCTCGTCTACTCCAAGAATATCAAAGATACAGTCTCAGAAGACC :  9490
             CCACGAGGAGCATCGTGGAAAAAGAAGACGTTCCAACCACGTCTTCAAAGCAAGTGGATTGATGTGA                                                                       
                                                                                                                                                       
                   9500         *      9520         *      9540         *      9560         *      9580         *      9600         *      9620        
NODE_10593 : ---------------------------------------------------------------------------------------------------------------------------------- :     -
p201N_cas9 : AAAGGGCTATTGAGACTTTTCAACAAAGGGTAATATCGGGAAACCTCCTCGGATTCCATTGCCCAGCTATCTGTCACTTCATCAAAAGGACAGTAGAAAAGGAAGGTGGCACCTACAAATGCCATCATTG :  9620
                                                                                                                                                       
                                                                                                                                                       
                      *      9640         *      9660         *      9680         *      9700         *      9720         *      9740         *        
NODE_10593 : ---------------------------------------------------------------------------------------------------------------------------------- :     -
p201N_cas9 : CGATAAAGGAAAGGCTATCGTTCAAGATGCCTCTGCCGACAGTGGTCCCAAAGATGGACCCCCACCCACGAGGAGCATCGTGGAAAAAGAAGACGTTCCAACCACGTCTTCAAAGCAAGTGGATTGATGT :  9750
                                                                                                                                                       
                                                                                                                                                       
                   9760         *      9780         *      9800         *      9820         *      9840         *      9860         *      9880        
NODE_10593 : --TATCTCCACTGACGTAAGGGATGACGCACAATCCCACTATCCTTCGCAAGACCCTTCCTCTATATAAGGAAGTTCATTTCATTTGGAGAGGACACGCTGAAATCACCAGTCTCTCTCTACAAATCTAT :  1137
p201N_cas9 : GATATCTCCACTGACGTAAGGGATGACGCACAATCCCACTATCCTTCGCAAGACCCTTCCTCTATATAAGGAAGTTCATTTCATTTGGAGAGGACACGCTGAAATCACCAGTCTCTCTCTACAAATCTAT :  9880
               TATCTCCACTGACGTAAGGGATGACGCACAATCCCACTATCCTTCGCAAGACCCTTCCTCTATATAAGGAAGTTCATTTCATTTGGAGAGGACACGCTGAAATCACCAGTCTCTCTCTACAAATCTAT        
                                                                                                                                                       
                      *      9900         *      9920         *      9940         *      9960         *      9980         *     10000         *        
NODE_10593 : CTCTCTCGACCGCTAGCATGGACAAGAAGTACTCCATTGGGCTCGATATCGGCACAAACAGCGTCGGCTGGGCCGTCATTACGGACGAGTACAAGGTGCCGAGCAAAAAATTCAAAGTTCTGGGCAATAC :  1267
p201N_cas9 : CTCTCTCGACCGCTAGCATGGACAAGAAGTACTCCATTGGGCTCGATATCGGCACAAACAGCGTCGGCTGGGCCGTCATTACGGACGAGTACAAGGTGCCGAGCAAAAAATTCAAAGTTCTGGGCAATAC : 10010
             CTCTCTCGACCGCTAGCATGGACAAGAAGTACTCCATTGGGCTCGATATCGGCACAAACAGCGTCGGCTGGGCCGTCATTACGGACGAGTACAAGGTGCCGAGCAAAAAATTCAAAGTTCTGGGCAATAC        
                                                                                                                                                       
                  10020         *     10040         *     10060         *     10080         *     10100         *     10120         *     10140        
NODE_10593 : CGATCGCCACAGCATAAAGAAGAACCTCATTGGCGCCCTCCTGTTCGACTCCGGGGAGACGGCCGAAGCCACGCGGCTCAAAAGAACAGCACGGCGCAGATATACCCGCAGAAAGAATCGGATCTGCTAC :  1397
p201N_cas9 : CGATCGCCACAGCATAAAGAAGAACCTCATTGGCGCCCTCCTGTTCGACTCCGGGGAGACGGCCGAAGCCACGCGGCTCAAAAGAACAGCACGGCGCAGATATACCCGCAGAAAGAATCGGATCTGCTAC : 10140
             CGATCGCCACAGCATAAAGAAGAACCTCATTGGCGCCCTCCTGTTCGACTCCGGGGAGACGGCCGAAGCCACGCGGCTCAAAAGAACAGCACGGCGCAGATATACCCGCAGAAAGAATCGGATCTGCTAC        
                                                                                                                                                       
                      *     10160         *     10180         *     10200         *     10220         *     10240         *     10260         *        
NODE_10593 : CTGCAGGAGATCTTTAGTAATGAGATGGCTAAGGTGGATGACTCTTTCTTCCATAGGCTGGAGGAGTCCTTTTTGGTGGAGGAGGATAAAAAGCACGAGCGCCACCCAATCTTTGGCAATATCGTGGACG :  1527
p201N_cas9 : CTGCAGGAGATCTTTAGTAATGAGATGGCTAAGGTGGATGACTCTTTCTTCCATAGGCTGGAGGAGTCCTTTTTGGTGGAGGAGGATAAAAAGCACGAGCGCCACCCAATCTTTGGCAATATCGTGGACG : 10270
             CTGCAGGAGATCTTTAGTAATGAGATGGCTAAGGTGGATGACTCTTTCTTCCATAGGCTGGAGGAGTCCTTTTTGGTGGAGGAGGATAAAAAGCACGAGCGCCACCCAATCTTTGGCAATATCGTGGACG        
                                                                                                                                                       
                  10280         *     10300         *     10320         *     10340         *     10360         *     10380         *     10400        
NODE_10593 : AGGTGGCGTACCATGAAAAGTACCCAACCATATATCATCTGAGGAAGAAGCTTGTAGACAGTACTGATAAGGCTGACTTGCGGTTGATCTATCTCGCGCTGGCGCATATGATCAAATTTCGGGGACACTT :  1657
p201N_cas9 : AGGTGGCGTACCATGAAAAGTACCCAACCATATATCATCTGAGGAAGAAGCTTGTAGACAGTACTGATAAGGCTGACTTGCGGTTGATCTATCTCGCGCTGGCGCATATGATCAAATTTCGGGGACACTT : 10400
             AGGTGGCGTACCATGAAAAGTACCCAACCATATATCATCTGAGGAAGAAGCTTGTAGACAGTACTGATAAGGCTGACTTGCGGTTGATCTATCTCGCGCTGGCGCATATGATCAAATTTCGGGGACACTT        
                                                                                                                                                       
                      *     10420         *     10440         *     10460         *     10480         *     10500         *     10520         *        
NODE_10593 : CCTCATCGAGGGGGACCTGAACCCAGACAACAGCGATGTCGACAAACTCTTTATCCAACTGGTTCAGACTTACAATCAGCTTTTCGAAGAGAACCCGATCAACGCATCCGGAGTTGACGCCAAAGCAATC :  1787
p201N_cas9 : CCTCATCGAGGGGGACCTGAACCCAGACAACAGCGATGTCGACAAACTCTTTATCCAACTGGTTCAGACTTACAATCAGCTTTTCGAAGAGAACCCGATCAACGCATCCGGAGTTGACGCCAAAGCAATC : 10530
             CCTCATCGAGGGGGACCTGAACCCAGACAACAGCGATGTCGACAAACTCTTTATCCAACTGGTTCAGACTTACAATCAGCTTTTCGAAGAGAACCCGATCAACGCATCCGGAGTTGACGCCAAAGCAATC        
                                                                                                                                                       
                  10540         *     10560         *     10580         *     10600         *     10620         *     10640         *     10660        
NODE_10593 : CTGAGCGCTAGGCTGTCCAAATCCCGGCGGCTCGAAAACCTCATCGCACAGCTCCCTGGGGAGAAGAAGAACGGCCTGTTTGGTAATCTTATCGCCCTGTCACTCGGGCTGACCCCCAACTTTAAATCTA :  1917
p201N_cas9 : CTGAGCGCTAGGCTGTCCAAATCCCGGCGGCTCGAAAACCTCATCGCACAGCTCCCTGGGGAGAAGAAGAACGGCCTGTTTGGTAATCTTATCGCCCTGTCACTCGGGCTGACCCCCAACTTTAAATCTA : 10660
             CTGAGCGCTAGGCTGTCCAAATCCCGGCGGCTCGAAAACCTCATCGCACAGCTCCCTGGGGAGAAGAAGAACGGCCTGTTTGGTAATCTTATCGCCCTGTCACTCGGGCTGACCCCCAACTTTAAATCTA        
                                                                                                                                                       
                      *     10680         *     10700         *     10720         *     10740         *     10760         *     10780         *        
NODE_10593 : ACTTCGACCTGGCCGAAGATGCCAAGCTTCAACTGAGCAAAGACACCTACGATGATGATCTCGACAATCTGCTGGCCCAGATCGGCGACCAGTACGCAGACCTTTTTTTGGCGGCAAAGAACCTGTCAGA :  2047
p201N_cas9 : ACTTCGACCTGGCCGAAGATGCCAAGCTTCAACTGAGCAAAGACACCTACGATGATGATCTCGACAATCTGCTGGCCCAGATCGGCGACCAGTACGCAGACCTTTTTTTGGCGGCAAAGAACCTGTCAGA : 10790
             ACTTCGACCTGGCCGAAGATGCCAAGCTTCAACTGAGCAAAGACACCTACGATGATGATCTCGACAATCTGCTGGCCCAGATCGGCGACCAGTACGCAGACCTTTTTTTGGCGGCAAAGAACCTGTCAGA        
                                                                                                                                                       
                  10800         *     10820         *     10840         *     10860         *     10880         *     10900         *     10920        
NODE_10593 : CGCCATTCTGCTGAGTGATATTCTGCGAGTGAACACGGAGATCACCAAAGCTCCGCTGAGCGCTAGTATGATCAAGCGCTATGATGAGCACCACCAAGACTTGACTTTGCTGAAGGCCCTTGTCAGACAG :  2177
p201N_cas9 : CGCCATTCTGCTGAGTGATATTCTGCGAGTGAACACGGAGATCACCAAAGCTCCGCTGAGCGCTAGTATGATCAAGCGCTATGATGAGCACCACCAAGACTTGACTTTGCTGAAGGCCCTTGTCAGACAG : 10920
             CGCCATTCTGCTGAGTGATATTCTGCGAGTGAACACGGAGATCACCAAAGCTCCGCTGAGCGCTAGTATGATCAAGCGCTATGATGAGCACCACCAAGACTTGACTTTGCTGAAGGCCCTTGTCAGACAG        
                                                                                                                                                       
                      *     10940         *     10960         *     10980         *     11000         *     11020         *     11040         *        
NODE_10593 : CAACTGCCTGAGAAGTACAAGGAAATTTTCTTCGATCAGTCTAAAAATGGCTACGCCGGATACATTGACGGCGGAGCAAGCCAGGAGGAATTTTACAAATTTATTAAGCCCATCTTGGAAAAAATGGACG :  2307
p201N_cas9 : CAACTGCCTGAGAAGTACAAGGAAATTTTCTTCGATCAGTCTAAAAATGGCTACGCCGGATACATTGACGGCGGAGCAAGCCAGGAGGAATTTTACAAATTTATTAAGCCCATCTTGGAAAAAATGGACG : 11050
             CAACTGCCTGAGAAGTACAAGGAAATTTTCTTCGATCAGTCTAAAAATGGCTACGCCGGATACATTGACGGCGGAGCAAGCCAGGAGGAATTTTACAAATTTATTAAGCCCATCTTGGAAAAAATGGACG        
                                                                                                                                                       
                  11060         *     11080         *     11100         *     11120         *     11140         *     11160         *     11180        
NODE_10593 : GCACCGAGGAGCTGCTGGTAAAGCTTAACAGAGAAGATCTGTTGCGCAAACAGCGCACTTTCGACAATGGAAGCATCCCCCACCAGATTCACCTGGGCGAACTGCACGCTATCCTCAGGCGGCAAGAGGA :  2437
p201N_cas9 : GCACCGAGGAGCTGCTGGTAAAGCTTAACAGAGAAGATCTGTTGCGCAAACAGCGCACTTTCGACAATGGAAGCATCCCCCACCAGATTCACCTGGGCGAACTGCACGCTATCCTCAGGCGGCAAGAGGA : 11180
             GCACCGAGGAGCTGCTGGTAAAGCTTAACAGAGAAGATCTGTTGCGCAAACAGCGCACTTTCGACAATGGAAGCATCCCCCACCAGATTCACCTGGGCGAACTGCACGCTATCCTCAGGCGGCAAGAGGA        
                                                                                                                                                       
                      *     11200         *     11220         *     11240         *     11260         *     11280         *     11300         *        
NODE_10593 : TTTCTACCCCTTTTTGAAAGATAACAGGGAAAAGATTGAGAAAATCCTCACATTTCGGATACCCTACTATGTAGGCCCCCTCGCCCGGGGAAATTCCAGATTCGCGTGGATGACTCGCAAATCAGAAGAG :  2567
p201N_cas9 : TTTCTACCCCTTTTTGAAAGATAACAGGGAAAAGATTGAGAAAATCCTCACATTTCGGATACCCTACTATGTAGGCCCCCTCGCCCGGGGAAATTCCAGATTCGCGTGGATGACTCGCAAATCAGAAGAG : 11310
             TTTCTACCCCTTTTTGAAAGATAACAGGGAAAAGATTGAGAAAATCCTCACATTTCGGATACCCTACTATGTAGGCCCCCTCGCCCGGGGAAATTCCAGATTCGCGTGGATGACTCGCAAATCAGAAGAG        
                                                                                                                                                       
                  11320         *     11340         *     11360         *     11380         *     11400         *     11420         *     11440        
NODE_10593 : ACCATCACTCCCTGGAACTTCGAGGAAGTCGTGGATAAGGGGGCCTCTGCCCAGTCCTTCATCGAAAGGATGACTAACTTTGATAAAAATCTGCCTAACGAAAAGGTGCTTCCTAAACACTCTCTGCTGT :  2697
p201N_cas9 : ACCATCACTCCCTGGAACTTCGAGGAAGTCGTGGATAAGGGGGCCTCTGCCCAGTCCTTCATCGAAAGGATGACTAACTTTGATAAAAATCTGCCTAACGAAAAGGTGCTTCCTAAACACTCTCTGCTGT : 11440
             ACCATCACTCCCTGGAACTTCGAGGAAGTCGTGGATAAGGGGGCCTCTGCCCAGTCCTTCATCGAAAGGATGACTAACTTTGATAAAAATCTGCCTAACGAAAAGGTGCTTCCTAAACACTCTCTGCTGT        
                                                                                                                                                       
                      *     11460         *     11480         *     11500         *     11520         *     11540         *     11560         *        
NODE_10593 : ACGAGTACTTCACAGTTTATAACGAGCTCACCAAGGTCAAATACGTCACAGAAGGGATGAGAAAGCCAGCATTCCTGTCTGGAGAGCAGAAGAAAGCTATCGTGGACCTCCTCTTCAAGACGAACCGGAA :  2827
p201N_cas9 : ACGAGTACTTCACAGTTTATAACGAGCTCACCAAGGTCAAATACGTCACAGAAGGGATGAGAAAGCCAGCATTCCTGTCTGGAGAGCAGAAGAAAGCTATCGTGGACCTCCTCTTCAAGACGAACCGGAA : 11570
             ACGAGTACTTCACAGTTTATAACGAGCTCACCAAGGTCAAATACGTCACAGAAGGGATGAGAAAGCCAGCATTCCTGTCTGGAGAGCAGAAGAAAGCTATCGTGGACCTCCTCTTCAAGACGAACCGGAA        
                                                                                                                                                       
                  11580         *     11600         *     11620         *     11640         *     11660         *     11680         *     11700        
NODE_10593 : AGTTACCGTGAAACAGCTCAAAGAAGACTATTTCAAAAAGATTGAATGTTTCGACTCTGTTGAAATCAGCGGAGTGGAGGATCGCTTCAACGCATCCCTGGGAACGTATCACGATCTCCTGAAAATCATT :  2957
p201N_cas9 : AGTTACCGTGAAACAGCTCAAAGAAGACTATTTCAAAAAGATTGAATGTTTCGACTCTGTTGAAATCAGCGGAGTGGAGGATCGCTTCAACGCATCCCTGGGAACGTATCACGATCTCCTGAAAATCATT : 11700
             AGTTACCGTGAAACAGCTCAAAGAAGACTATTTCAAAAAGATTGAATGTTTCGACTCTGTTGAAATCAGCGGAGTGGAGGATCGCTTCAACGCATCCCTGGGAACGTATCACGATCTCCTGAAAATCATT        
                                                                                                                                                       
                      *     11720         *     11740         *     11760         *     11780         *     11800         *     11820         *        
NODE_10593 : AAAGACAAGGACTTCCTGGACAATGAGGAGAACGAGGACATTCTTGAGGACATTGTCCTCACCCTTACGTTGTTTGAAGATAGGGAGATGATTGAAGAACGCTTGAAAACTTACGCTCATCTCTTCGACG :  3087
p201N_cas9 : AAAGACAAGGACTTCCTGGACAATGAGGAGAACGAGGACATTCTTGAGGACATTGTCCTCACCCTTACGTTGTTTGAAGATAGGGAGATGATTGAAGAACGCTTGAAAACTTACGCTCATCTCTTCGACG : 11830
             AAAGACAAGGACTTCCTGGACAATGAGGAGAACGAGGACATTCTTGAGGACATTGTCCTCACCCTTACGTTGTTTGAAGATAGGGAGATGATTGAAGAACGCTTGAAAACTTACGCTCATCTCTTCGACG        
                                                                                                                                                       
                  11840         *     11860         *     11880         *     11900         *     11920         *     11940         *     11960        
NODE_10593 : ACAAAGTCATGAAACAGCTCAAGAGGCGCCGATATACAGGATGGGGGCGGCTGTCAAGAAAACTGATCAATGGGATCCGAGACAAGCAGAGTGGAAAGACAATCCTGGATTTTCTTAAGTCCGATGGATT :  3217
p201N_cas9 : ACAAAGTCATGAAACAGCTCAAGAGGCGCCGATATACAGGATGGGGGCGGCTGTCAAGAAAACTGATCAATGGGATCCGAGACAAGCAGAGTGGAAAGACAATCCTGGATTTTCTTAAGTCCGATGGATT : 11960
             ACAAAGTCATGAAACAGCTCAAGAGGCGCCGATATACAGGATGGGGGCGGCTGTCAAGAAAACTGATCAATGGGATCCGAGACAAGCAGAGTGGAAAGACAATCCTGGATTTTCTTAAGTCCGATGGATT        
                                                                                                                                                       
                      *     11980         *     12000         *     12020         *     12040         *     12060         *     12080         *        
NODE_10593 : TGCCAACCGGAACTTCATGCAGTTGATCCATGATGACTCTCTCACCTTTAAGGAGGACATCCAGAAAGCACAAGTTTCTGGCCAGGGGGACAGTCTTCACGAGCACATCGCTAATCTTGCAGGTAGCCCA :  3347
p201N_cas9 : TGCCAACCGGAACTTCATGCAGTTGATCCATGATGACTCTCTCACCTTTAAGGAGGACATCCAGAAAGCACAAGTTTCTGGCCAGGGGGACAGTCTTCACGAGCACATCGCTAATCTTGCAGGTAGCCCA : 12090
             TGCCAACCGGAACTTCATGCAGTTGATCCATGATGACTCTCTCACCTTTAAGGAGGACATCCAGAAAGCACAAGTTTCTGGCCAGGGGGACAGTCTTCACGAGCACATCGCTAATCTTGCAGGTAGCCCA        
                                                                                                                                                       
                  12100         *     12120         *     12140         *     12160         *     12180         *     12200         *     12220        
NODE_10593 : GCTATCAAAAAGGGAATACTGCAGACCGTTAAGGTCGTGGATGAACTCGTCAAAGTAATGGGAAGGCATAAGCCCGAGAATATCGTTATCGAGATGGCCCGAGAGAACCAAACTACCCAGAAGGGACAGA :  3477
p201N_cas9 : GCTATCAAAAAGGGAATACTGCAGACCGTTAAGGTCGTGGATGAACTCGTCAAAGTAATGGGAAGGCATAAGCCCGAGAATATCGTTATCGAGATGGCCCGAGAGAACCAAACTACCCAGAAGGGACAGA : 12220
             GCTATCAAAAAGGGAATACTGCAGACCGTTAAGGTCGTGGATGAACTCGTCAAAGTAATGGGAAGGCATAAGCCCGAGAATATCGTTATCGAGATGGCCCGAGAGAACCAAACTACCCAGAAGGGACAGA        
                                                                                                                                                       
                      *     12240         *     12260         *     12280         *     12300         *     12320         *     12340         *        
NODE_10593 : AGAACAGTAGGGAAAGGATGAAGAGGATTGAAGAGGGTATAAAAGAACTGGGGTCCCAAATCCTTAAGGAACACCCAGTTGAAAACACCCAGCTTCAGAATGAGAAGCTCTACCTGTACTACCTGCAGAA :  3607
p201N_cas9 : AGAACAGTAGGGAAAGGATGAAGAGGATTGAAGAGGGTATAAAAGAACTGGGGTCCCAAATCCTTAAGGAACACCCAGTTGAAAACACCCAGCTTCAGAATGAGAAGCTCTACCTGTACTACCTGCAGAA : 12350
             AGAACAGTAGGGAAAGGATGAAGAGGATTGAAGAGGGTATAAAAGAACTGGGGTCCCAAATCCTTAAGGAACACCCAGTTGAAAACACCCAGCTTCAGAATGAGAAGCTCTACCTGTACTACCTGCAGAA        
                                                                                                                                                       
                  12360         *     12380         *     12400         *     12420         *     12440         *     12460         *     12480        
NODE_10593 : CGGCAGGGACATGTACGTGGATCAGGAACTGGACATCAATCGGCTCTCCGACTACGACGTGGATCATATCGTGCCCCAGTCTTTTCTCAAAGATGATTCTATTGATAATAAAGTGTTGACAAGATCCGAT :  3737
p201N_cas9 : CGGCAGGGACATGTACGTGGATCAGGAACTGGACATCAATCGGCTCTCCGACTACGACGTGGATCATATCGTGCCCCAGTCTTTTCTCAAAGATGATTCTATTGATAATAAAGTGTTGACAAGATCCGAT : 12480
             CGGCAGGGACATGTACGTGGATCAGGAACTGGACATCAATCGGCTCTCCGACTACGACGTGGATCATATCGTGCCCCAGTCTTTTCTCAAAGATGATTCTATTGATAATAAAGTGTTGACAAGATCCGAT        
                                                                                                                                                       
                      *     12500         *     12520         *     12540         *     12560         *     12580         *     12600         *        
NODE_10593 : AAAAATAGAGGGAAGAGTGATAACGTCCCCTCAGAAGAAGTTGTCAAGAAAATGAAAAATTATTGGCGGCAGCTGCTGAACGCCAAACTGATCACACAACGGAAGTTCGATAATCTGACTAAGGCTGAAC :  3867
p201N_cas9 : AAAAATAGAGGGAAGAGTGATAACGTCCCCTCAGAAGAAGTTGTCAAGAAAATGAAAAATTATTGGCGGCAGCTGCTGAACGCCAAACTGATCACACAACGGAAGTTCGATAATCTGACTAAGGCTGAAC : 12610
             AAAAATAGAGGGAAGAGTGATAACGTCCCCTCAGAAGAAGTTGTCAAGAAAATGAAAAATTATTGGCGGCAGCTGCTGAACGCCAAACTGATCACACAACGGAAGTTCGATAATCTGACTAAGGCTGAAC        
                                                                                                                                                       
                  12620         *     12640         *     12660         *     12680         *     12700         *     12720         *     12740        
NODE_10593 : GAGGTGGCCTGTCTGAGTTGGATAAAGCCGGCTTCATCAAAAGGCAGCTTGTTGAGACACGCCAGATCACCAAGCACGTGGCCCAAATTCTCGATTCACGCATGAACACCAAGTACGATGAAAATGACAA :  3997
p201N_cas9 : GAGGTGGCCTGTCTGAGTTGGATAAAGCCGGCTTCATCAAAAGGCAGCTTGTTGAGACACGCCAGATCACCAAGCACGTGGCCCAAATTCTCGATTCACGCATGAACACCAAGTACGATGAAAATGACAA : 12740
             GAGGTGGCCTGTCTGAGTTGGATAAAGCCGGCTTCATCAAAAGGCAGCTTGTTGAGACACGCCAGATCACCAAGCACGTGGCCCAAATTCTCGATTCACGCATGAACACCAAGTACGATGAAAATGACAA        
                                                                                                                                                       
                      *     12760         *     12780         *     12800         *     12820         *     12840         *     12860         *        
NODE_10593 : ACTGATTCGAGAGGTGAAAGTTATTACTCTGAAGTCTAAGCTGGTCTCAGATTTCAGAAAGGACTTTCAGTTTTATAAGGTGAGAGAGATCAACAATTACCACCATGCGCATGATGCCTACCTGAATGCA :  4127
p201N_cas9 : ACTGATTCGAGAGGTGAAAGTTATTACTCTGAAGTCTAAGCTGGTCTCAGATTTCAGAAAGGACTTTCAGTTTTATAAGGTGAGAGAGATCAACAATTACCACCATGCGCATGATGCCTACCTGAATGCA : 12870
             ACTGATTCGAGAGGTGAAAGTTATTACTCTGAAGTCTAAGCTGGTCTCAGATTTCAGAAAGGACTTTCAGTTTTATAAGGTGAGAGAGATCAACAATTACCACCATGCGCATGATGCCTACCTGAATGCA        
                                                                                                                                                       
                  12880         *     12900         *     12920         *     12940         *     12960         *     12980         *     13000        
NODE_10593 : GTGGTAGGCACTGCACTTATCAAAAAATATCCCAAGCTTGAATCTGAATTTGTTTACGGAGACTATAAAGTGTACGATGTTAGGAAAATGATCGCAAAGTCTGAGCAGGAAATAGGCAAGGCCACCGCTA :  4257
p201N_cas9 : GTGGTAGGCACTGCACTTATCAAAAAATATCCCAAGCTTGAATCTGAATTTGTTTACGGAGACTATAAAGTGTACGATGTTAGGAAAATGATCGCAAAGTCTGAGCAGGAAATAGGCAAGGCCACCGCTA : 13000
             GTGGTAGGCACTGCACTTATCAAAAAATATCCCAAGCTTGAATCTGAATTTGTTTACGGAGACTATAAAGTGTACGATGTTAGGAAAATGATCGCAAAGTCTGAGCAGGAAATAGGCAAGGCCACCGCTA        
                                                                                                                                                       
                      *     13020         *     13040         *     13060         *     13080         *     13100         *     13120         *        
NODE_10593 : AGTACTTCTTTTACAGCAATATTATGAATTTTTTCAAGACCGAGATTACACTGGCCAATGGAGAGATTCGGAAGCGACCACTTATCGAAACAAACGGAGAAACAGGAGAAATCGTGTGGGACAAGGGTAG :  4387
p201N_cas9 : AGTACTTCTTTTACAGCAATATTATGAATTTTTTCAAGACCGAGATTACACTGGCCAATGGAGAGATTCGGAAGCGACCACTTATCGAAACAAACGGAGAAACAGGAGAAATCGTGTGGGACAAGGGTAG : 13130
             AGTACTTCTTTTACAGCAATATTATGAATTTTTTCAAGACCGAGATTACACTGGCCAATGGAGAGATTCGGAAGCGACCACTTATCGAAACAAACGGAGAAACAGGAGAAATCGTGTGGGACAAGGGTAG        
                                                                                                                                                       
                  13140         *     13160         *     13180         *     13200         *     13220         *     13240         *     13260        
NODE_10593 : GGATTTCGCGACAGTCCGGAAGGTCCTGTCCATGCCGCAGGTGAACATCGTTAAAAAGACCGAAGTACAGACCGGAGGCTTCTCCAAGGAAAGTATCCTCCCGAAAAGGAACAGCGACAAGCTGATCGCA :  4517
p201N_cas9 : GGATTTCGCGACAGTCCGGAAGGTCCTGTCCATGCCGCAGGTGAACATCGTTAAAAAGACCGAAGTACAGACCGGAGGCTTCTCCAAGGAAAGTATCCTCCCGAAAAGGAACAGCGACAAGCTGATCGCA : 13260
             GGATTTCGCGACAGTCCGGAAGGTCCTGTCCATGCCGCAGGTGAACATCGTTAAAAAGACCGAAGTACAGACCGGAGGCTTCTCCAAGGAAAGTATCCTCCCGAAAAGGAACAGCGACAAGCTGATCGCA        
                                                                                                                                                       
                      *     13280         *     13300         *     13320         *     13340         *     13360         *     13380         *        
NODE_10593 : CGCAAAAAAGATTGGGACCCCAAGAAATACGGCGGATTCGATTCTCCTACAGTCGCTTACAGTGTACTGGTTGTGGCCAAAGTGGAGAAAGGGAAGTCTAAAAAACTCAAAAGCGTCAAGGAACTGCTGG :  4647
p201N_cas9 : CGCAAAAAAGATTGGGACCCCAAGAAATACGGCGGATTCGATTCTCCTACAGTCGCTTACAGTGTACTGGTTGTGGCCAAAGTGGAGAAAGGGAAGTCTAAAAAACTCAAAAGCGTCAAGGAACTGCTGG : 13390
             CGCAAAAAAGATTGGGACCCCAAGAAATACGGCGGATTCGATTCTCCTACAGTCGCTTACAGTGTACTGGTTGTGGCCAAAGTGGAGAAAGGGAAGTCTAAAAAACTCAAAAGCGTCAAGGAACTGCTGG        
                                                                                                                                                       
                  13400         *     13420         *     13440         *     13460         *     13480         *     13500         *     13520        
NODE_10593 : GCATCACAATCATGGAGCGATCAAGCTTCGAAAAAAACCCCATCGACTTTCTCGAGGCGAAAGGATATAAAGAGGTCAAAAAAGACCTCATCATTAAGCTTCCCAAGTACTCTCTCTTTGAGCTTGAAAA :  4777
p201N_cas9 : GCATCACAATCATGGAGCGATCAAGCTTCGAAAAAAACCCCATCGACTTTCTCGAGGCGAAAGGATATAAAGAGGTCAAAAAAGACCTCATCATTAAGCTTCCCAAGTACTCTCTCTTTGAGCTTGAAAA : 13520
             GCATCACAATCATGGAGCGATCAAGCTTCGAAAAAAACCCCATCGACTTTCTCGAGGCGAAAGGATATAAAGAGGTCAAAAAAGACCTCATCATTAAGCTTCCCAAGTACTCTCTCTTTGAGCTTGAAAA        
                                                                                                                                                       
                      *     13540         *     13560         *     13580         *     13600         *     13620         *     13640         *        
NODE_10593 : CGGCCGGAAACGAATGCTCGCTAGTGCGGGCGAGCTGCAGAAAGGTAACGAGCTGGCACTGCCCTCTAAATACGTTAATTTCTTGTATCTGGCCAGCCACTATGAAAAGCTCAAAGGGTCTCCCGAAGAT :  4907
p201N_cas9 : CGGCCGGAAACGAATGCTCGCTAGTGCGGGCGAGCTGCAGAAAGGTAACGAGCTGGCACTGCCCTCTAAATACGTTAATTTCTTGTATCTGGCCAGCCACTATGAAAAGCTCAAAGGGTCTCCCGAAGAT : 13650
             CGGCCGGAAACGAATGCTCGCTAGTGCGGGCGAGCTGCAGAAAGGTAACGAGCTGGCACTGCCCTCTAAATACGTTAATTTCTTGTATCTGGCCAGCCACTATGAAAAGCTCAAAGGGTCTCCCGAAGAT        
                                                                                                                                                       
                  13660         *     13680         *     13700         *     13720         *     13740         *     13760         *     13780        
NODE_10593 : AATGAGCAGAAGCAGCTGTTCGTGGAACAACACAAACACTACCTTGATGAGATCATCGAGCAAATAAGCGAATTCTCCAAAAGAGTGATCCTCGCCGACGCTAACCTCGATAAGGTGCTTTCTGCTTACA :  5037
p201N_cas9 : AATGAGCAGAAGCAGCTGTTCGTGGAACAACACAAACACTACCTTGATGAGATCATCGAGCAAATAAGCGAATTCTCCAAAAGAGTGATCCTCGCCGACGCTAACCTCGATAAGGTGCTTTCTGCTTACA : 13780
             AATGAGCAGAAGCAGCTGTTCGTGGAACAACACAAACACTACCTTGATGAGATCATCGAGCAAATAAGCGAATTCTCCAAAAGAGTGATCCTCGCCGACGCTAACCTCGATAAGGTGCTTTCTGCTTACA        
                                                                                                                                                       
                      *     13800         *     13820         *     13840         *     13860         *     13880         *     13900         *        
NODE_10593 : ATAAGCACAGGGATAAGCCCATCAGGGAGCAGGCAGAAAACATTATCCACTTGTTTACTCTGACCAACTTGGGCGCGCCTGCAGCCTTCAAGTACTTCGACACCACCATAGACAGAAAGCGGTACACCTC :  5167
p201N_cas9 : ATAAGCACAGGGATAAGCCCATCAGGGAGCAGGCAGAAAACATTATCCACTTGTTTACTCTGACCAACTTGGGCGCGCCTGCAGCCTTCAAGTACTTCGACACCACCATAGACAGAAAGCGGTACACCTC : 13910
             ATAAGCACAGGGATAAGCCCATCAGGGAGCAGGCAGAAAACATTATCCACTTGTTTACTCTGACCAACTTGGGCGCGCCTGCAGCCTTCAAGTACTTCGACACCACCATAGACAGAAAGCGGTACACCTC        
                                                                                                                                                       
                  13920         *     13940         *     13960         *     13980         *     14000         *     14020         *     14040        
NODE_10593 : TACAAAGGAGGTCCTGGACGCCACACTGATTCATCAGTCAATTACGGGGCTCTATGAAACAAGAATCGACCTCTCTCAGCTCGGTGGAGACAGCAGGGCTGACCCCAAGAAGAAGAGGAAGGTGTGACCG :  5297
p201N_cas9 : TACAAAGGAGGTCCTGGACGCCACACTGATTCATCAGTCAATTACGGGGCTCTATGAAACAAGAATCGACCTCTCTCAGCTCGGTGGAGACAGCAGGGCTGACCCCAAGAAGAAGAGGAAGGTGTGACCG : 14040
             TACAAAGGAGGTCCTGGACGCCACACTGATTCATCAGTCAATTACGGGGCTCTATGAAACAAGAATCGACCTCTCTCAGCTCGGTGGAGACAGCAGGGCTGACCCCAAGAAGAAGAGGAAGGTGTGACCG        
                                                                                                                                                       
                      *     14060         *     14080         *     14100         *     14120         *     14140         *     14160         *        
NODE_10593 : CGGATATCTGCAGAAGCTTACGCGTCGACGTCTCGAGGCCGGCCGAGCTCGAATTTCCCCGATCGTTCAAACATTTGGCAATAAAGTTTCTTAAGATTGAATCCTGTTGCCGGTCTTGCGATGATTATCA :  5427
p201N_cas9 : CGGATATCTGCAGAAGCTTACGCGTCGACGTCTCGAGGCCGGCCGAGCTCGAATTTCCCCGATCGTTCAAACATTTGGCAATAAAGTTTCTTAAGATTGAATCCTGTTGCCGGTCTTGCGATGATTATCA : 14170
             CGGATATCTGCAGAAGCTTACGCGTCGACGTCTCGAGGCCGGCCGAGCTCGAATTTCCCCGATCGTTCAAACATTTGGCAATAAAGTTTCTTAAGATTGAATCCTGTTGCCGGTCTTGCGATGATTATCA        
                                                                                                                                                       
                  14180         *     14200         *     14220         *     14240         *     14260         *     14280         *     14300        
NODE_10593 : TATAATTTCTGTTGAATTACGTTAAGCATGTAATAATTAACATGTAATGCATGACGTTATTTATGAGATGGGTTTTTATGATTAGAGTCCCGCAATTATACATTTAATACGCGATAGAAAACAAAATATA :  5557
p201N_cas9 : TATAATTTCTGTTGAATTACGTTAAGCATGTAATAATTAACATGTAATGCATGACGTTATTTATGAGATGGGTTTTTATGATTAGAGTCCCGCAATTATACATTTAATACGCGATAGAAAACAAAATATA : 14300
             TATAATTTCTGTTGAATTACGTTAAGCATGTAATAATTAACATGTAATGCATGACGTTATTTATGAGATGGGTTTTTATGATTAGAGTCCCGCAATTATACATTTAATACGCGATAGAAAACAAAATATA        
                                                                                                                                                       
                      *     14320         *     14340         *     14360         *     14380         *     14400         *     14420         *        
NODE_10593 : GCGCGCAAACTAGGATAAATTATCGCGCGCGGTGTCATCTATGTTACTAGATCGGGAATTGATATC-TAGGGATAACAGGGTAATAAGCTTGGCACTGGCCGTCGTTTTACAACGTCGTGACTGGGAAAA :  5686
p201N_cas9 : GCGCGCAAACTAGGATAAATTATCGCGCGCGGTGTCATCTATGTTACTAGATCGGGAATTGATATCGTAGGGATAACAGGGTAATAAGCTTGGCACTGGCCGTCGTTTTACAACGTCGTGACTGGGAAAA : 14430
             GCGCGCAAACTAGGATAAATTATCGCGCGCGGTGTCATCTATGTTACTAGATCGGGAATTGATATC TAGGGATAACAGGGTAATAAGCTTGGCACTGGCCGTCGTTTTACAACGTCGTGACTGGGAAAA        
                                                                                                                                                       
                  14440         *     14460         *     14480         *     14500         *     14520         *     14540         *     14560        
NODE_10593 : CCCTGGCGTTACCCAACTTAATCGCCTTGCAGCACATCCCCCTTTCGCCAGCTGGCGTAATAGCGAAGAGGCCCGCACCGATCGCCCTTCCCAACAGTTGCGCAGCCTGAATGGCGAATGAGCTTGAGCT :  5816
p201N_cas9 : CCCTGGCGTTACCCAACTTAATCGCCTTGCAGCACATCCCCCTTTCGCCAGCTGGCGTAATAGCGAAGAGGCCCGCACCGATCGCCCTTCCCAACAGTTGCGCAGCCTGAATGGCGAATGAGCTTGAGCT : 14560
             CCCTGGCGTTACCCAACTTAATCGCCTTGCAGCACATCCCCCTTTCGCCAGCTGGCGTAATAGCGAAGAGGCCCGCACCGATCGCCCTTCCCAACAGTTGCGCAGCCTGAATGGCGAATGAGCTTGAGCT        
                                                                                                                                                       
                      *     14580         *     14600         *     14620         *     14640         *     14660         *     14680         *        
NODE_10593 : TGGATCAGATTGTCGTTTCCCGCCTTCAGTTTAAACTATCAGTTGCGTATTGGCTAGAGCAATTCGGCGTTAATTCAGTACATTAAAAACGTCCGC-AATGTGTTATTAAGTTGTCTAAGCGTCAATTTG :  5945
p201N_cas9 : TGGATCAGATTGTCGTTTCCCGCCTTCAGTTTAAACTATCAGT---GT-TTGACAGGATATATTGGCGGGTAAACCTAAGAGAAAAGAGCGTTTATTAGAATAACGGATATTTAAAAGGGCGTGAAAAGG : 14686
             TGGATCAGATTGTCGTTTCCCGCCTTCAGTTTAAACTATCAGT   GT TTG C  GA   ATT G  G TAA  C        AA A CGT     A   T       A TT      GCGT AA   G        
                                                                                                   
                  14700         *     14720         *     14740         *     14760                
NODE_10593 : TTTAACCTTCTTCATTAGTATTGTGTGTTGCTATCTCGCCTCTTCAATCGCATTTCCACCGACGGGATCAATCTTTGG :  6023
p201N_cas9 : TTTATCCGT--TCGTCCAT-TTGTATGTGCATGCC----------AACCACAGGGTTCCCCTCGGGATCAA------- : 14744
             TTTA CC T  TC T   T TTGT TGT   T  C          AA C CA      CC  CGGGATCAA               
