# Supplementary material for: CRISPR/Cas9-mediated targeted mutagenesis of TAS4 and MYBA7 loci in grapevine rootstock 101-14
Source: Transgenic Res. 2020 Apr 23;29(3):355–67. doi: 10.1007/s11248-020-00196-w (PMC7283210; doi:10.1007/s11248-020-00196-w)
Supplement: Supplementary file 2 — Supplementary material 2 (RTF 288 kb) [file 11248_2020_196_MOESM2_ESM.rtf]

Suppl. docx2.  Alignment of SPAdes contig output to p201N-cas9 vector sequence to map the T-DNA integration loci of MYBA7-6 transgenic plant


>chr15 chr15:12878392..12878434 (- strand) class=match length=43
ATCCCTCTAGTACTTTATTACTTTTTTTGTAATTCACTTTTAA                                                                                                                   
Immediate Downstream of RB                        
 382 bp downstream of LB     20         *        40         *        60         *        80         *       100         *       120         *        
NODE_6452_ : AATATTTTAAACAATAATATATTTTTATAATAATAAAAATATTTAAAATATATGTTTTATAAAATTAATTACTTTTATGTACTAAATTCAAGATATAAAACTAATTATAAATTATTACAAATATATTATT :   130
p201N_cas9 : ---------------------------------------------------------------------------------------------------------------------------------- :     -
                                                                                                                                                       
                                                                                                                                                       
                    140         *       160         *       180         *       200         *       220         *       240         *       260        
NODE_6452_ : AATTTTGTATATTAAATTCAATATATTATTATTTAGAAATGATATATTTTTTTTTTAGAGAAAATTAAAATTGTCTCTTCAATGGATGATTTTAATTTTAAGATTTTTTTTTTAAAAAGAGATCATCTCT :   260
p201N_cas9 : ---------------------------------------------------------------------------------------------------------------------------------- :     -
                                                                                                                                                       
                                                                                                                                                       
                      *       280         *       300         *       320         *       340         *       360         *       380         *        
NODE_6452_ : TTAAAACTTTTTTTTTTGAAAATTTTTAGAAATCTTTTGTTCAATTTTTGGACTTTTTTTTTTTTTTTTTGAAGAATTTTTAAAAATCTTCTCTTAAAGAGGCAATTTTCCGATACCTTTTAATTTTTTT :   390
p201N_cas9 : ---------------------------------------------------------------------------------------------------------------------------------- :     -
                                                                                                                                                       
                                                                                                                                                       
                    400         *       420         *       440         *       460         *       480         *       500         *       520        
NODE_6452_ : ACAAATTATTATTTTTTAATCATCTATAATAAAACTTGAAAGTAACCATGTTTGCAAAATAGAGGATTATTTTTATACCCCCATCCTCACTATCAAGTACACAAATGTTAAATGTGTACTGACATTTAAT :   520
p201N_cas9 : -----------------------------TTGATCCCGAGGGGAACCCTGTGGTTGGCATGCACATACAAATGGACGAACGGATAAACCTTTTCACGCCC-----TTTTAAATATCCGTTA--TTCTAAT :    94
                                          T  A C  GA  G AACC TGT       AT  A     A  T  A    C  AT   C  T TCA G  C     T TTAAAT T    T    T TAAT        
                                                                                                                                                       
                      *       540         *       560         *       580         *       600         *       620         *       640         *        
NODE_6452_ : ATCCCTCTAGTACTTTATTACTTTTTTTGTAATTCACTTTTAAATCAAACACTGATAGTTTAAACTGAAGGCGGGAAACGACAATCTGATCCAAGCTCAAGCTCATTCGCCATTCAGGCTGCGCAACTGT :   650
p201N_cas9 : AAACGCTCTTTTCTCTTAGGTTTACCCGCCAATATATCCT---GTCAAACACTGATAGTTTAAACTGAAGGCGGGAAACGACAATCTGATCCAAGCTCAAGCTCATTCGCCATTCAGGCTGCGCAACTGT :   221
             A  C      T CT T     TT       AAT  A   T    TCAAACACTGATAGTTTAAACTGAAGGCGGGAAACGACAATCTGATCCAAGCTCAAGCTCATTCGCCATTCAGGCTGCGCAACTGT        
                                                                                                                                                       
                    660         *       680         *       700         *       720         *       740         *       760         *       780        
NODE_6452_ : TGGGAAGGGCGATCGGTGCGGGCCTCTTCGCTATTACGCCAGCTGGCGAAAGGGGGATGTGCTGCAAGGCGATTAAGTTGGGTAACGCCAGGGTTTTCCCAGTCACGACGTTGTAAAACGACGGCCAGTG :   780
p201N_cas9 : TGGGAAGGGCGATCGGTGCGGGCCTCTTCGCTATTACGCCAGCTGGCGAAAGGGGGATGTGCTGCAAGGCGATTAAGTTGGGTAACGCCAGGGTTTTCCCAGTCACGACGTTGTAAAACGACGGCCAGTG :   351
             TGGGAAGGGCGATCGGTGCGGGCCTCTTCGCTATTACGCCAGCTGGCGAAAGGGGGATGTGCTGCAAGGCGATTAAGTTGGGTAACGCCAGGGTTTTCCCAGTCACGACGTTGTAAAACGACGGCCAGTG        
                                                                                                                                                       
                      *       800         *       820         *       840         *       860         *       880         *       900         *        
NODE_6452_ : CCAAGCTTATTACCCTGTTATCCCTA-GATATCAATTCCCGATCTAGTAACATAGATGACACCGCGCGCGATAATTTATCCTAGTTTGCGCGCTATATTTTGTTTTCTATCGCGTATTAAATGTATAATT :   909
p201N_cas9 : CCAAGCTTATTACCCTGTTATCCCTACGATATCAATTCCCGATCTAGTAACATAGATGACACCGCGCGCGATAATTTATCCTAGTTTGCGCGCTATATTTTGTTTTCTATCGCGTATTAAATGTATAATT :   481
             CCAAGCTTATTACCCTGTTATCCCTA GATATCAATTCCCGATCTAGTAACATAGATGACACCGCGCGCGATAATTTATCCTAGTTTGCGCGCTATATTTTGTTTTCTATCGCGTATTAAATGTATAATT        
                                                                                                                                                       
                    920         *       940         *       960         *       980         *      1000         *      1020         *      1040        
NODE_6452_ : GCGGGACTCTAATCATAAAAACCCATCTCATAAATAACGTCATGCATTACATGTTAATTATTACATGCTTAACGTAATTCAACAGAAATTATATGATAATCATCGCAAGACCGGCAACAGGATTCAATCT :  1039
p201N_cas9 : GCGGGACTCTAATCATAAAAACCCATCTCATAAATAACGTCATGCATTACATGTTAATTATTACATGCTTAACGTAATTCAACAGAAATTATATGATAATCATCGCAAGACCGGCAACAGGATTCAATCT :   611
             GCGGGACTCTAATCATAAAAACCCATCTCATAAATAACGTCATGCATTACATGTTAATTATTACATGCTTAACGTAATTCAACAGAAATTATATGATAATCATCGCAAGACCGGCAACAGGATTCAATCT        
                                                                                                                                                       
                      *      1060         *      1080         *      1100         *      1120         *      1140         *      1160         *        
NODE_6452_ : TAAGAAACTTTATTGCCAAATGTTTGAACGATCGGGGAAATTCGAGCTCGGCCGGCCTCGAGACGTCGACGCGTAAGCTTCTGCAGATATCCGCGGTCACACCTTCCTCTTCTTCTTGGGGTCAGCCCTG :  1169
p201N_cas9 : TAAGAAACTTTATTGCCAAATGTTTGAACGATCGGGGAAATTCGAGCTCGGCCGGCCTCGAGACGTCGACGCGTAAGCTTCTGCAGATATCCGCGGTCACACCTTCCTCTTCTTCTTGGGGTCAGCCCTG :   741
             TAAGAAACTTTATTGCCAAATGTTTGAACGATCGGGGAAATTCGAGCTCGGCCGGCCTCGAGACGTCGACGCGTAAGCTTCTGCAGATATCCGCGGTCACACCTTCCTCTTCTTCTTGGGGTCAGCCCTG        
                                                                                                                                                       
                   1180         *      1200         *      1220         *      1240         *      1260         *      1280         *      1300        
NODE_6452_ : CTGTCTCCACCGAGCTGAGAGAGGTCGATTCTTGTTTCATAGAGCCCCGTAATTGACTGATGAATCAGTGTGGCGTCCAGGACCTCCTTTGTAGAGGTGTACCGCTTTCTGTCTATGGTGGTGTCGAAGT :  1299
p201N_cas9 : CTGTCTCCACCGAGCTGAGAGAGGTCGATTCTTGTTTCATAGAGCCCCGTAATTGACTGATGAATCAGTGTGGCGTCCAGGACCTCCTTTGTAGAGGTGTACCGCTTTCTGTCTATGGTGGTGTCGAAGT :   871
             CTGTCTCCACCGAGCTGAGAGAGGTCGATTCTTGTTTCATAGAGCCCCGTAATTGACTGATGAATCAGTGTGGCGTCCAGGACCTCCTTTGTAGAGGTGTACCGCTTTCTGTCTATGGTGGTGTCGAAGT        
                                                                                                                                                       
                      *      1320         *      1340         *      1360         *      1380         *      1400         *      1420         *        
NODE_6452_ : ACTTGAAGGCTGCAGGCGCGCCCAAGTTGGTCAGAGTAAACAAGTGGATAATGTTTTCTGCCTGCTCCCTGATGGGCTTATCCCTGTGCTTATTGTAAGCAGAAAGCACCTTATCGAGGTTAGCGTCGGC :  1429
p201N_cas9 : ACTTGAAGGCTGCAGGCGCGCCCAAGTTGGTCAGAGTAAACAAGTGGATAATGTTTTCTGCCTGCTCCCTGATGGGCTTATCCCTGTGCTTATTGTAAGCAGAAAGCACCTTATCGAGGTTAGCGTCGGC :  1001
             ACTTGAAGGCTGCAGGCGCGCCCAAGTTGGTCAGAGTAAACAAGTGGATAATGTTTTCTGCCTGCTCCCTGATGGGCTTATCCCTGTGCTTATTGTAAGCAGAAAGCACCTTATCGAGGTTAGCGTCGGC        
                                                                                                                                                       
                   1440         *      1460         *      1480         *      1500         *      1520         *      1540         *      1560        
NODE_6452_ : GAGGATCACTCTTTTGGAGAATTCGCTTATTTGCTCGATGATCTCATCAAGGTAGTGTTTGTGTTGTTCCACGAACAGCTGCTTCTGCTCATTATCTTCGGGAGACCCTTTGAGCTTTTCATAGTGGCTG :  1559
p201N_cas9 : GAGGATCACTCTTTTGGAGAATTCGCTTATTTGCTCGATGATCTCATCAAGGTAGTGTTTGTGTTGTTCCACGAACAGCTGCTTCTGCTCATTATCTTCGGGAGACCCTTTGAGCTTTTCATAGTGGCTG :  1131
             GAGGATCACTCTTTTGGAGAATTCGCTTATTTGCTCGATGATCTCATCAAGGTAGTGTTTGTGTTGTTCCACGAACAGCTGCTTCTGCTCATTATCTTCGGGAGACCCTTTGAGCTTTTCATAGTGGCTG        
                                                                                                                                                       
                      *      1580         *      1600         *      1620         *      1640         *      1660         *      1680         *        
NODE_6452_ : GCCAGATACAAGAAATTAACGTATTTAGAGGGCAGTGCCAGCTCGTTACCTTTCTGCAGCTCGCCCGCACTAGCGAGCATTCGTTTCCGGCCGTTTTCAAGCTCAAAGAGAGAGTACTTGGGAAGCTTAA :  1689
p201N_cas9 : GCCAGATACAAGAAATTAACGTATTTAGAGGGCAGTGCCAGCTCGTTACCTTTCTGCAGCTCGCCCGCACTAGCGAGCATTCGTTTCCGGCCGTTTTCAAGCTCAAAGAGAGAGTACTTGGGAAGCTTAA :  1261
             GCCAGATACAAGAAATTAACGTATTTAGAGGGCAGTGCCAGCTCGTTACCTTTCTGCAGCTCGCCCGCACTAGCGAGCATTCGTTTCCGGCCGTTTTCAAGCTCAAAGAGAGAGTACTTGGGAAGCTTAA        
                                                                                                                                                       
                   1700         *      1720         *      1740         *      1760         *      1780         *      1800         *      1820        
NODE_6452_ : TGATGAGGTCTTTTTTGACCTCTTTATATCCTTTCGCCTCGAGAAAGTCGATGGGGTTTTTTTCGAAGCTTGATCGCTCCATGATTGTGATGCCCAGCAGTTCCTTGACGCTTTTGAGTTTTTTAGACTT :  1819
p201N_cas9 : TGATGAGGTCTTTTTTGACCTCTTTATATCCTTTCGCCTCGAGAAAGTCGATGGGGTTTTTTTCGAAGCTTGATCGCTCCATGATTGTGATGCCCAGCAGTTCCTTGACGCTTTTGAGTTTTTTAGACTT :  1391
             TGATGAGGTCTTTTTTGACCTCTTTATATCCTTTCGCCTCGAGAAAGTCGATGGGGTTTTTTTCGAAGCTTGATCGCTCCATGATTGTGATGCCCAGCAGTTCCTTGACGCTTTTGAGTTTTTTAGACTT        
                                                                                                                                                       
                      *      1840         *      1860         *      1880         *      1900         *      1920         *      1940         *        
NODE_6452_ : CCCTTTCTCCACTTTGGCCACAACCAGTACACTGTAAGCGACTGTAGGAGAATCGAATCCGCCGTATTTCTTGGGGTCCCAATCTTTTTTGCGTGCGATCAGCTTGTCGCTGTTCCTTTTCGGGAGGATA :  1949
p201N_cas9 : CCCTTTCTCCACTTTGGCCACAACCAGTACACTGTAAGCGACTGTAGGAGAATCGAATCCGCCGTATTTCTTGGGGTCCCAATCTTTTTTGCGTGCGATCAGCTTGTCGCTGTTCCTTTTCGGGAGGATA :  1521
             CCCTTTCTCCACTTTGGCCACAACCAGTACACTGTAAGCGACTGTAGGAGAATCGAATCCGCCGTATTTCTTGGGGTCCCAATCTTTTTTGCGTGCGATCAGCTTGTCGCTGTTCCTTTTCGGGAGGATA        
                                                                                                                                                       
                   1960         *      1980         *      2000         *      2020         *      2040         *      2060         *      2080        
NODE_6452_ : CTTTCCTTGGAGAAGCCTCCGGTCTGTACTTCGGTCTTTTTAACGATGTTCACCTGCGGCATGGACAGGACCTTCCGGACTGTCGCGAAATCCCTACCCTTGTCCCACACGATTTCTCCTGTTTCTCCGT :  2079
p201N_cas9 : CTTTCCTTGGAGAAGCCTCCGGTCTGTACTTCGGTCTTTTTAACGATGTTCACCTGCGGCATGGACAGGACCTTCCGGACTGTCGCGAAATCCCTACCCTTGTCCCACACGATTTCTCCTGTTTCTCCGT :  1651
             CTTTCCTTGGAGAAGCCTCCGGTCTGTACTTCGGTCTTTTTAACGATGTTCACCTGCGGCATGGACAGGACCTTCCGGACTGTCGCGAAATCCCTACCCTTGTCCCACACGATTTCTCCTGTTTCTCCGT        
                                                                                                                                                       
                      *      2100         *      2120         *      2140         *      2160         *      2180         *      2200         *        
NODE_6452_ : TTGTTTCGATAAGTGGTCGCTTCCGAATCTCTCCATTGGCCAGTGTAATCTCGGTCTTGAAAAAATTCATAATATTGCTGTAAAAGAAGTACTTAGCGGTGGCCTTGCCTATTTCCTGCTCAGACTTTGC :  2209
p201N_cas9 : TTGTTTCGATAAGTGGTCGCTTCCGAATCTCTCCATTGGCCAGTGTAATCTCGGTCTTGAAAAAATTCATAATATTGCTGTAAAAGAAGTACTTAGCGGTGGCCTTGCCTATTTCCTGCTCAGACTTTGC :  1781
             TTGTTTCGATAAGTGGTCGCTTCCGAATCTCTCCATTGGCCAGTGTAATCTCGGTCTTGAAAAAATTCATAATATTGCTGTAAAAGAAGTACTTAGCGGTGGCCTTGCCTATTTCCTGCTCAGACTTTGC        
                                                                                                                                                       
                   2220         *      2240         *      2260         *      2280         *      2300         *      2320         *      2340        
NODE_6452_ : GATCATTTTCCTAACATCGTACACTTTATAGTCTCCGTAAACAAATTCAGATTCAAGCTTGGGATATTTTTTGATAAGTGCAGTGCCTACCACTGCATTCAGGTAGGCATCATGCGCATGGTGGTAATTG :  2339
p201N_cas9 : GATCATTTTCCTAACATCGTACACTTTATAGTCTCCGTAAACAAATTCAGATTCAAGCTTGGGATATTTTTTGATAAGTGCAGTGCCTACCACTGCATTCAGGTAGGCATCATGCGCATGGTGGTAATTG :  1911
             GATCATTTTCCTAACATCGTACACTTTATAGTCTCCGTAAACAAATTCAGATTCAAGCTTGGGATATTTTTTGATAAGTGCAGTGCCTACCACTGCATTCAGGTAGGCATCATGCGCATGGTGGTAATTG        
                                                                                                                                                       
                      *      2360         *      2380         *      2400         *      2420         *      2440         *      2460         *        
NODE_6452_ : TTGATCTCTCTCACCTTATAAAACTGAAAGTCCTTTCTGAAATCTGAGACCAGCTTAGACTTCAGAGTAATAACTTTCACCTCTCGAATCAGTTTGTCATTTTCATCGTACTTGGTGTTCATGCGTGAAT :  2469
p201N_cas9 : TTGATCTCTCTCACCTTATAAAACTGAAAGTCCTTTCTGAAATCTGAGACCAGCTTAGACTTCAGAGTAATAACTTTCACCTCTCGAATCAGTTTGTCATTTTCATCGTACTTGGTGTTCATGCGTGAAT :  2041
             TTGATCTCTCTCACCTTATAAAACTGAAAGTCCTTTCTGAAATCTGAGACCAGCTTAGACTTCAGAGTAATAACTTTCACCTCTCGAATCAGTTTGTCATTTTCATCGTACTTGGTGTTCATGCGTGAAT        
                                                                                                                                                       
                   2480         *      2500         *      2520         *      2540         *      2560         *      2580         *      2600        
NODE_6452_ : CGAGAATTTGGGCCACGTGCTTGGTGATCTGGCGTGTCTCAACAAGCTGCCTTTTGATGAAGCCGGCTTTATCCAACTCAGACAGGCCACCTCGTTCAGCCTTAGTCAGATTATCGAACTTCCGTTGTGT :  2599
p201N_cas9 : CGAGAATTTGGGCCACGTGCTTGGTGATCTGGCGTGTCTCAACAAGCTGCCTTTTGATGAAGCCGGCTTTATCCAACTCAGACAGGCCACCTCGTTCAGCCTTAGTCAGATTATCGAACTTCCGTTGTGT :  2171
             CGAGAATTTGGGCCACGTGCTTGGTGATCTGGCGTGTCTCAACAAGCTGCCTTTTGATGAAGCCGGCTTTATCCAACTCAGACAGGCCACCTCGTTCAGCCTTAGTCAGATTATCGAACTTCCGTTGTGT        
                                                                                                                                                       
                      *      2620         *      2640         *      2660         *      2680         *      2700         *      2720         *        
NODE_6452_ : GATCAGTTTGGCGTTCAGCAGCTGCCGCCAATAATTTTTCATTTTCTTGACAACTTCTTCTGAGGGGACGTTATCACTCTTCCCTCTATTTTTATCGGATCTTGTCAACACTTTATTATCAATAGAATCA :  2729
p201N_cas9 : GATCAGTTTGGCGTTCAGCAGCTGCCGCCAATAATTTTTCATTTTCTTGACAACTTCTTCTGAGGGGACGTTATCACTCTTCCCTCTATTTTTATCGGATCTTGTCAACACTTTATTATCAATAGAATCA :  2301
             GATCAGTTTGGCGTTCAGCAGCTGCCGCCAATAATTTTTCATTTTCTTGACAACTTCTTCTGAGGGGACGTTATCACTCTTCCCTCTATTTTTATCGGATCTTGTCAACACTTTATTATCAATAGAATCA        
                                                                                                                                                       
                   2740         *      2760         *      2780         *      2800         *      2820         *      2840         *      2860        
NODE_6452_ : TCTTTGAGAAAAGACTGGGGCACGATATGATCCACGTCGTAGTCGGAGAGCCGATTGATGTCCAGTTCCTGATCCACGTACATGTCCCTGCCGTTCTGCAGGTAGTACAGGTAGAGCTTCTCATTCTGAA :  2859
p201N_cas9 : TCTTTGAGAAAAGACTGGGGCACGATATGATCCACGTCGTAGTCGGAGAGCCGATTGATGTCCAGTTCCTGATCCACGTACATGTCCCTGCCGTTCTGCAGGTAGTACAGGTAGAGCTTCTCATTCTGAA :  2431
             TCTTTGAGAAAAGACTGGGGCACGATATGATCCACGTCGTAGTCGGAGAGCCGATTGATGTCCAGTTCCTGATCCACGTACATGTCCCTGCCGTTCTGCAGGTAGTACAGGTAGAGCTTCTCATTCTGAA        
                                                                                                                                                       
                      *      2880         *      2900         *      2920         *      2940         *      2960         *      2980         *        
NODE_6452_ : GCTGGGTGTTTTCAACTGGGTGTTCCTTAAGGATTTGGGACCCCAGTTCTTTTATACCCTCTTCAATCCTCTTCATCCTTTCCCTACTGTTCTTCTGTCCCTTCTGGGTAGTTTGGTTCTCTCGGGCCAT :  2989
p201N_cas9 : GCTGGGTGTTTTCAACTGGGTGTTCCTTAAGGATTTGGGACCCCAGTTCTTTTATACCCTCTTCAATCCTCTTCATCCTTTCCCTACTGTTCTTCTGTCCCTTCTGGGTAGTTTGGTTCTCTCGGGCCAT :  2561
             GCTGGGTGTTTTCAACTGGGTGTTCCTTAAGGATTTGGGACCCCAGTTCTTTTATACCCTCTTCAATCCTCTTCATCCTTTCCCTACTGTTCTTCTGTCCCTTCTGGGTAGTTTGGTTCTCTCGGGCCAT        
                                                                                                                                                       
                   3000         *      3020         *      3040         *      3060         *      3080         *      3100         *      3120        
NODE_6452_ : CTCGATAACGATATTCTCGGGCTTATGCCTTCCCATTACTTTGACGAGTTCATCCACGACCTTAACGGTCTGCAGTATTCCCTTTTTGATAGCTGGGCTACCTGCAAGATTAGCGATGTGCTCGTGAAGA :  3119
p201N_cas9 : CTCGATAACGATATTCTCGGGCTTATGCCTTCCCATTACTTTGACGAGTTCATCCACGACCTTAACGGTCTGCAGTATTCCCTTTTTGATAGCTGGGCTACCTGCAAGATTAGCGATGTGCTCGTGAAGA :  2691
             CTCGATAACGATATTCTCGGGCTTATGCCTTCCCATTACTTTGACGAGTTCATCCACGACCTTAACGGTCTGCAGTATTCCCTTTTTGATAGCTGGGCTACCTGCAAGATTAGCGATGTGCTCGTGAAGA        
                                                                                                                                                       
                      *      3140         *      3160         *      3180         *      3200         *      3220         *      3240         *        
NODE_6452_ : CTGTCCCCCTGGCCAGAAACTTGTGCTTTCTGGATGTCCTCCTTAAAGGTGAGAGAGTCATCATGGATCAACTGCATGAAGTTCCGGTTGGCAAATCCATCGGACTTAAGAAAATCCAGGATTGTCTTTC :  3249
p201N_cas9 : CTGTCCCCCTGGCCAGAAACTTGTGCTTTCTGGATGTCCTCCTTAAAGGTGAGAGAGTCATCATGGATCAACTGCATGAAGTTCCGGTTGGCAAATCCATCGGACTTAAGAAAATCCAGGATTGTCTTTC :  2821
             CTGTCCCCCTGGCCAGAAACTTGTGCTTTCTGGATGTCCTCCTTAAAGGTGAGAGAGTCATCATGGATCAACTGCATGAAGTTCCGGTTGGCAAATCCATCGGACTTAAGAAAATCCAGGATTGTCTTTC        
                                                                                                                                                       
                   3260         *      3280         *      3300         *      3320         *      3340         *      3360         *      3380        
NODE_6452_ : CACTCTGCTTGTCTCGGATCCCATTGATCAGTTTTCTTGACAGCCGCCCCCATCCTGTATATCGGCGCCTCTTGAGCTGTTTCATGACTTTGTCGTCGAAGAGATGAGCGTAAGTTTTCAAGCGTTCTTC :  3379
p201N_cas9 : CACTCTGCTTGTCTCGGATCCCATTGATCAGTTTTCTTGACAGCCGCCCCCATCCTGTATATCGGCGCCTCTTGAGCTGTTTCATGACTTTGTCGTCGAAGAGATGAGCGTAAGTTTTCAAGCGTTCTTC :  2951
             CACTCTGCTTGTCTCGGATCCCATTGATCAGTTTTCTTGACAGCCGCCCCCATCCTGTATATCGGCGCCTCTTGAGCTGTTTCATGACTTTGTCGTCGAAGAGATGAGCGTAAGTTTTCAAGCGTTCTTC        
                                                                                                                                                       
                      *      3400         *      3420         *      3440         *      3460         *      3480         *      3500         *        
NODE_6452_ : AATCATCTCCCTATCTTCAAACAACGTAAGGGTGAGGACAATGTCCTCAAGAATGTCCTCGTTCTCCTCATTGTCCAGGAAGTCCTTGTCTTTAATGATTTTCAGGAGATCGTGATACGTTCCCAGGGAT :  3509
p201N_cas9 : AATCATCTCCCTATCTTCAAACAACGTAAGGGTGAGGACAATGTCCTCAAGAATGTCCTCGTTCTCCTCATTGTCCAGGAAGTCCTTGTCTTTAATGATTTTCAGGAGATCGTGATACGTTCCCAGGGAT :  3081
             AATCATCTCCCTATCTTCAAACAACGTAAGGGTGAGGACAATGTCCTCAAGAATGTCCTCGTTCTCCTCATTGTCCAGGAAGTCCTTGTCTTTAATGATTTTCAGGAGATCGTGATACGTTCCCAGGGAT        
                                                                                                                                                       
                   3520         *      3540         *      3560         *      3580         *      3600         *      3620         *      3640        
NODE_6452_ : GCGTTGAAGCGATCCTCCACTCCGCTGATTTCAACAGAGTCGAAACATTCAATCTTTTTGAAATAGTCTTCTTTGAGCTGTTTCACGGTAACTTTCCGGTTCGTCTTGAAGAGGAGGTCCACGATAGCTT :  3639
p201N_cas9 : GCGTTGAAGCGATCCTCCACTCCGCTGATTTCAACAGAGTCGAAACATTCAATCTTTTTGAAATAGTCTTCTTTGAGCTGTTTCACGGTAACTTTCCGGTTCGTCTTGAAGAGGAGGTCCACGATAGCTT :  3211
             GCGTTGAAGCGATCCTCCACTCCGCTGATTTCAACAGAGTCGAAACATTCAATCTTTTTGAAATAGTCTTCTTTGAGCTGTTTCACGGTAACTTTCCGGTTCGTCTTGAAGAGGAGGTCCACGATAGCTT        
                                                                                                                                                       
                      *      3660         *      3680         *      3700         *      3720         *      3740         *      3760         *        
NODE_6452_ : TCTTCTGCTCTCCAGACAGGAATGCTGGCTTTCTCATCCCTTCTGTGACGTATTTGACCTTGGTGAGCTCGTTATAAACTGTGAAGTACTCGTACAGCAGAGAGTGTTTAGGAAGCACCTTTTCGTTAGG :  3769
p201N_cas9 : TCTTCTGCTCTCCAGACAGGAATGCTGGCTTTCTCATCCCTTCTGTGACGTATTTGACCTTGGTGAGCTCGTTATAAACTGTGAAGTACTCGTACAGCAGAGAGTGTTTAGGAAGCACCTTTTCGTTAGG :  3341
             TCTTCTGCTCTCCAGACAGGAATGCTGGCTTTCTCATCCCTTCTGTGACGTATTTGACCTTGGTGAGCTCGTTATAAACTGTGAAGTACTCGTACAGCAGAGAGTGTTTAGGAAGCACCTTTTCGTTAGG        
                                                                                                                                                       
                   3780         *      3800         *      3820         *      3840         *      3860         *      3880         *      3900        
NODE_6452_ : CAGATTTTTATCAAAGTTAGTCATCCTTTCGATGAAGGACTGGGCAGAGGCCCCCTTATCCACGACTTCCTCGAAGTTCCAGGGAGTGATGGTCTCTTCTGATTTGCGAGTCATCCACGCGAATCTGGAA :  3899
p201N_cas9 : CAGATTTTTATCAAAGTTAGTCATCCTTTCGATGAAGGACTGGGCAGAGGCCCCCTTATCCACGACTTCCTCGAAGTTCCAGGGAGTGATGGTCTCTTCTGATTTGCGAGTCATCCACGCGAATCTGGAA :  3471
             CAGATTTTTATCAAAGTTAGTCATCCTTTCGATGAAGGACTGGGCAGAGGCCCCCTTATCCACGACTTCCTCGAAGTTCCAGGGAGTGATGGTCTCTTCTGATTTGCGAGTCATCCACGCGAATCTGGAA        
                                                                                                                                                       
                      *      3920         *      3940         *      3960         *      3980         *      4000         *      4020         *        
NODE_6452_ : TTTCCCCGGGCGAGGGGGCCTACATAGTAGGGTATCCGAAATGTGAGGATTTTCTCAATCTTTTCCCTGTTATCTTTCAAAAAGGGGTAGAAATCCTCTTGCCGCCTGAGGATAGCGTGCAGTTCGCCCA :  4029
p201N_cas9 : TTTCCCCGGGCGAGGGGGCCTACATAGTAGGGTATCCGAAATGTGAGGATTTTCTCAATCTTTTCCCTGTTATCTTTCAAAAAGGGGTAGAAATCCTCTTGCCGCCTGAGGATAGCGTGCAGTTCGCCCA :  3601
             TTTCCCCGGGCGAGGGGGCCTACATAGTAGGGTATCCGAAATGTGAGGATTTTCTCAATCTTTTCCCTGTTATCTTTCAAAAAGGGGTAGAAATCCTCTTGCCGCCTGAGGATAGCGTGCAGTTCGCCCA        
                                                                                                                                                       
                   4040         *      4060         *      4080         *      4100         *      4120         *      4140         *      4160        
NODE_6452_ : GGTGAATCTGGTGGGGGATGCTTCCATTGTCGAAAGTGCGCTGTTTGCGCAACAGATCTTCTCTGTTAAGCTTTACCAGCAGCTCCTCGGTGCCGTCCATTTTTTCCAAGATGGGCTTAATAAATTTGTA :  4159
p201N_cas9 : GGTGAATCTGGTGGGGGATGCTTCCATTGTCGAAAGTGCGCTGTTTGCGCAACAGATCTTCTCTGTTAAGCTTTACCAGCAGCTCCTCGGTGCCGTCCATTTTTTCCAAGATGGGCTTAATAAATTTGTA :  3731
             GGTGAATCTGGTGGGGGATGCTTCCATTGTCGAAAGTGCGCTGTTTGCGCAACAGATCTTCTCTGTTAAGCTTTACCAGCAGCTCCTCGGTGCCGTCCATTTTTTCCAAGATGGGCTTAATAAATTTGTA        
                                                                                                                                                       
                      *      4180         *      4200         *      4220         *      4240         *      4260         *      4280         *        
NODE_6452_ : AAATTCCTCCTGGCTTGCTCCGCCGTCAATGTATCCGGCGTAGCCATTTTTAGACTGATCGAAGAAAATTTCCTTGTACTTCTCAGGCAGTTGCTGTCTGACAAGGGCCTTCAGCAAAGTCAAGTCTTGG :  4289
p201N_cas9 : AAATTCCTCCTGGCTTGCTCCGCCGTCAATGTATCCGGCGTAGCCATTTTTAGACTGATCGAAGAAAATTTCCTTGTACTTCTCAGGCAGTTGCTGTCTGACAAGGGCCTTCAGCAAAGTCAAGTCTTGG :  3861
             AAATTCCTCCTGGCTTGCTCCGCCGTCAATGTATCCGGCGTAGCCATTTTTAGACTGATCGAAGAAAATTTCCTTGTACTTCTCAGGCAGTTGCTGTCTGACAAGGGCCTTCAGCAAAGTCAAGTCTTGG        
                                                                                                                                                       
                   4300         *      4320         *      4340         *      4360         *      4380         *      4400         *      4420        
NODE_6452_ : TGGTGCTCATCATAGCGCTTGATCATACTAGCGCTCAGCGGAGCTTTGGTGATCTCCGTGTTCACTCGCAGAATATCACTCAGCAGAATGGCGTCTGACAGGTTCTTTGCCGCCAAAAAAAGGTCTGCGT :  4419
p201N_cas9 : TGGTGCTCATCATAGCGCTTGATCATACTAGCGCTCAGCGGAGCTTTGGTGATCTCCGTGTTCACTCGCAGAATATCACTCAGCAGAATGGCGTCTGACAGGTTCTTTGCCGCCAAAAAAAGGTCTGCGT :  3991
             TGGTGCTCATCATAGCGCTTGATCATACTAGCGCTCAGCGGAGCTTTGGTGATCTCCGTGTTCACTCGCAGAATATCACTCAGCAGAATGGCGTCTGACAGGTTCTTTGCCGCCAAAAAAAGGTCTGCGT        
                                                                                                                                                       
                      *      4440         *      4460         *      4480         *      4500         *      4520         *      4540         *        
NODE_6452_ : ACTGGTCGCCGATCTGGGCCAGCAGATTGTCGAGATCATCATCGTAGGTGTCTTTGCTCAGTTGAAGCTTGGCATCTTCGGCCAGGTCGAAGTTAGATTTAAAGTTGGGGGTCAGCCCGAGTGACAGGGC :  4549
p201N_cas9 : ACTGGTCGCCGATCTGGGCCAGCAGATTGTCGAGATCATCATCGTAGGTGTCTTTGCTCAGTTGAAGCTTGGCATCTTCGGCCAGGTCGAAGTTAGATTTAAAGTTGGGGGTCAGCCCGAGTGACAGGGC :  4121
             ACTGGTCGCCGATCTGGGCCAGCAGATTGTCGAGATCATCATCGTAGGTGTCTTTGCTCAGTTGAAGCTTGGCATCTTCGGCCAGGTCGAAGTTAGATTTAAAGTTGGGGGTCAGCCCGAGTGACAGGGC        
                                                                                                                                                       
                   4560         *      4580         *      4600         *      4620         *      4640         *      4660         *      4680        
NODE_6452_ : GATAAGATTACCAAACAGGCCGTTCTTCTTCTCCCCAGGGAGCTGTGCGATGAGGTTTTCGAGCCGCCGGGATTTGGACAGCCTAGCGCTCAGGATTGCTTTGGCGTCAACTCCGGATGCGTTGATCGGG :  4679
p201N_cas9 : GATAAGATTACCAAACAGGCCGTTCTTCTTCTCCCCAGGGAGCTGTGCGATGAGGTTTTCGAGCCGCCGGGATTTGGACAGCCTAGCGCTCAGGATTGCTTTGGCGTCAACTCCGGATGCGTTGATCGGG :  4251
             GATAAGATTACCAAACAGGCCGTTCTTCTTCTCCCCAGGGAGCTGTGCGATGAGGTTTTCGAGCCGCCGGGATTTGGACAGCCTAGCGCTCAGGATTGCTTTGGCGTCAACTCCGGATGCGTTGATCGGG        
                                                                                                                                                       
                      *      4700         *      4720         *      4740         *      4760         *      4780         *      4800         *        
NODE_6452_ : TTCTCTTCGAAAAGCTGATTGTAAGTCTGAACCAGTTGGATAAAGAGTTTGTCGACATCGCTGTTGTCTGGGTTCAGGTCCCCCTCGATGAGGAAGTGTCCCCGAAATTTGATCATATGCGCCAGCGCGA :  4809
p201N_cas9 : TTCTCTTCGAAAAGCTGATTGTAAGTCTGAACCAGTTGGATAAAGAGTTTGTCGACATCGCTGTTGTCTGGGTTCAGGTCCCCCTCGATGAGGAAGTGTCCCCGAAATTTGATCATATGCGCCAGCGCGA :  4381
             TTCTCTTCGAAAAGCTGATTGTAAGTCTGAACCAGTTGGATAAAGAGTTTGTCGACATCGCTGTTGTCTGGGTTCAGGTCCCCCTCGATGAGGAAGTGTCCCCGAAATTTGATCATATGCGCCAGCGCGA        
                                                                                                                                                       
                   4820         *      4840         *      4860         *      4880         *      4900         *      4920         *      4940        
NODE_6452_ : GATAGATCAACCGCAAGTCAGCCTTATCAGTACTGTCTACAAGCTTCTTCCTCAGATGATATATGGTTGGGTACTTTTCATGGTACGCCACCTCGTCCACGATATTGCCAAAGATTGGGTGGCGCTCGTG :  4939
p201N_cas9 : GATAGATCAACCGCAAGTCAGCCTTATCAGTACTGTCTACAAGCTTCTTCCTCAGATGATATATGGTTGGGTACTTTTCATGGTACGCCACCTCGTCCACGATATTGCCAAAGATTGGGTGGCGCTCGTG :  4511
             GATAGATCAACCGCAAGTCAGCCTTATCAGTACTGTCTACAAGCTTCTTCCTCAGATGATATATGGTTGGGTACTTTTCATGGTACGCCACCTCGTCCACGATATTGCCAAAGATTGGGTGGCGCTCGTG        
                                                                                                                                                       
                      *      4960         *      4980         *      5000         *      5020         *      5040         *      5060         *        
NODE_6452_ : CTTTTTATCCTCCTCCACCAAAAAGGACTCCTCCAGCCTATGGAAGAAAGAGTCATCCACCTTAGCCATCTCATTACTAAAGATCTCCTGCAGGTAGCAGATCCGATTCTTTCTGCGGGTATATCTGCGC :  5069
p201N_cas9 : CTTTTTATCCTCCTCCACCAAAAAGGACTCCTCCAGCCTATGGAAGAAAGAGTCATCCACCTTAGCCATCTCATTACTAAAGATCTCCTGCAGGTAGCAGATCCGATTCTTTCTGCGGGTATATCTGCGC :  4641
             CTTTTTATCCTCCTCCACCAAAAAGGACTCCTCCAGCCTATGGAAGAAAGAGTCATCCACCTTAGCCATCTCATTACTAAAGATCTCCTGCAGGTAGCAGATCCGATTCTTTCTGCGGGTATATCTGCGC        
                                                                                                                                                       
                   5080         *      5100         *      5120         *      5140         *      5160         *      5180         *      5200        
NODE_6452_ : CGTGCTGTTCTTTTGAGCCGCGTGGCTTCGGCCGTCTCCCCGGAGTCGAACAGGAGGGCGCCAATGAGGTTCTTCTTTATGCTGTGGCGATCGGTATTGCCCAGAACTTTGAATTTTTTGCTCGGCACCT :  5199
p201N_cas9 : CGTGCTGTTCTTTTGAGCCGCGTGGCTTCGGCCGTCTCCCCGGAGTCGAACAGGAGGGCGCCAATGAGGTTCTTCTTTATGCTGTGGCGATCGGTATTGCCCAGAACTTTGAATTTTTTGCTCGGCACCT :  4771
             CGTGCTGTTCTTTTGAGCCGCGTGGCTTCGGCCGTCTCCCCGGAGTCGAACAGGAGGGCGCCAATGAGGTTCTTCTTTATGCTGTGGCGATCGGTATTGCCCAGAACTTTGAATTTTTTGCTCGGCACCT        
                                                                                                                                                       
                      *      5220         *      5240         *      5260         *      5280         *      5300         *      5320         *        
NODE_6452_ : TGTACTCGTCCGTAATGACGGCCCAGCCGACGCTGTTTGTGCCGATATCGAGCCCAATGGAGTACTTCTTGTCCATGCTAGCGGTCGAGAGAGATAGATTTGTAGAGAGAGACTGGTGATTTCAGCGTGT :  5329
p201N_cas9 : TGTACTCGTCCGTAATGACGGCCCAGCCGACGCTGTTTGTGCCGATATCGAGCCCAATGGAGTACTTCTTGTCCATGCTAGCGGTCGAGAGAGATAGATTTGTAGAGAGAGACTGGTGATTTCAGCGTGT :  4901
             TGTACTCGTCCGTAATGACGGCCCAGCCGACGCTGTTTGTGCCGATATCGAGCCCAATGGAGTACTTCTTGTCCATGCTAGCGGTCGAGAGAGATAGATTTGTAGAGAGAGACTGGTGATTTCAGCGTGT        
                                                                                                                                                       
                   5340         *      5360         *      5380         *      5400         *      5420         *      5440         *      5460        
NODE_6452_ : CCTCTCCAAATGAAATGAACTTCCTTATATAGAGGAAGGGTCTTGCGAAGGATAGTGGGATTGTGCGTCATCCCTTACGTCAGTGGAGATATCACATCAATCCACTTGCTTTGAAGACGTGGTTGGAACG :  5459
p201N_cas9 : CCTCTCCAAATGAAATGAACTTCCTTATATAGAGGAAGGGTCTTGCGAAGGATAGTGGGATTGTGCGTCATCCCTTACGTCAGTGGAGATATCACATCAATCCACTTGCTTTGAAGACGTGGTTGGAACG :  5031
             CCTCTCCAAATGAAATGAACTTCCTTATATAGAGGAAGGGTCTTGCGAAGGATAGTGGGATTGTGCGTCATCCCTTACGTCAGTGGAGATATCACATCAATCCACTTGCTTTGAAGACGTGGTTGGAACG        
                                                                                                                                                       
                      *      5480         *      5500         *      5520         *      5540         *      5560         *      5580         *        
NODE_6452_ : TCTTCTTTTTCCACGATGCTCCTCGTGGGTGGGGGTCCATCTTTGGGACCACTGTCGGCAGAGGCATCTTGAACGATAGCCTTTCCTTTATCGCAATGATGGCATTTGTAGGTGCCACCTTCCTTTTCTA :  5589
p201N_cas9 : TCTTCTTTTTCCACGATGCTCCTCGTGGGTGGGGGTCCATCTTTGGGACCACTGTCGGCAGAGGCATCTTGAACGATAGCCTTTCCTTTATCGCAATGATGGCATTTGTAGGTGCCACCTTCCTTTTCTA :  5161
             TCTTCTTTTTCCACGATGCTCCTCGTGGGTGGGGGTCCATCTTTGGGACCACTGTCGGCAGAGGCATCTTGAACGATAGCCTTTCCTTTATCGCAATGATGGCATTTGTAGGTGCCACCTTCCTTTTCTA        
                                                                                                                                                       
                   5600         *      5620         *      5640         *      5660         *      5680         *      5700         *      5720        
NODE_6452_ : CTGTCCTTTTGATGAAGTGACAGATAGCTGGGCAATGGAATCCGAGGAGGTTTCCCGATATTACCCTTTGTTGAAAAGTCTCAATAGCCCTTTGGTCTTCTGAGACTGTATCTTTGATATTCTTGGAGTA :  5719
p201N_cas9 : CTGTCCTTTTGATGAAGTGACAGATAGCTGGGCAATGGAATCCGAGGAGGTTTCCCGATATTACCCTTTGTTGAAAAGTCTCAATAGCCCTTTGGTCTTCTGAGACTGTATCTTTGATATTCTTGGAGTA :  5291
             CTGTCCTTTTGATGAAGTGACAGATAGCTGGGCAATGGAATCCGAGGAGGTTTCCCGATATTACCCTTTGTTGAAAAGTCTCAATAGCCCTTTGGTCTTCTGAGACTGTATCTTTGATATTCTTGGAGTA        
                                                                                                                                                       
                      *      5740         *      5760         *      5780         *      5800         *      5820         *      5840         *        
NODE_6452_ : GACGAGAGTGTCGTGCTCCACCATGTT------------------------------------------------------------------------------------------------------- :  5746
p201N_cas9 : GACGAGAGTGTCGTGCTCCACCATGTTCACATCAATCCACTTGCTTTGAAGACGTGGTTGGAACGTCTTCTTTTTCCACGATGCTCCTCGTGGGTGGGGGTCCATCTTTGGGACCACTGTCGGCAGAGGC :  5421
             GACGAGAGTGTCGTGCTCCACCATGTT                                                                                                               
                                                                                                                                                       
                   5860         *      5880         *      5900         *      5920         *      5940         *      5960         *      5980        
NODE_6452_ : ---------------------------------------------------------------------------------------------------------------------------------- :     -
p201N_cas9 : ATCTTGAACGATAGCCTTTCCTTTATCGCAATGATGGCATTTGTAGGTGCCACCTTCCTTTTCTACTGTCCTTTTGATGAAGTGACAGATAGCTGGGCAATGGAATCCGAGGAGGTTTCCCGATATTACC :  5551
                                                                                                                                                       
                                                                                                                                                       
                      *      6000         *      6020         *      6040         *      6060         *      6080         *      6100         *        
NODE_6452_ : --------------------------------------------------------------------------------------------GGGCCCGGCGCGCCGAATTCTAGTGATCGATTACCCTG :  5784
p201N_cas9 : CTTTGTTGAAAAGTCTCAATAGCCCTTTGGTCTTCTGAGACTGTATCTTTGATATTCTTGGAGTAGACGAGAGTGTCGTGCTCCACCATGTTGGGCCCGGCGCGCCGAATTCTAGTGATCGATTACCCTG :  5681
                                                                                                         GGGCCCGGCGCGCCGAATTCTAGTGATCGATTACCCTG        
                                                                                                                                                       
                   6120         *      6140         *      6160         *      6180         *      6200         *      6220         *      6240        
NODE_6452_ : TTATCCCTAGATATCGATTTTGGCTACCTTAAGAGAGTCATGAATTGTAATACGACTCAAAAAAAAGCACCGACTCGGTGCCACTTTTTCAAGTTGATAACGGACTAGCCTTATTTTAACTTGCTATTTC :  5914
p201N_cas9 : TTATCCCTAGATATCGATTTTGGCTACCTTAAGAGAGTCATGAATTGTAATACGACTCAAAAAAAAGCACCGACTCGGTGCCACTTTTTCAAGTTGATAACGGACTAGCCTTATTTTAACTTGCTATTTC :  5811
             TTATCCCTAGATATCGATTTTGGCTACCTTAAGAGAGTCATGAATTGTAATACGACTCAAAAAAAAGCACCGACTCGGTGCCACTTTTTCAAGTTGATAACGGACTAGCCTTATTTTAACTTGCTATTTC        
                                                                                                                                                       
                      *      6260         *      6280         *      6300         *      6320         *      6340         *      6360         *        
NODE_6452_ : TAGCTCTAAAACTTCCGCAGACCTAAAGAGCCAAGCCTACTGGTTCGCTTGAAGTATTTAAACGCTGAAATATTTTATGTTTCAGACGATGTGGGACTAAAAAGCAAGTGTAATAGTAACATGCAAATCC :  6044
p201N_cas9 : TAGCTCTAAAAC--------------------AAGCCTACTGGTTCGCTTGAAGTATTTAAACGCTGAAATATTTTATGTTTCAGACGATGTGGGACTAAAAAGCAAGTGTAATAGTAACATGCAAATCC :  5921
             TAGCTCTAAAAC                    AAGCCTACTGGTTCGCTTGAAGTATTTAAACGCTGAAATATTTTATGTTTCAGACGATGTGGGACTAAAAAGCAAGTGTAATAGTAACATGCAAATCC        
                                                                                                                                                       
                   6380         *      6400         *      6420         *      6440         *      6460         *      6480         *      6500        
NODE_6452_ : TTCTCGACTTTCACCTACTGGGATGCATCTTCCTCTCAGTGAGTTTTGTTTTATTTCAGATTAAACCAAAGTTTGTAGCAGTGCACCTTGTCTTTGCAAAATGAACAAGTGATACAATCATCACATCCCA :  6174
p201N_cas9 : TTCTCGACTTTCACCTACTGGGATGCATCTTCCTCTCAGTGAGTTTTGTTTTATTTCAGATTAAACCAAAGTTTGTAGCAGTGCACCTTGTCTTTGCAAAATGAACAAGTGATACAATCATCACATCCCA :  6051
             TTCTCGACTTTCACCTACTGGGATGCATCTTCCTCTCAGTGAGTTTTGTTTTATTTCAGATTAAACCAAAGTTTGTAGCAGTGCACCTTGTCTTTGCAAAATGAACAAGTGATACAATCATCACATCCCA        
                                                                                                                                                       
                      *      6520         *      6540         *      6560         *      6580         *      6600         *      6620         *        
NODE_6452_ : AAAGGATGTGATAAATTTAAGACCAAATTCCACATGAGTCATCATTCCCAAGCCAGGAGCTGCACCTTTTTTCACCATCATATGCACCCAATTAAATGCCTCATTGATCATATAAGATAGGCATAAATTT :  6304
p201N_cas9 : AAAGGATGTGATAAATTTAAGACCAAATTCCACATGAGTCATCATTCCCAAGCCAGGAGCTGCACCTTTTTTCACCATCATATGCACCCAATTAAATGCCTCATTGATCATATAAGATAGGCATAAATTT :  6181
             AAAGGATGTGATAAATTTAAGACCAAATTCCACATGAGTCATCATTCCCAAGCCAGGAGCTGCACCTTTTTTCACCATCATATGCACCCAATTAAATGCCTCATTGATCATATAAGATAGGCATAAATTT        
                                                                                                                                                       
                   6640         *      6660         *      6680         *      6700         *      6720         *      6740         *      6760        
NODE_6452_ : CATCGAAGAGATTAATATCGAATAATCATATACATACTTTAAATACATAACAAATTTTAAATACATATATCTGGTATATAATTAATTTTTTAAAGTCATGAAGTATGTATCAAATACACATATGGAAAAA :  6434
p201N_cas9 : CATCGAAGAGATTAATATCGAATAATCATATACATACTTTAAATACATAACAAATTTTAAATACATATATCTGGTATATAATTAATTTTTTAAAGTCATGAAGTATGTATCAAATACACATATGGAAAAA :  6311
             CATCGAAGAGATTAATATCGAATAATCATATACATACTTTAAATACATAACAAATTTTAAATACATATATCTGGTATATAATTAATTTTTTAAAGTCATGAAGTATGTATCAAATACACATATGGAAAAA        
                                                                                                                                                       
                      *      6780         *      6800         *      6820         *      6840         *      6860         *      6880         *        
NODE_6452_ : ATTAACTATTCATAATTTAAAAAATAGAAAAGATACATCTAGTGAAATTAGGTGCATGTATCAAATACATTAGGAAAAGGGCATATATCTTGATCTAGATAATTAACGATTTTGATTTATGTATAATTTC :  6564
p201N_cas9 : ATTAACTATTCATAATTTAAAAAATAGAAAAGATACATCTAGTGAAATTAGGTGCATGTATCAAATACATTAGGAAAAGGGCATATATCTTGATCTAGATAATTAACGATTTTGATTTATGTATAATTTC :  6441
             ATTAACTATTCATAATTTAAAAAATAGAAAAGATACATCTAGTGAAATTAGGTGCATGTATCAAATACATTAGGAAAAGGGCATATATCTTGATCTAGATAATTAACGATTTTGATTTATGTATAATTTC        
                                                                                                                                                       
                   6900         *      6920         *      6940         *      6960         *      6980         *      7000         *      7020        
NODE_6452_ : CAAATGAAGGTTTATATCTACTTCAGAAATAACAATATACTTTTATCAGAACATTCAACAAAGTAACAACCAACTAGAGTGAAAAATACACATTGTTCTCTAGACATACAAAATTGAGAAAAGAATCTCA :  6694
p201N_cas9 : CAAATGAAGGTTTATATCTACTTCAGAAATAACAATATACTTTTATCAGAACATTCAACAAAGTAACAACCAACTAGAGTGAAAAATACACATTGTTCTCTAGACATACAAAATTGAGAAAAGAATCTCA :  6571
             CAAATGAAGGTTTATATCTACTTCAGAAATAACAATATACTTTTATCAGAACATTCAACAAAGTAACAACCAACTAGAGTGAAAAATACACATTGTTCTCTAGACATACAAAATTGAGAAAAGAATCTCA        
                                                                                                                                                       
                      *      7040         *      7060         *      7080         *      7100         *      7120         *      7140         *        
NODE_6452_ : AAATTTAGAGAAACAAATCTGAATTTCTAGAAGAAAAAAATAATTATGCACTTTGCTATTGCTCGAAAAATAAATGAAAGAAATTAGACTTTTTTAAAAGATGTTAGACTAGATATACTCAAAAGCTATT :  6824
p201N_cas9 : AAATTTAGAGAAACAAATCTGAATTTCTAGAAGAAAAAAATAATTATGCACTTTGCTATTGCTCGAAAAATAAATGAAAGAAATTAGACTTTTTTAAAAGATGTTAGACTAGATATACTCAAAAGCTATT :  6701
             AAATTTAGAGAAACAAATCTGAATTTCTAGAAGAAAAAAATAATTATGCACTTTGCTATTGCTCGAAAAATAAATGAAAGAAATTAGACTTTTTTAAAAGATGTTAGACTAGATATACTCAAAAGCTATT        
                                                                                                                                                       
                   7160         *      7180         *      7200         *      7220         *      7240         *      7260         *      7280        
NODE_6452_ : AAAGGAGTAATATTCTTCTTACATTAAGTATTTTAGTTACAGTCCTGTAATTAAAGACACATTTTAGATTGTATCTAAACTTAAATGTATCTAGAATACATATATTTGAATGCATCATATACATGTATCC :  6954
p201N_cas9 : AAAGGAGTAATATTCTTCTTACATTAAGTATTTTAGTTACAGTCCTGTAATTAAAGACACATTTTAGATTGTATCTAAACTTAAATGTATCTAGAATACATATATTTGAATGCATCATATACATGTATCC :  6831
             AAAGGAGTAATATTCTTCTTACATTAAGTATTTTAGTTACAGTCCTGTAATTAAAGACACATTTTAGATTGTATCTAAACTTAAATGTATCTAGAATACATATATTTGAATGCATCATATACATGTATCC        
                                                                                                                                                       
                      *      7300         *      7320         *      7340         *      7360         *      7380         *      7400         *        
NODE_6452_ : GACACACCAATTCTCATAAAAAACGTAATATCCTAAACTAATTTATCCTTCAAGTCAACTTAAGCCCAATATACATTTTCATCTCTAAAGGCCCAAGTGGCACAAAATGTCAGGCCCAATTACGAAGAAA :  7084
p201N_cas9 : GACACACCAATTCTCATAAAAAACGTAATATCCTAAACTAATTTATCCTTCAAGTCAACTTAAGCCCAATATACATTTTCATCTCTAAAGGCCCAAGTGGCACAAAATGTCAGGCCCAATTACGAAGAAA :  6961
             GACACACCAATTCTCATAAAAAACGTAATATCCTAAACTAATTTATCCTTCAAGTCAACTTAAGCCCAATATACATTTTCATCTCTAAAGGCCCAAGTGGCACAAAATGTCAGGCCCAATTACGAAGAAA        
                                                                                                                                                       
                   7420         *      7440         *      7460         *      7480         *      7500         *      7520         *      7540        
NODE_6452_ : AGGGCTTGTAAAACCCTAATAAAGTGGCACTGGCAGAGCTTACACTCTCATTCCATCAACAAAGAAACCCTAAAAGCCGCAGCGCCACTGATTTCTCTCCTCCAGGCGAAGGATCGATCTTCGAATGCAT :  7214
p201N_cas9 : AGGGCTTGTAAAACCCTAATAAAGTGGCACTGGCAGAGCTTACACTCTCATTCCATCAACAAAGAAACCCTAAAAGCCGCAGCGCCACTGATTTCTCTCCTCCAGGCGAAGGATCGATCTTCGAATGCAT :  7091
             AGGGCTTGTAAAACCCTAATAAAGTGGCACTGGCAGAGCTTACACTCTCATTCCATCAACAAAGAAACCCTAAAAGCCGCAGCGCCACTGATTTCTCTCCTCCAGGCGAAGGATCGATCTTCGAATGCAT        
                                                                                                                                                       
                      *      7560         *      7580         *      7600         *      7620         *      7640         *      7660         *        
NODE_6452_ : CGCGCGGTACGTCTCGAGATGATTGAACAAGATGGATTGCACGCAGGTTCTCCGGCCGCTTGGGTGGAGAGGCTATTCGGCTATGACTGGGCACAACAGACAATCGGCTGCTCTGATGCCGCCGTGTTCC :  7344
p201N_cas9 : CGCGCGGTACGTCTCGAGATGATTGAACAAGATGGATTGCACGCAGGTTCTCCGGCCGCTTGGGTGGAGAGGCTATTCGGCTATGACTGGGCACAACAGACAATCGGCTGCTCTGATGCCGCCGTGTTCC :  7221
             CGCGCGGTACGTCTCGAGATGATTGAACAAGATGGATTGCACGCAGGTTCTCCGGCCGCTTGGGTGGAGAGGCTATTCGGCTATGACTGGGCACAACAGACAATCGGCTGCTCTGATGCCGCCGTGTTCC        
                                                                                                                                                       
                   7680         *      7700         *      7720         *      7740         *      7760         *      7780         *      7800        
NODE_6452_ : GGCTGTCAGCGCAGGGGCGCCCGGTTCTTTTTGTCAAGACCGACCTGTCCGGTGCCCTGAATGAACTGCAGGACGAGGCAGCGCGGCTATCGTGGCTGGCCACGACGGGCGTTCCTTGCGCAGCTGTGCT :  7474
p201N_cas9 : GGCTGTCAGCGCAGGGGCGCCCGGTTCTTTTTGTCAAGACCGACCTGTCCGGTGCCCTGAATGAACTGCAGGACGAGGCAGCGCGGCTATCGTGGCTGGCCACGACGGGCGTTCCTTGCGCAGCTGTGCT :  7351
             GGCTGTCAGCGCAGGGGCGCCCGGTTCTTTTTGTCAAGACCGACCTGTCCGGTGCCCTGAATGAACTGCAGGACGAGGCAGCGCGGCTATCGTGGCTGGCCACGACGGGCGTTCCTTGCGCAGCTGTGCT        
                                                                                                                                                       
                      *      7820         *      7840         *      7860         *      7880         *      7900         *      7920         *        
NODE_6452_ : CGACGTTGTCACTGAAGCGGGAAGGGACTGGCTGCTATTGGGCGAAGTGCCGGGGCAGGATCTCCTGTCATCTCACCTTGCTCCTGCCGAGAAAGTATCCATCATGGCTGATGCAATGCGGCGGCTGCAT :  7604
p201N_cas9 : CGACGTTGTCACTGAAGCGGGAAGGGACTGGCTGCTATTGGGCGAAGTGCCGGGGCAGGATCTCCTGTCATCTCACCTTGCTCCTGCCGAGAAAGTATCCATCATGGCTGATGCAATGCGGCGGCTGCAT :  7481
             CGACGTTGTCACTGAAGCGGGAAGGGACTGGCTGCTATTGGGCGAAGTGCCGGGGCAGGATCTCCTGTCATCTCACCTTGCTCCTGCCGAGAAAGTATCCATCATGGCTGATGCAATGCGGCGGCTGCAT        
                                                                                                                                                       
                   7940         *      7960         *      7980         *      8000         *      8020         *      8040         *      8060        
NODE_6452_ : ACGCTTGATCCGGCTACCTGCCCATTCGACCACCAAGCGAAACATCGCATCGAGCGAGCACGTACTCGGATGGAAGCCGGTCTTGTCGATCAGGATGATCTGGACGAAGAGCATCAGGGGCTCGCGCCAG :  7734
p201N_cas9 : ACGCTTGATCCGGCTACCTGCCCATTCGACCACCAAGCGAAACATCGCATCGAGCGAGCACGTACTCGGATGGAAGCCGGTCTTGTCGATCAGGATGATCTGGACGAAGAGCATCAGGGGCTCGCGCCAG :  7611
             ACGCTTGATCCGGCTACCTGCCCATTCGACCACCAAGCGAAACATCGCATCGAGCGAGCACGTACTCGGATGGAAGCCGGTCTTGTCGATCAGGATGATCTGGACGAAGAGCATCAGGGGCTCGCGCCAG        
                                                                                                                                                       
                      *      8080         *      8100         *      8120         *      8140         *      8160         *      8180         *        
NODE_6452_ : CCGAACTGTTCGCCAGGCTCAAGGCGCGCATGCCCGACGGCGATGATCTCGTCGTGACCCATGGCGATGCCTGCTTGCCGAATATCATGGTGGAAAATGGCCGCTTTTCTGGATTCATCGACTGTGGCCG :  7864
p201N_cas9 : CCGAACTGTTCGCCAGGCTCAAGGCGCGCATGCCCGACGGCGATGATCTCGTCGTGACCCATGGCGATGCCTGCTTGCCGAATATCATGGTGGAAAATGGCCGCTTTTCTGGATTCATCGACTGTGGCCG :  7741
             CCGAACTGTTCGCCAGGCTCAAGGCGCGCATGCCCGACGGCGATGATCTCGTCGTGACCCATGGCGATGCCTGCTTGCCGAATATCATGGTGGAAAATGGCCGCTTTTCTGGATTCATCGACTGTGGCCG        
                                                                                                                                                       
                   8200         *      8220         *      8240         *      8260         *      8280         *      8300         *      8320        
NODE_6452_ : GCTGGGTGTGGCGGACCGCTATCAGGACATAGCGTTGGCTACCCGTGATATTGCTGAAGAGCTTGGCGGCGAATGGGCTGACCGCTTCCTCGTGCTTTACGGTATCGCCGCTCCCGATTCGCAGCGCATC :  7994
p201N_cas9 : GCTGGGTGTGGCGGACCGCTATCAGGACATAGCGTTGGCTACCCGTGATATTGCTGAAGAGCTTGGCGGCGAATGGGCTGACCGCTTCCTCGTGCTTTACGGTATCGCCGCTCCCGATTCGCAGCGCATC :  7871
             GCTGGGTGTGGCGGACCGCTATCAGGACATAGCGTTGGCTACCCGTGATATTGCTGAAGAGCTTGGCGGCGAATGGGCTGACCGCTTCCTCGTGCTTTACGGTATCGCCGCTCCCGATTCGCAGCGCATC        
                                                                                                                                                       
                      *      8340         *      8360         *      8380         *      8400         *      8420         *      8440         *        
NODE_6452_ : GCCTTCTATCGCCTTCTTGACGAGTTCTTCTGAGAATTCGGCTTGATCCAAATTTTGATTTTAATGTTTAGCAAATGTCCTATCAGTTTTCTCTTTTTGTCGAACGGTAATTTAGAGTTTTTTTTGCTAT :  8124
p201N_cas9 : GCCTTCTATCGCCTTCTTGACGAGTTCTTCTGAGAATTCGGCTTGATCCAAATTTTGATTTTAATGTTTAGCAAATGTCCTATCAGTTTTCTCTTTTTGTCGAACGGTAATTTAGAGTTTTTTTTGCTAT :  8001
             GCCTTCTATCGCCTTCTTGACGAGTTCTTCTGAGAATTCGGCTTGATCCAAATTTTGATTTTAATGTTTAGCAAATGTCCTATCAGTTTTCTCTTTTTGTCGAACGGTAATTTAGAGTTTTTTTTGCTAT        
                                                                                                                                                       
                   8460         *      8480         *      8500         *      8520         *      8540         *      8560         *      8580        
NODE_6452_ : ATGGATTTTCGTTTTTGATGTATGTGACAACCCTCGGGATTGTTGATTTATTTCAAAACTAAGAGTTTTTGCTTATTGTTCTCGTCTATTTTGGATATCAATCTTAGTTTTATATCTTTTCTAGTTCTCT :  8254
p201N_cas9 : ATGGATTTTCGTTTTTGATGTATGTGACAACCCTCGGGATTGTTGATTTATTTCAAAACTAAGAGTTTTTGCTTATTGTTCTCGTCTATTTTGGATATCAATCTTAGTTTTATATCTTTTCTAGTTCTCT :  8131
             ATGGATTTTCGTTTTTGATGTATGTGACAACCCTCGGGATTGTTGATTTATTTCAAAACTAAGAGTTTTTGCTTATTGTTCTCGTCTATTTTGGATATCAATCTTAGTTTTATATCTTTTCTAGTTCTCT        
                                                                                                                                                       
                      *      8600         *      8620         *      8640         *      8660         *      8680         *      8700         *        
NODE_6452_ : ACGTGTTAAATGTTCAACACACTAGCAATTTGGCTGCAGCGTATGGATTATGGAACTATCAAGTCTGTGGGATCGATAAATATGCTTCTCAGGAATTTGAGATTTTACAGTCTTTATGCTCATTGGGTTG :  8384
p201N_cas9 : ACGTGTTAAATGTTCAACACACTAGCAATTTGGCTGCAGCGTATGGATTATGGAACTATCAAGTCTGTGGGATCGATAAATATGCTTCTCAGGAATTTGAGATTTTACAGTCTTTATGCTCATTGGGTTG :  8261
             ACGTGTTAAATGTTCAACACACTAGCAATTTGGCTGCAGCGTATGGATTATGGAACTATCAAGTCTGTGGGATCGATAAATATGCTTCTCAGGAATTTGAGATTTTACAGTCTTTATGCTCATTGGGTTG        
                                                                                                                                                       
                   8720         *      8740         *      8760         *      8780         *      8800         *      8820         *      8840        
NODE_6452_ : AGTATAATATAGTAAAAAAATAGTAAATTTAAGCAATAATGTTAGGTGCTATGTGTCTGTCGATACCAT-------CATCATGGATCTATCCA-TCACAAGTATT-----ATGATGGAAT--------AT :  8493
p201N_cas9 : AGTATAATATAGTAAAAAAATAGTAAATTTAAGCAATAATGTTAGGTGCTATGTGTCTGTCGAGACTATTGGGGTACGGCGCGGCTTAATTAAGCCGCGCCTACCCTCTAGTCAAGGAATTCGTAATCAT :  8391
             AGTATAATATAGTAAAAAAATAGTAAATTTAAGCAATAATGTTAGGTGCTATGTGTCTGTCGA AC AT       C  C  GG T  AT  A  C C   TA        T A GGAAT        AT        
                                                                                                                                                       
                      *      8860         *      8880         *      8900         *      8920         *      8940         *      8960         *        
NODE_6452_ : TTTCTACTTTATCAGTACACCAAA--AATACCCACATGTAAA---ATGAGGGTTATGGGTCCA---TGTATAGGGATATATCTATGGC------CAAATTTAATGTCCTACTTTATTTATAATGCATTAT :  8609
p201N_cas9 : GTCATAGCTGTTTCCTGTGTGAAATTGTTATCCGCTCACAATTCCACACAACATACGAGCCGGAAGCATAAAGTGTAAAGCCTGGGGTGCCTAATGAGTGAGCTAACTCACATTAATTGCGTTGCGCTCA :  8521
              T  TA  T  T   T     AAA    TA CC C    AA    A       TA G G C       TA AG G  A   CT  GG         A T    T  C  AC TTA TT    TGC  T          
                                                                                                                                                       
                   8980         *      9000         *      9020         *      9040         *      9060         *      9080         *      9100        
NODE_6452_ : TTGCATACGGATTTGTGTGCAAATAAAAGGGGAAATATTTGTCATT-ATTATGCCTATATTTTGAAAGTTTCAAA--ACTTTTCAAGTTAAATCTTAAGGCTATATCTGATTCC--CGAAAATTTGAAGA :  8734
p201N_cas9 : CTGCCCGCTTTCCAGTCGGGAAACCTGTCGTGCCAGCTGCATTAATGAATCGGCCAACGCGCGGGGAGAGGCGGTTTGCGTATTGGCTAGAGCAATTCGGCGTTAATTCAGTACATTAAAAACGTCCGCA :  8651
              TGC   C      GT  G AAA      G G  A  T   T A T A T  GCC A      G  AG   C      C T T    T  A    T  GGC  TA  T A T C    AAAA  T    A        
                                                                                                                                                       
                      *      9120         *      9140         *      9160         *      9180         *      9200         *      9220         *        
NODE_6452_ : CTATGTTA---GGTTCCTGAAAAGT------ATCGAGGAAAGAAAAAAAAGTGTTAAAA---AAATAATTTTTTTATGTTTGGT--TTCACTATAAAA----------ATACAAA-AGAAAATCAAATAT :  8839
p201N_cas9 : ATGTGTTATTAAGTTGTCTAAGCGTCAATTTGTTTACACCACAATATATCCTGCCACCAGCCAGCCAACAGCTCCCCGACCGGCAGCTCGGCACAAAATCACCACTCGATACAGGCAGCCCATCAGTCCG :  8781
              T TGTTA    GTT    AA  GT       T  A    A AA A A   TG  A  A   A   AA    T    G   GG    TC   A AAAA          ATACA   AG   ATCA             
                                                                                                                                                       
                   9240         *      9260         *      9280         *      9300         *      9320         *      9340         *      9360        
NODE_6452_ : GATTAAAATTTGTCCAAAAT---TTATATATATTTAAATTATTTTAATCTTT---ATATAATAGAGGGAAATAATAAAATAAGCTTGAAAAAAC-ATATAGAAATAATTT-GTTGAGTTTAA-ATATTTT :  8960
p201N_cas9 : GGACGGCGTCAGCGGGAGAGCCGTTGTAAGGCGGCAGACTTTGCTCATGTTACCGATGCTATTCGGAAGAACGGCAAC-TAAGCTGCCGGGTTTGAAACACGGATGATCTCGCGGAGGGTAGCATGTTGA :  8910
             G       T  G    A A    TT TA       A A T T  T AT TT    AT   AT   G   AA    AA  TAAGCT          A A A   AT AT T G  GAG  TA  AT TT          
                                                                                                                                                       
                      *      9380         *      9400         *      9420         *      9440         *      9460         *      9480         *        
NODE_6452_ : TTTTTCT--TCAC----TTTTCTTTCCTTTTATTTATATTTTTTCT--TCAAATTTTTTGAGAAC-TAAACA-TAGCCTAAGTGAAA------------------------------------------- :  9037
p201N_cas9 : TTGTAACGATGACAGAGCGTTGCTGCCTGTGATCCAGATCATGAACAATAAAACTGTCTGCTTACATAAACAGTAATACAAGGGGTGTTATGAGCCATATTCAACGGGAAACGTCTTGCTCTAGGCCGCG :  9040
             TT T     T AC      TT  T CCT T AT  A AT  T      T AAA T T TG   AC TAAACA TA    AAG G                                                      
                                                                                                                                                       
                   9500         *      9520         *      9540         *      9560         *      9580         *      9600         *      9620        
NODE_6452_ : ---------------------------------------------------------------------------------------------------------------------------------- :     -
p201N_cas9 : ATTAAATTCCAACATGGATGCTGATTTATATGGGTATAAATGGGCTCGCGATAATGTCGGGCAATCAGGTGCGACAATCTATCGATTGTATGGGAAGCCCGATGCGCCAGAGTTGTTTCTGAAACATGGC :  9170
                                                                                                                                                       
                                                                                                                                                       
                      *      9640         *      9660         *      9680         *      9700         *      9720         *      9740         *        
NODE_6452_ : ---------------------------------------------------------------------------------------------------------------------------------- :     -
p201N_cas9 : AAAGGTAGCGTTGCCAATGATGTTACAGATGAGATGGTCAGACTAAACTGGCTGACGGAATTTATGCCTCTTCCGACCATCAAGCATTTTATCCGTACTCCTGATGATGCATGGTTACTCACCACTGCGA :  9300
                                                                                                                                                       
                                                                                                                                                       
                   9760         *      9780         *      9800         *      9820         *      9840         *      9860         *      9880        
NODE_6452_ : ---------------------------------------------------------------------------------------------------------------------------------- :     -
p201N_cas9 : TCCCCGGGAAAACAGCATTCCAGGTATTAGAAGAATATCCTGATTCAGGTGAAAATATTGTTGATGCGCTGGCAGTGTTCCTGCGCCGGTTGCATTCGATTCCTGTTTGTAATTGTCCTTTTAACAGCGA :  9430
                                                                                                                                                       
                                                                                                                                                       
                      *      9900         *      9920         *      9940         *      9960         *      9980         *     10000         *        
NODE_6452_ : ---------------------------------------------------------------------------------------------------------------------------------- :     -
p201N_cas9 : TCGCGTATTTCGTCTCGCTCAGGCGCAATCACGAATGAATAACGGTTTGGTTGATGCGAGTGATTTTGATGACGAGCGTAATGGCTGGCCTGTTGAACAAGTCTGGAAAGAAATGCATAAACTTTTGCCA :  9560
                                                                                                                                                       
                                                                                                                                                       
                  10020         *     10040         *     10060         *     10080         *     10100         *     10120         *     10140        
NODE_6452_ : ---------------------------------------------------------------------------------------------------------------------------------- :     -
p201N_cas9 : TTCTCACCGGATTCAGTCGTCACTCATGGTGATTTCTCACTTGATAACCTTATTTTTGACGAGGGGAAATTAATAGGTTGTATTGATGTTGGACGAGTCGGAATCGCAGACCGATACCAGGATCTTGCCA :  9690
                                                                                                                                                       
                                                                                                                                                       
                      *     10160         *     10180         *     10200         *     10220         *     10240         *     10260         *        
NODE_6452_ : ---------------------------------------------------------------------------------------------------------------------------------- :     -
p201N_cas9 : TCCTATGGAACTGCCTCGGTGAGTTTTCTCCTTCATTACAGAAACGGCTTTTTCAAAAATATGGTATTGATAATCCTGATATGAATAAATTGCAGTTTCATTTGATGCTCGATGAGTTTTTCTAAGAATT :  9820
                                                                                                                                                       
                                                                                                                                                       
                  10280         *     10300         *     10320         *     10340         *     10360         *     10380         *     10400        
NODE_6452_ : ---------------------------------------------------------------------------------------------------------------------------------- :     -
p201N_cas9 : AATTCATGATCCTGCATGACCAAAATCCCTTAACGTGAGTTTTCGTTCCACTGAGCGTCAGACCCCGTAGAAAAGATCAAAGGATCTTCTTGAGATCCTTTTTTTCTGCGCGTAATCTGCTGCTTGCAAA :  9950
                                                                                                                                                       
                                                                                                                                                       
                      *     10420         *     10440         *     10460         *     10480         *     10500         *     10520         *        
NODE_6452_ : ---------------------------------------------------------------------------------------------------------------------------------- :     -
p201N_cas9 : CAAAAAAACCACCGCTACCAGCGGTGGTTTGTTTGCCGGATCAAGAGCTACCAACTCTTTTTCCGAAGGTAACTGGCTTCAGCAGAGCGCAGATACCAAATACTGTCCTTCTAGTGTAGCCGTAGTTAGG : 10080
                                                                                                                                                       
                                                                                                                                                       
                  10540         *     10560         *     10580         *     10600         *     10620         *     10640         *     10660        
NODE_6452_ : ---------------------------------------------------------------------------------------------------------------------------------- :     -
p201N_cas9 : CCACCACTTCAAGAACTCTGTAGCACCGCCTACATACCTCGCTCTGCTAATCCTGTTACCAGTGGCTGCTGCCAGTGGCGATAAGTCGTGTCTTACCGGGTTGGACTCAAGACGATAGTTACCGGATAAG : 10210
                                                                                                                                                       
                                                                                                                                                       
                      *     10680         *     10700         *     10720         *     10740         *     10760         *     10780         *        
NODE_6452_ : ---------------------------------------------------------------------------------------------------------------------------------- :     -
p201N_cas9 : GCGCAGCGGTCGGGCTGAACGGGGGGTTCGTGCACACAGCCCAGCTTGGAGCGAACGACCTACACCGAACTGAGATACCTACAGCGTGAGCTATGAGAAAGCGCCACGCTTCCCGAAGGGAGAAAGGCGG : 10340
                                                                                                                                                       
                                                                                                                                                       
                  10800         *     10820         *     10840         *     10860         *     10880         *     10900         *     10920        
NODE_6452_ : ---------------------------------------------------------------------------------------------------------------------------------- :     -
p201N_cas9 : ACAGGTATCCGGTAAGCGGCAGGGTCGGAACAGGAGAGCGCACGAGGGAGCTTCCAGGGGGAAACGCCTGGTATCTTTATAGTCCTGTCGGGTTTCGCCACCTCTGACTTGAGCGTCGATTTTTGTGATG : 10470
                                                                                                                                                       
                                                                                                                                                       
                      *     10940         *     10960         *     10980         *     11000         *     11020         *     11040         *        
NODE_6452_ : ---------------------------------------------------------------------------------------------------------------------------------- :     -
p201N_cas9 : CTCGTCAGGGGGGCGGAGCCTATGGAAAAACGCCAGCAACGCGGCCTTTTTACGGTTCCTGGCCTTTTGCTGGCCTTTTGCTCACATGTTCTTTCCTGCGTTATCCCCTGATTCTGTGGATAACCGTATT : 10600
                                                                                                                                                       
                                                                                                                                                       
                  11060         *     11080         *     11100         *     11120         *     11140         *     11160         *     11180        
NODE_6452_ : ---------------------------------------------------------------------------------------------------------------------------------- :     -
p201N_cas9 : ACCGCCTTTGAGTGAGCTGATACCGCTCGCCGCAGCCGAACGACCGAGCGCAGCGAGTCAGTGAGCGAGGAAGCGGAAGAGCGCCTGATGCGGTATTTTCTCCTTACGCATCTGTGCGGTATTTCACACC : 10730
                                                                                                                                                       
                                                                                                                                                       
                      *     11200         *     11220         *     11240         *     11260         *     11280         *     11300         *        
NODE_6452_ : ---------------------------------------------------------------------------------------------------------------------------------- :     -
p201N_cas9 : GCATATGGTGCACTCTCAGTACAATCTGCTCTGATGCCGCATAGTTAAGCCAGTATACACTCCGCTATCGCTACGTGACTGGGTCATGGCTGCGCCCCGACACCCGCCAACACCCGCTGACGCGCCCTGA : 10860
                                                                                                                                                       
                                                                                                                                                       
                  11320         *     11340         *     11360         *     11380         *     11400         *     11420         *     11440        
NODE_6452_ : ---------------------------------------------------------------------------------------------------------------------------------- :     -
p201N_cas9 : CGGGCTTGTCTGCTCCCGGCATCCGCTTACAGACAAGCTGTGACCGTCTCCGGGAGCTGCATGTGTCAGAGGTTTTCACCGTCATCACCGAAACGCGCGAGGCAGGGTGCCTTGATGTGGGCGCCGGCGG : 10990
                                                                                                                                                       
                                                                                                                                                       
                      *     11460         *     11480         *     11500         *     11520         *     11540         *     11560         *        
NODE_6452_ : ---------------------------------------------------------------------------------------------------------------------------------- :     -
p201N_cas9 : TCGAGTGGCGACGGCGCGGCTTGTCCGCGCCCTGGTAGATTGCCTGGCCGTAGGCCAGCCATTTTTGAGCGGCCAGCGGCCGCGATAGGCCGACGCGAAGCGGCGGGGCGTAGGGAGCGCAGCGACCGAA : 11120
                                                                                                                                                       
                                                                                                                                                       
                  11580         *     11600         *     11620         *     11640         *     11660         *     11680         *     11700        
NODE_6452_ : ---------------------------------------------------------------------------------------------------------------------------------- :     -
p201N_cas9 : GGGTAGGCGCTTTTTGCAGCTCTTCGGCTGTGCGCTGGCCAGACAGTTATGCACAGGCCAGGCGGGTTTTAAGAGTTTTAATAAGTTTTAAAGAGTTTTAGGCGGAAAAATCGCCTTTTTTCTCTTTTAT : 11250
                                                                                                                                                       
                                                                                                                                                       
                      *     11720         *     11740         *     11760         *     11780         *     11800         *     11820         *        
NODE_6452_ : ---------------------------------------------------------------------------------------------------------------------------------- :     -
p201N_cas9 : ATCAGTCACTTACATGTGTGACCGGTTCCCAATGTACGGCTTTGGGTTCCCAATGTACGGGTTCCGGTTCCCAATGTACGGCTTTGGGTTCCCAATGTACGTGCTATCCACAGGAAAGAGACCTTTTCGA : 11380
                                                                                                                                                       
                                                                                                                                                       
                  11840         *     11860         *     11880         *     11900         *     11920         *     11940         *     11960        
NODE_6452_ : ---------------------------------------------------------------------------------------------------------------------------------- :     -
p201N_cas9 : CCTTTTTCCCCTGCTAGGGCAATTTGCCCTAGCATCTGCTCCGTACATTAGGAACCGGCGGATGCTTCGCCCTCGATCAGGTTGCGGTAGCGCATGACTAGGATCGGGCCAGCCTGCCCCGCCTCCTCCT : 11510
                                                                                                                                                       
                                                                                                                                                       
                      *     11980         *     12000         *     12020         *     12040         *     12060         *     12080         *        
NODE_6452_ : ---------------------------------------------------------------------------------------------------------------------------------- :     -
p201N_cas9 : TCAAATCGTACTCCGGCAGGTCATTTGACCCGATCAGCTTGCGCACGGTGAAACAGAACTTCTTGAACTCTCCGGCGCTGCCACTGCGTTCGTAGATCGTCTTGAACAACCATCTGGCTTCTGCCTTGCC : 11640
                                                                                                                                                       
                                                                                                                                                       
                  12100         *     12120         *     12140         *     12160         *     12180         *     12200         *     12220        
NODE_6452_ : ---------------------------------------------------------------------------------------------------------------------------------- :     -
p201N_cas9 : TGCGGCGCGGCGTGCCAGGCGGTAGAGAAAACGGCCGATGCCGGGATCGATCAAAAAGTAATCGGGGTGAACCGTCAGCACGTCCGGGTTCTTGCCTTCTGTGATCTCGCGGTACATCCAATCAGCTAGC : 11770
                                                                                                                                                       
                                                                                                                                                       
                      *     12240         *     12260         *     12280         *     12300         *     12320         *     12340         *        
NODE_6452_ : ---------------------------------------------------------------------------------------------------------------------------------- :     -
p201N_cas9 : TCGATCTCGATGTACTCCGGCCGCCCGGTTTCGCTCTTTACGATCTTGTAGCGGCTAATCAAGGCTTCACCCTCGGATACCGTCACCAGGCGGCCGTTCTTGGCCTTCTTCGTACGCTGCATGGCAACGT : 11900
                                                                                                                                                       
                                                                                                                                                       
                  12360         *     12380         *     12400         *     12420         *     12440         *     12460         *     12480        
NODE_6452_ : ---------------------------------------------------------------------------------------------------------------------------------- :     -
p201N_cas9 : GCGTGGTGTTTAACCGAATGCAGGTTTCTACCAGGTCGTCTTTCTGCTTTCCGCCATCGGCTCGCCGGCAGAACTTGAGTACGTCCGCAACGTGTGGACGGAACACGCGGCCGGGCTTGTCTCCCTTCCC : 12030
                                                                                                                                                       
                                                                                                                                                       
                      *     12500         *     12520         *     12540         *     12560         *     12580         *     12600         *        
NODE_6452_ : ---------------------------------------------------------------------------------------------------------------------------------- :     -
p201N_cas9 : TTCCCGGTATCGGTTCATGGATTCGGTTAGATGGGAAACCGCCATCAGTACCAGGTCGTAATCCCACACACTGGCCATGCCGGCCGGCCCTGCGGAAACCTCTACGTGCCCGTCTGGAAGCTCGTAGCGG : 12160
                                                                                                                                                       
                                                                                                                                                       
                  12620         *     12640         *     12660         *     12680         *     12700         *     12720         *     12740        
NODE_6452_ : ---------------------------------------------------------------------------------------------------------------------------------- :     -
p201N_cas9 : ATCACCTCGCCAGCTCGTCGGTCACGCTTCGACAGACGGAAAACGGCCACGTCCATGATGCTGCGACTATCGCGGGTGCCCACGTCATAGAGCATCGGAACGAAAAAATCTGGTTGCTCGTCGCCCTTGG : 12290
                                                                                                                                                       
                                                                                                                                                       
                      *     12760         *     12780         *     12800         *     12820         *     12840         *     12860         *        
NODE_6452_ : ---------------------------------------------------------------------------------------------------------------------------------- :     -
p201N_cas9 : GCGGCTTCCTAATCGACGGCGCACCGGCTGCCGGCGGTTGCCGGGATTCTTTGCGGATTCGATCAGCGGCCGCTTGCCACGATTCACCGGGGCGTGCTTCTGCCTCGATGCGTTGCCGCTGGGCGGCCTG : 12420
                                                                                                                                                       
                                                                                                                                                       
                  12880         *     12900         *     12920         *     12940         *     12960         *     12980         *     13000        
NODE_6452_ : ---------------------------------------------------------------------------------------------------------------------------------- :     -
p201N_cas9 : CGCGGCCTTCAACTTCTCCACCAGGTCATCACCCAGCGCCGCGCCGATTTGTACCGGGCCGGATGGTTTGCGACCGTCACGCCGATTCCTCGGGCTTGGGGGTTCCAGTGCCATTGCAGGGCCGGCAGAC : 12550
                                                                                                                                                       
                                                                                                                                                       
                      *     13020         *     13040         *     13060         *     13080         *     13100         *     13120         *        
NODE_6452_ : ---------------------------------------------------------------------------------------------------------------------------------- :     -
p201N_cas9 : AACCCAGCCGCTTACGCCTGGCCAACCGCCCGTTCCTCCACACATGGGGCATTCCACGGCGTCGGTGCCTGGTTGTTCTTGATTTTCCATGCCGCCTCCTTTAGCCGCTAAAATTCATCTACTCATTTAT : 12680
                                                                                                                                                       
                                                                                                                                                       
                  13140         *     13160         *     13180         *     13200         *     13220         *     13240         *     13260        
NODE_6452_ : ---------------------------------------------------------------------------------------------------------------------------------- :     -
p201N_cas9 : TCATTTGCTCATTTACTCTGGTAGCTGCGCGATGTATTCAGATAGCAGCTCGGTAATGGTCTTGCCTTGGCGTACCGCGTACATCTTCAGCTTGGTGTGATCCTCCGCCGGCAACTGAAAGTTGACCCGC : 12810
                                                                                                                                                       
                                                                                                                                                       
                      *     13280         *     13300         *     13320         *     13340         *     13360         *     13380         *        
NODE_6452_ : ---------------------------------------------------------------------------------------------------------------------------------- :     -
p201N_cas9 : TTCATGGCTGGCGTGTCTGCCAGGCTGGCCAACGTTGCAGCCTTGCTGCTGCGTGCGCTCGGACGGCCGGCACTTAGCGTGTTTGTGCTTTTGCTCATTTTCTCTTTACCTCATTAACTCAAATGAGTTT : 12940
                                                                                                                                                       
                                                                                                                                                       
                  13400         *     13420         *     13440         *     13460         *     13480         *     13500         *     13520        
NODE_6452_ : ---------------------------------------------------------------------------------------------------------------------------------- :     -
p201N_cas9 : TGATTTAATTTCAGCGGCCAGCGCCTGGACCTCGCGGGCAGCGTCGCCCTCGGGTTCTGATTCAAGAACGGTTGTGCCGGCGGCGGCAGTGCCTGGGTAGCTCACGCGCTGCGTGATACGGGACTCAAGA : 13070
                                                                                                                                                       
                                                                                                                                                       
                      *     13540         *     13560         *     13580         *     13600         *     13620         *     13640         *        
NODE_6452_ : ---------------------------------------------------------------------------------------------------------------------------------- :     -
p201N_cas9 : ATGGGCAGCTCGTACCCGGCCAGCGCCTCGGCAACCTCACCGCCGATGCGCGTGCCTTTGATCGCCCGCGACACGACAAAGGCCGCTTGTAGCCTTCCATCCGTGACCTCAATGCGCTGCTTAACCAGCT : 13200
                                                                                                                                                       
                                                                                                                                                       
                  13660         *     13680         *     13700         *     13720         *     13740         *     13760         *     13780        
NODE_6452_ : ---------------------------------------------------------------------------------------------------------------------------------- :     -
p201N_cas9 : CCACCAGGTCGGCGGTGGCCCATATGTCGTAAGGGCTTGGCTGCACCGGAATCAGCACGAAGTCGGCTGCCTTGATCGCGGACACAGCCAAGTCCGCCGCCTGGGGCGCTCCGTCGATCACTACGAAGTC : 13330
                                                                                                                                                       
                                                                                                                                                       
                      *     13800         *     13820         *     13840         *     13860         *     13880         *     13900         *        
NODE_6452_ : ---------------------------------------------------------------------------------------------------------------------------------- :     -
p201N_cas9 : GCGCCGGCCGATGGCCTTCACGTCGCGGTCAATCGTCGGGCGGTCGATGCCGACAACGGTTAGCGGTTGATCTTCCCGCACGGCCGCCCAATCGCGGGCACTGCCCTGGGGATCGGAATCGACTAACAGA : 13460
                                                                                                                                                       
                                                                                                                                                       
                  13920         *     13940         *     13960         *     13980         *     14000         *     14020         *     14040        
NODE_6452_ : ---------------------------------------------------------------------------------------------------------------------------------- :     -
p201N_cas9 : ACATCGGCCCCGGCGAGTTGCAGGGCGCGGGCTAGATGGGTTGCGATGGTCGTCTTGCCTGACCCGCCTTTCTGGTTAAGTACAGCGATAACCTTCATGCGTTCCCCTTGCGTATTTGTTTATTTACTCA : 13590
                                                                                                                                                       
                                                                                                                                                       
                      *     14060         *     14080         *     14100         *     14120         *     14140         *     14160         *        
NODE_6452_ : ---------------------------------------------------------------------------------------------------------------------------------- :     -
p201N_cas9 : TCGCATCATATACGCAGCGACCGCATGACGCAAGCTGTTTTACTCAAATACACATCACCTTTTTAGACGGCGGCGCTCGGTTTCTTCAGCGGCCAAGCTGGCCGGCCAGGCCGCCAGCTTGGCATCAGAC : 13720
                                                                                                                                                       
                                                                                                                                                       
                  14180         *     14200         *     14220         *     14240         *     14260         *     14280         *     14300        
NODE_6452_ : ---------------------------------------------------------------------------------------------------------------------------------- :     -
p201N_cas9 : AAACCGGCCAGGATTTCATGCAGCCGCACGGTTGAGACGTGCGCGGGCGGCTCGAACACGTACCCGGCCGCGATCATCTCCGCCTCGATCTCTTCGGTAATGAAAAACGGTTCGTCCTGGCCGTCCTGGT : 13850
                                                                                                                                                       
                                                                                                                                                       
                      *     14320         *     14340         *     14360         *     14380         *     14400         *     14420         *        
NODE_6452_ : ---------------------------------------------------------------------------------------------------------------------------------- :     -
p201N_cas9 : GCGGTTTCATGCTTGTTCCTCTTGGCGTTCATTCTCGGCGGCCGCCAGGGCGTCGGCCTCGGTCAATGCGTCCTCACGGAAGGCACCGCGCCGCCTGGCCTCGGTGGGCGTCACTTCCTCGCTGCGCTCA : 13980
                                                                                                                                                       
                                                                                                                                                       
                  14440         *     14460         *     14480         *     14500         *     14520         *     14540         *     14560        
NODE_6452_ : ---------------------------------------------------------------------------------------------------------------------------------- :     -
p201N_cas9 : AGTGCGCGGTACAGGGTCGAGCGATGCACGCCAAGCAGTGCAGCCGCCTCTTTCACGGTGCGGCCTTCCTGGTCGATCAGCTCGCGGGCGTGCGCGATCTGTGCCGGGGTGAGGGTAGGGCGGGGGCCAA : 14110
                                                                                                                                                       
                                                                                                                                                       
                      *     14580         *     14600         *     14620         *     14640         *     14660         *     14680         *        
NODE_6452_ : ---------------------------------------------------------------------------------------------------------------------------------- :     -
p201N_cas9 : ACTTCACGCCTCGGGCCTTGGCGGCCTCGCGCCCGCTCCGGGTGCGGTCGATGATTAGGGAACGCTCGAACTCGGCAATGCCGGCGAACACGGTCAACACCATGCGGCCGGCCGGCGTGGTGGTGTCGGC : 14240
                                                                                                                                                       
                                                                                                                                                       
                  14700         *     14720         *     14740         *     14760         *     14780         *     14800         *     14820        
NODE_6452_ : ---------------------------------------------------------------------------------------------------------------------------------- :     -
p201N_cas9 : CCACGGCTCTGCCAGGCTACGCAGGCCCGCGCCGGCCTCCTGGATGCGCTCGGCAATGTCCAGTAGGTCGCGGGTGCTGCGGGCCAGGCGGTCTAGCCTGGTCACTGTCACAACGTCGCCAGGGCGTAGG : 14370
                                                                                                                                                       
                                                                                                                                                       
                      *     14840         *     14860         *     14880         *     14900         *     14920         *     14940         *        
NODE_6452_ : ---------------------------------------------------------------------------------------------------------------------------------- :     -
p201N_cas9 : TGGTCAAGCATCCTGGCCAGCTCCGGGCGGTCGCGCCTGGTGCCGGTGATCTTCTCGGAAAACAGCTTGGTGCAGCCGGCCGCGTGCAGTTCGGCCCGTTGGTTGGTCAAGTCCTGGTCGTCGGTGCTGA : 14500
                                                                                                                                                       
                                                                                                                                                       
                  14960         *     14980         *     15000         *     15020         *     15040         *     15060         *     15080        
NODE_6452_ : ---------------------------------------------------------------------------------------------------------------------------------- :     -
p201N_cas9 : CGCGGGCATAGCCCAGCAGGCCAGCGGCGGCGCTCTTGTTCATGGCGTAATGTCTCCGGTTCTAGTCGCAAGTATTCTACTTTATGCGACTAAAACACGCGACAAGAAAACGCCAGGAAAAGGGCAGGGC : 14630
                                                                                                                                                       
                                                                                                                                       
                      *     15100         *     15120         *     15140         *     15160         *     15180         *            
NODE_6452_ : ------------------------------------------------------------------------------------------------------------------ :     -
p201N_cas9 : GGCAGCCTGTCGCGTAACTTAGGACTTGTGCGACATGTCGTTTTCAGAAGACGGCTGCACTGAACGTCAGAAGCCGACTGCACTATAGCAGCGGAGGGGTTGGATCAAAGTACT : 14744
